# Author Correction: CpG island turnover events predict evolutionary changes in enhancer activity

**DOI:** 10.1186/s13059-025-03834-w

**Published:** 2025-10-22

**Authors:** Acadia A. Kocher, Emily V. Dutrow, Severin Uebbing, Kristina M. Yim, María F. Rosales Larios, Marybeth Baumgartner, Timothy Nottoli, James P. Noonan

**Affiliations:** 1 Department of Genetics, Yale School of Medicine, New Haven, CT 06510 USA; 2 Department of Comparative Medicine, Yale School of Medicine, New Haven, CT 06510 USA; 3 Yale Genome Editing Center, Yale School of Medicine, New Haven, CT 06510 USA; 4 Department of Ecology and Evolutionary Biology, Yale University, New Haven, CT 06520 USA; 5 Department of Neuroscience, Yale School of Medicine, New Haven, CT 06510 USA; 6 Wu Tsai Institute, Yale University, New Haven, CT 06510 USA; 7 Division of Molecular Genetics and Oncode Institute, Netherlands Cancer Institute, Amsterdam, The Netherlands; 8 Zoetis, Inc, 333 Portage St, Kalamazoo, MI 49007 USA; 9 Genome Biology and Epigenetics, Institute of Biodynamics and Biocomplexity, Department of Biology, Utrecht University, Utrecht, The Netherlands

**Author Correction: Genome Biology 25, 156 (2024)**

**https://doi.org/10.1186/s13059-024–03300-z**

Following publication of the original article [1], the authors reported an error in the description of the confidence interval shown by box-and-whisker plots in Figs. 1C, 1D, 2C, 2D, and 3E. Previously the legends for these figure panels incorrectly reported that whiskers indicate the 90% confidence interval. They have now been corrected to state that “Box plots show the interquartile range and median, and whiskers indicate the 80% confidence interval.” The same correction has been made to the legends of the following supplementary figures in Additional file 1: Fig. S4B, panels A and B of Figs. S8-11, Figs. S15-19, and Fig. S31. Corrected legends now state that “Box plots show the interquartile range and median, and whiskers indicate the 80% confidence interval.”

These errors do not change any results and conclusions of the paper.

The original article [1] has been corrected.

## Supplementary Information

Additional file 1: Supplementary figures S1-S51.

## References

[1] Kocher AA, Dutrow EV, Uebbing S, et al. Cpg island turnover events predict evolutionary changes in enhancer activity. Genome Biol. 2024;25:156. 10.1186/s13059-024-03300-z. 38872220 10.1186/s13059-024-03300-z PMC11170920

