# Supplementary material for: CpG island turnover events predict evolutionary changes in enhancer activity
Source: Genome Biol. 2024 Jun 13;25:156. doi: 10.1186/s13059-024-03300-z (PMC11170920; doi:10.1186/s13059-024-03300-z)
Supplement: Supplementary file 1 — Additional file 1: Supplementary figures S1-S51. [file 13059_2024_3300_MOESM1_ESM.docx]

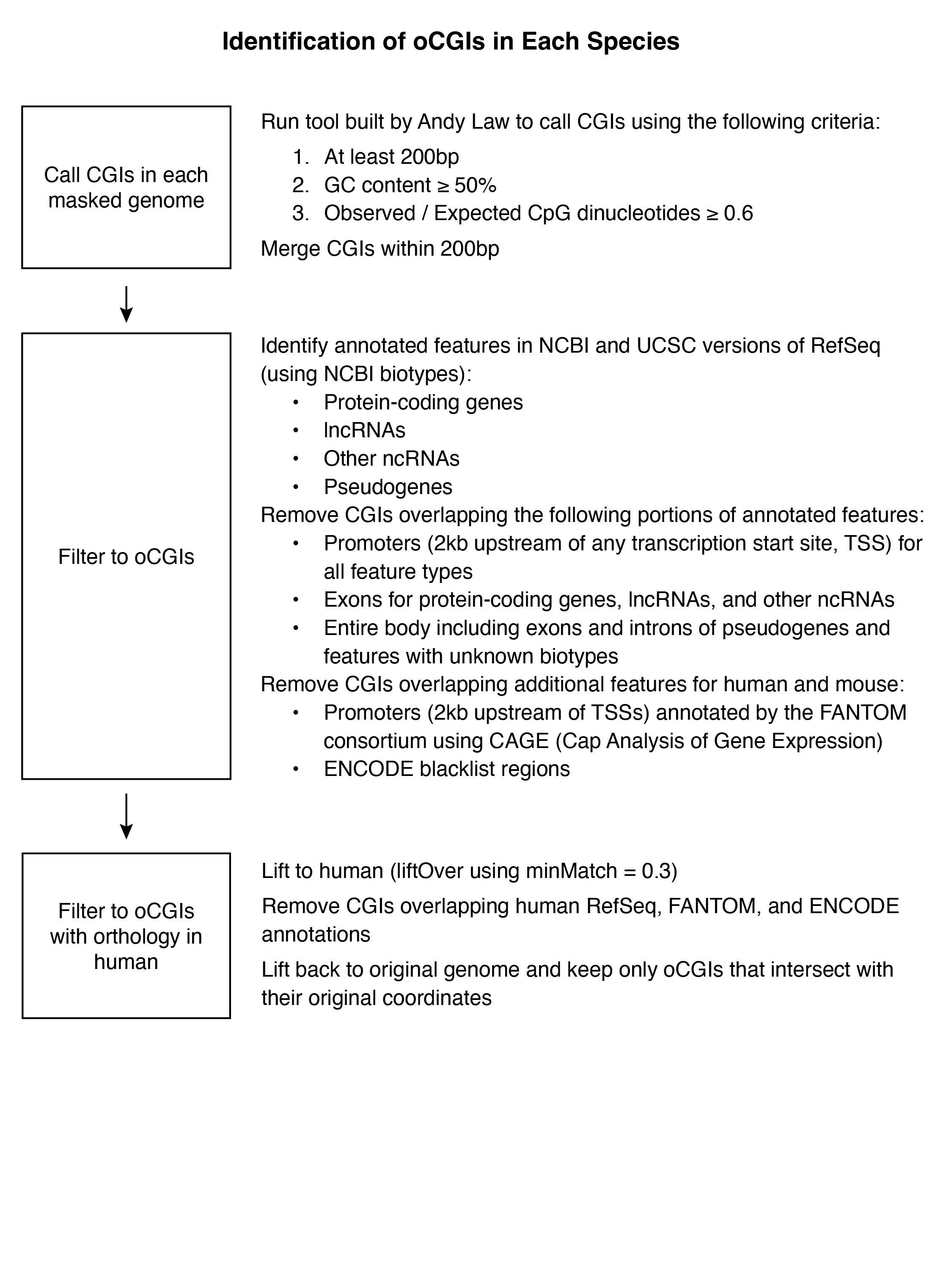


Fig S1. Pipeline for identifying oCGIs in nine mammalian species

Flow chart of the steps involved in identifying oCGIs (*left*) and details of each step (*right*). See Table S12 for a description of how NCBI biotypes were used to classify features.


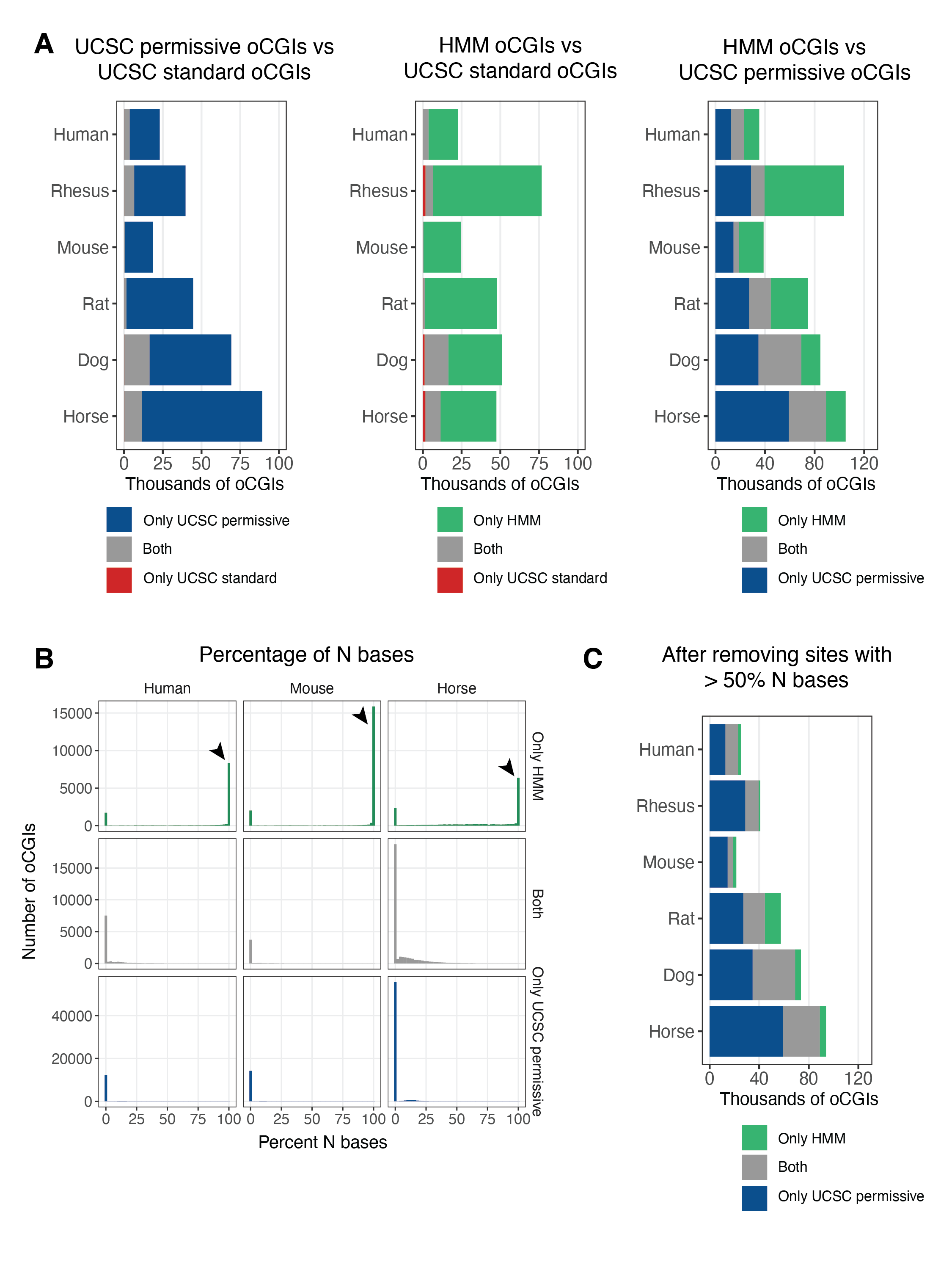


**Fig S2. Comparison of oCGIs defined by different methods**

(A) Comparison of the number of oCGIs defined using our criteria (“UCSC permissive oCGIs”) to CGIs defined using annotations from the UCSC Genome Browser (“UCSC standard oCGIs”) and CGIs defined using probabilistic models (“HMM oCGIs”) [51]. (B) Percentage of N bases (corresponding to repeat-masked sequences) in oCGIs identified only by the HMM approach, only by the UCSC permissive approach, or by both approaches. Arrowheads indicate the large number of sites identified only by the HMM approach that have close to 100% N bases in the masked genome files. (C) Comparison of HMM oCGIs and UCSC permissive oCGIs, as in rightmost plot of panel (A), but with sites containing > 50% N bases removed.


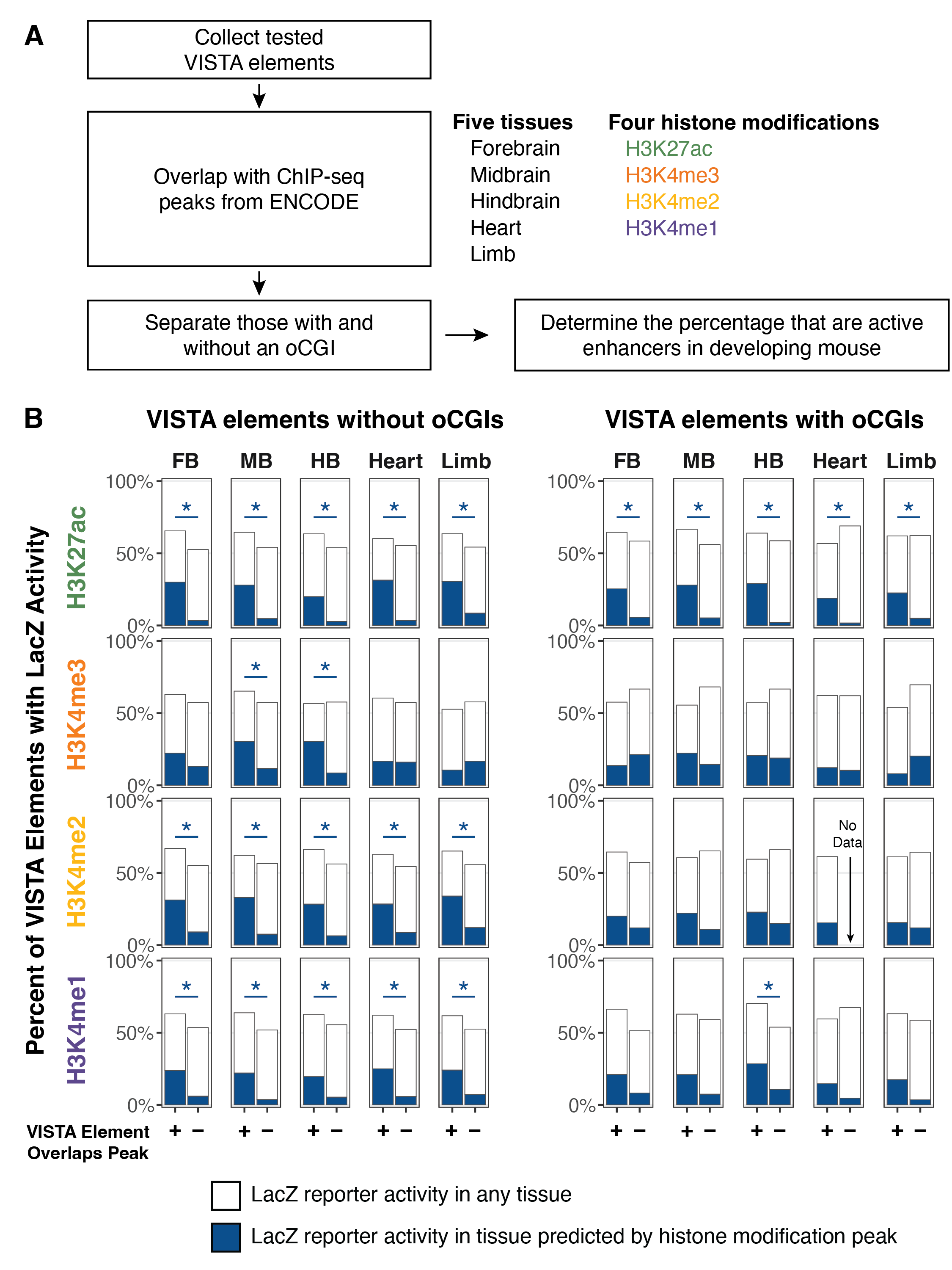


Fig S3. Histone modification peaks predict LacZ reporter assay activity

(A) Workflow for testing whether histone modification peaks are predictive of transgenic reporter activity (VISTA Enhancer Browser, [52]) using ChIP-seq data from the ENCODE consortium across five tissues in embryonic day 11.5 (E11.5) mouse. (B) Percent of tested VISTA elements that are active enhancers in the tissue predicted by ChIP-seq peaks (dark blue) or active in another tissue (white). Each panel shows a separate bar for VISTA elements that overlap a histone modification peak in the specified tissue (*left bar*) or VISTA elements that do not overlap a peak in that tissue (*right bar*). A separate analysis was performed for VISTA elements that do not overlap an oCGI (*left panel*) and VISTA elements that do overlap an oCGI (*right panel*). Stars indicate a significant association between the presence or absence of a ChIP-seq peak and the number of VISTA elements that have reporter activity in the tissue predicted by the peak (q < 0.05, Fisher’s exact test, BH-corrected).


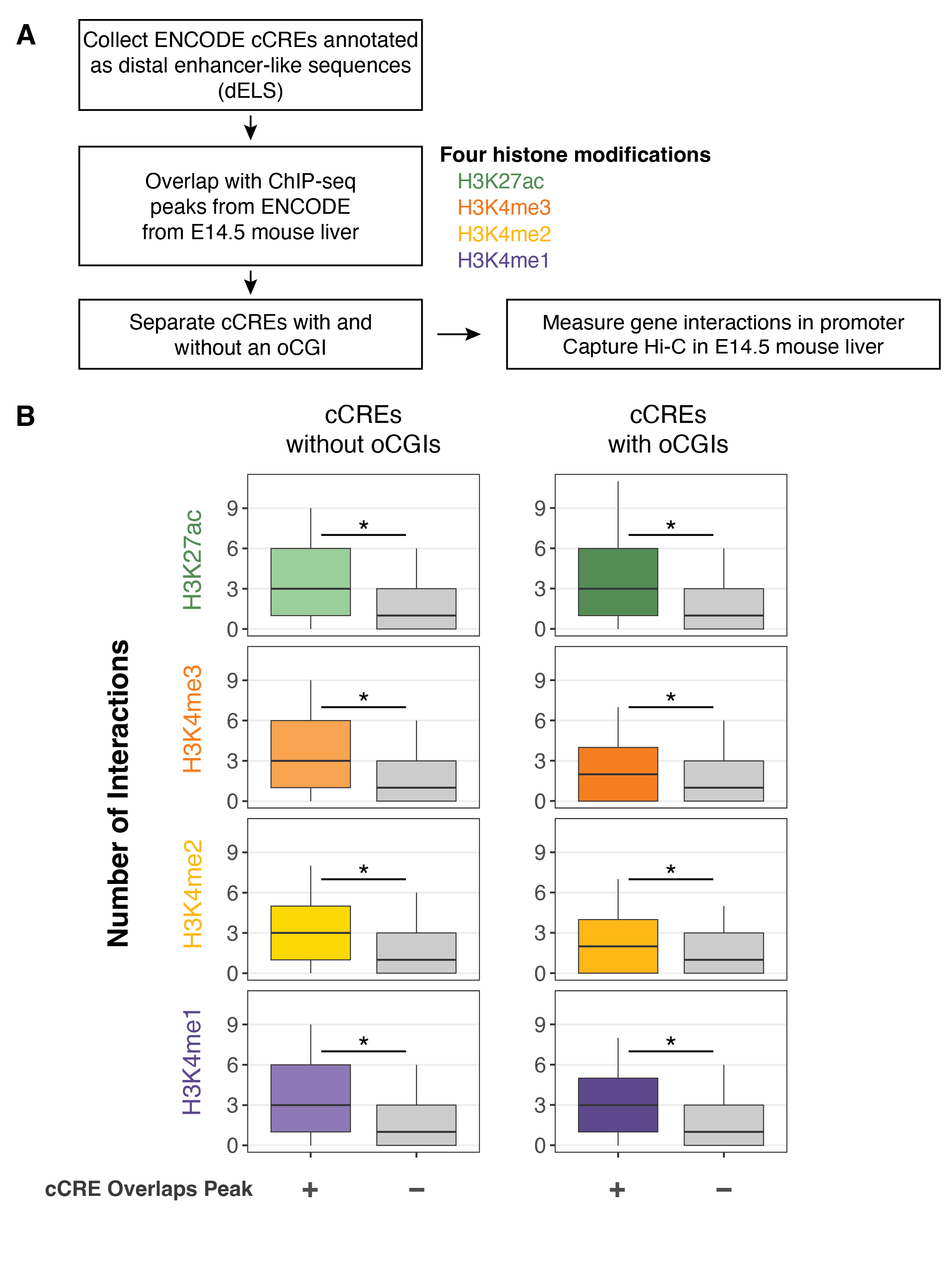


**Fig S4. Histone modification peaks at putative enhancers are associated with enhancer-promoter interactions**

(A) The workflow we used for testing whether histone modification peaks at putative enhancers are predictive of enhancer-promoter interactions using data from promoter capture HiC in E14.5 mouse liver [53]. (B) The number of enhancer-promoter interactions detected for cCREs marked by each histone modification (colored box plots) compared to unmarked cCREs (gray box plots). We performed independent analyses for cCREs that do not overlap an oCGI (*left*) and cCREs that do overlap an oCGI (*right*). Box plots show the interquartile range and median, and whiskers indicate the 80% confidence interval. Stars indicate a significant difference between cCREs that do or do not overlap a histone modification peak (q < 0.05, Wilcoxon rank-sum test, BH-corrected).


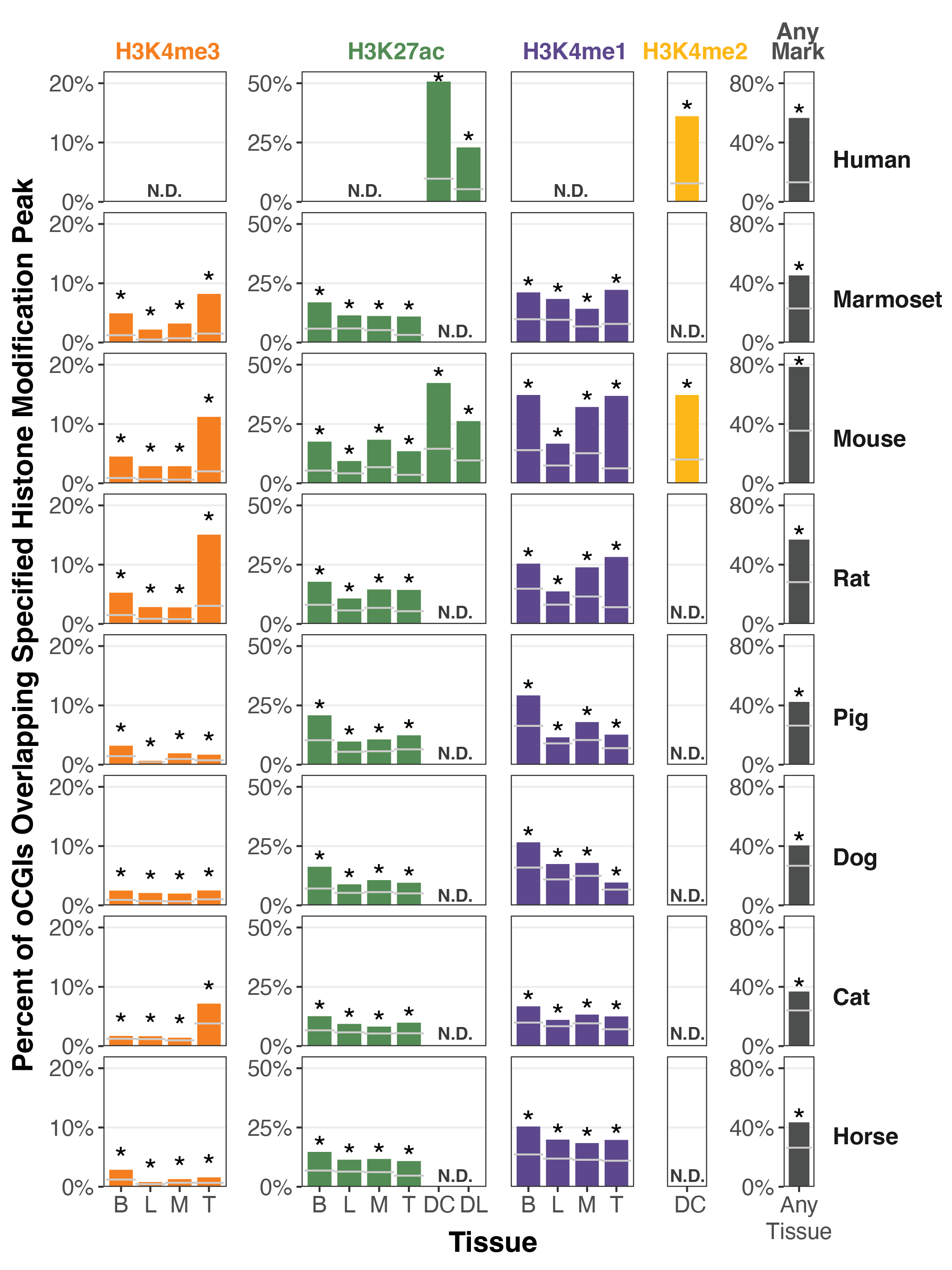


**Fig S5. oCGIs are enriched for active histone modifications in all species**

Percent of oCGIs overlapping a peak for each histone modification and in each tissue in all species except rhesus macaque (which is shown in Figure 1B). B = adult brain, L = adult liver, M = adult muscle, T = adult testis, DC = developing cortex, DL = developing limb. Gray horizontal lines indicate the expected overlap from a permutation test where oCGIs were reshuffled on the intronic and intergenic genome (see Methods). Stars indicate significance (q < 0.05 from this permutation test, BH-corrected). N.D. = no histone modification data available.


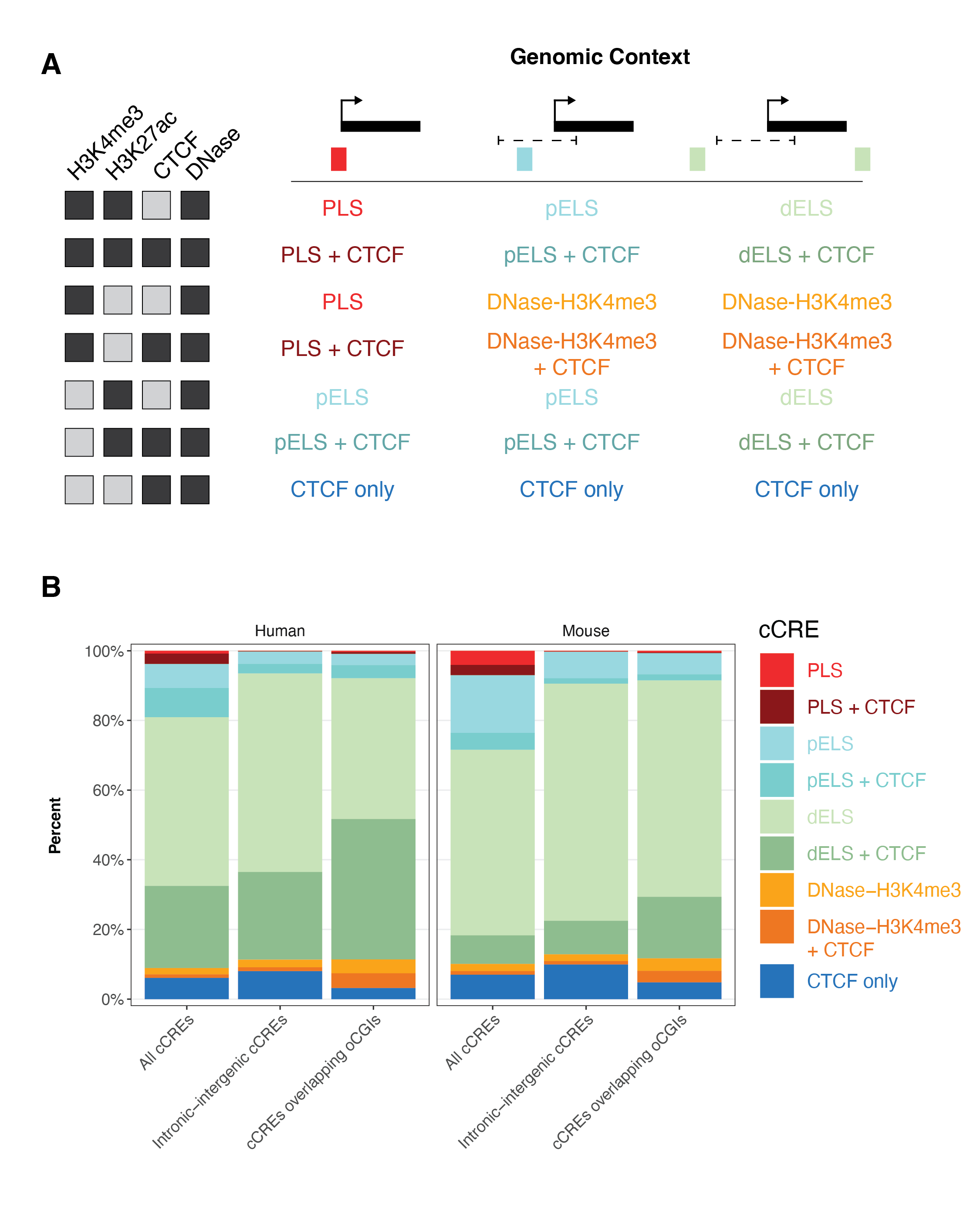


**Fig S6. Regulatory element definitions of ENCODE cCREs overlapping oCGIs**

(A) Schematic of the approach used by ENCODE to define cCREs. The approach considers genomic features such as histone modifications, CTCF binding, and DNase accessibility (*left)*. The grid indicates a set of genomic features observed in combination (black boxes indicate presence and gray boxes indicate absence). Each combination of genomic features is considered in combination with genomic context (*right)* relative to a transcription start site (TSS). The cCRE definitions for each feature and context combination are listed. PLS = promoter-like sequence, pELS = proximal enhancer-like sequence, dELS = distal enhancer-like sequence. Modeled after Figure 3 in ref. 29. (B) The percent of cCREs belonging to each definition for all cCREs, only intronic and intergenic cCREs, and cCREs overlapping oCGIs, in human (*left)* and mouse (*right)*.


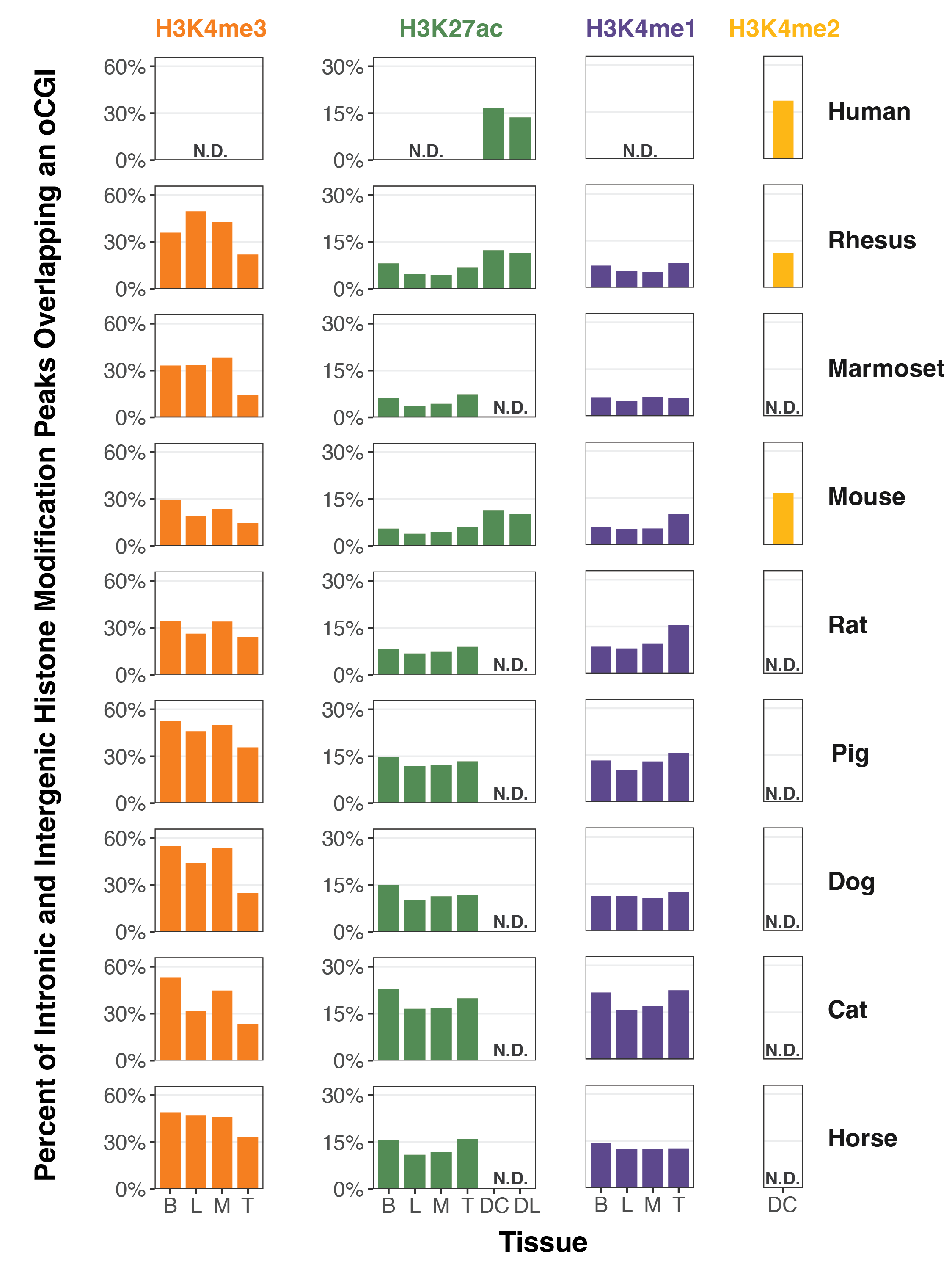


**Fig S7. Percent of histone modification peaks that overlap oCGIs**

Percent of peaks for each histone modification in each tissue and species that overlap an oCGI. B = adult brain, L = adult liver, M = adult muscle, T = adult testis, DC = developing cortex, DL = developing limb. N.D. = no histone modification data available.


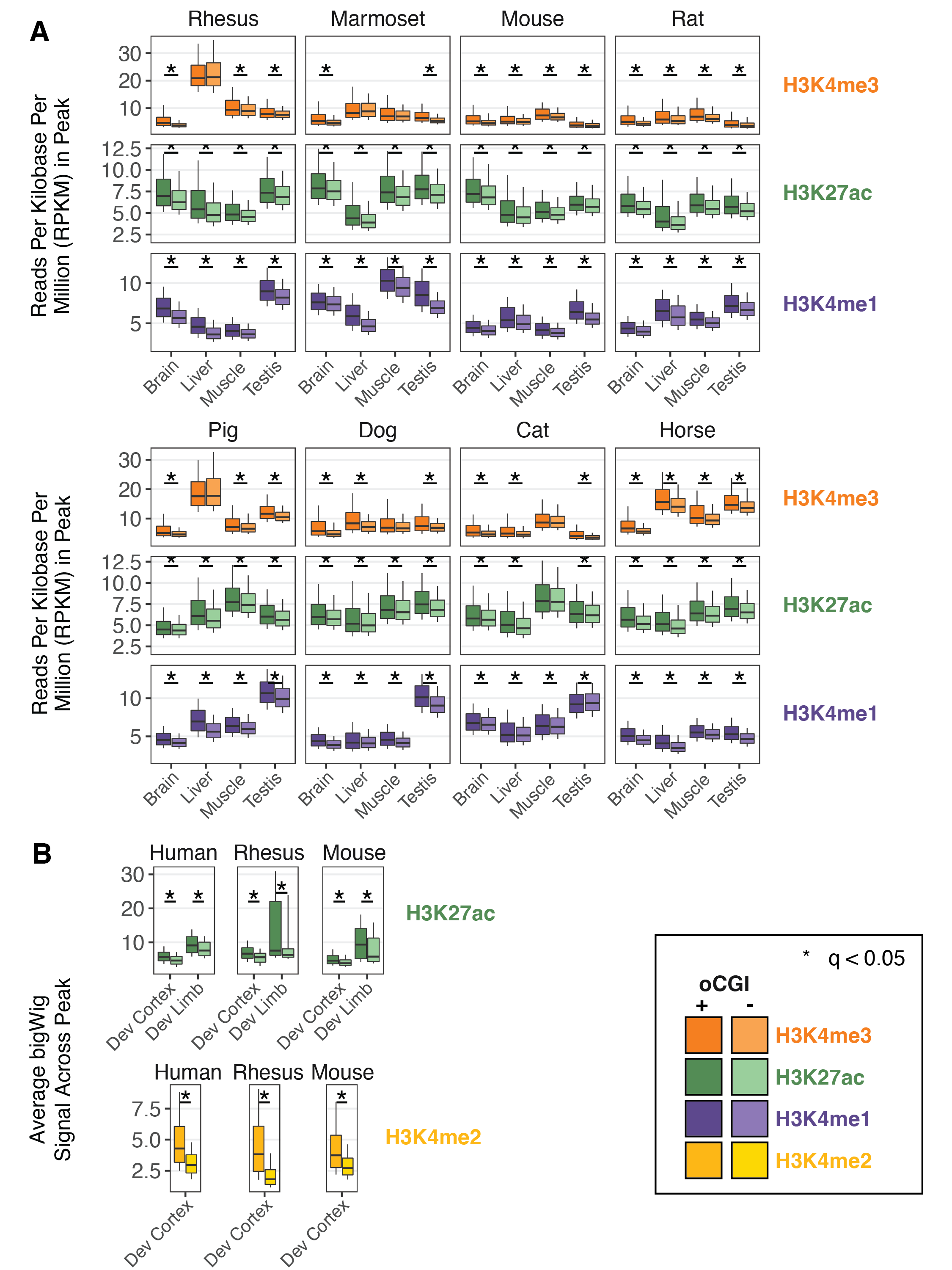


Fig S8. Histone modification peaks with oCGIs have higher histone modification levels

(A) RPKM (reads per kilobase per million mapped reads) for peaks with and without an oCGI from adult tissues. Box plots show the interquartile range and median, and whiskers indicate the 80% confidence interval. Stars indicate a significant difference between peaks with and without an oCGI (q < 0.05, Wilcoxon rank-sum test, BH-corrected). (B) Average bigWig signal (analogous to RPKM) for peaks with and without an oCGI from developing tissues. Box plots show the interquartile range and median, and whiskers indicate the 80% confidence interval. Stars indicate a significant difference between peaks with and without an oCGI (q < 0.05, Wilcoxon rank-sum test, BH-corrected).


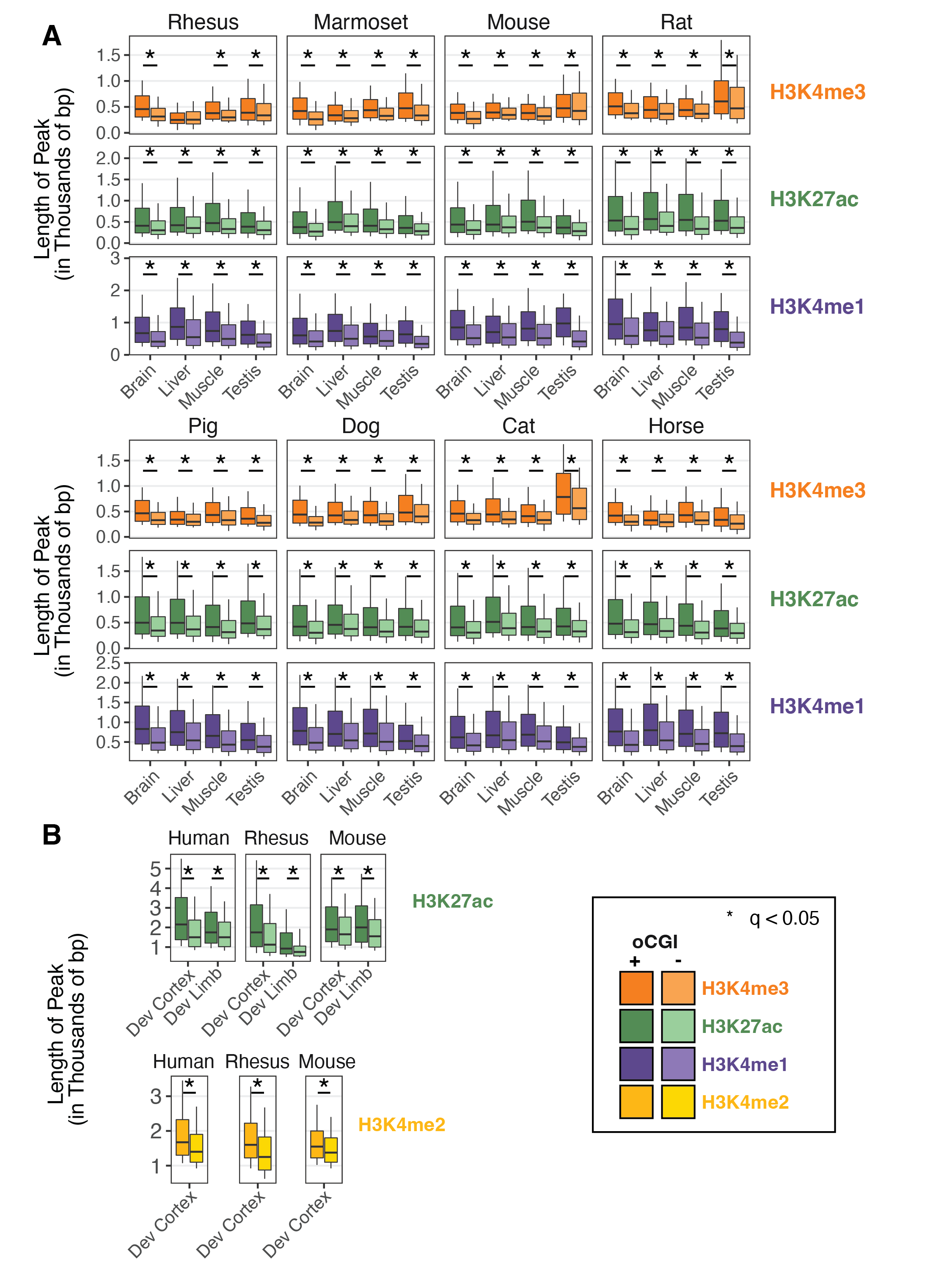


Fig S9. Histone modification peaks with oCGIs are longer

(A) Length of peaks with and without an oCGI for each histone modification in adult tissues. Box plots show the interquartile range and median, and whiskers indicate the 80% confidence interval. Stars indicate a significant difference between peaks with and without an oCGI (q < 0.05, Wilcoxon rank-sum test, BH-corrected). (B) Length of peaks with and without an oCGI for each histone modification in developing tissues. Box plots show the interquartile range and median, and whiskers indicate the 80% confidence interval. Stars indicate a significant difference between peaks with and without an oCGI (q < 0.05, Wilcoxon rank-sum test, BH-corrected).


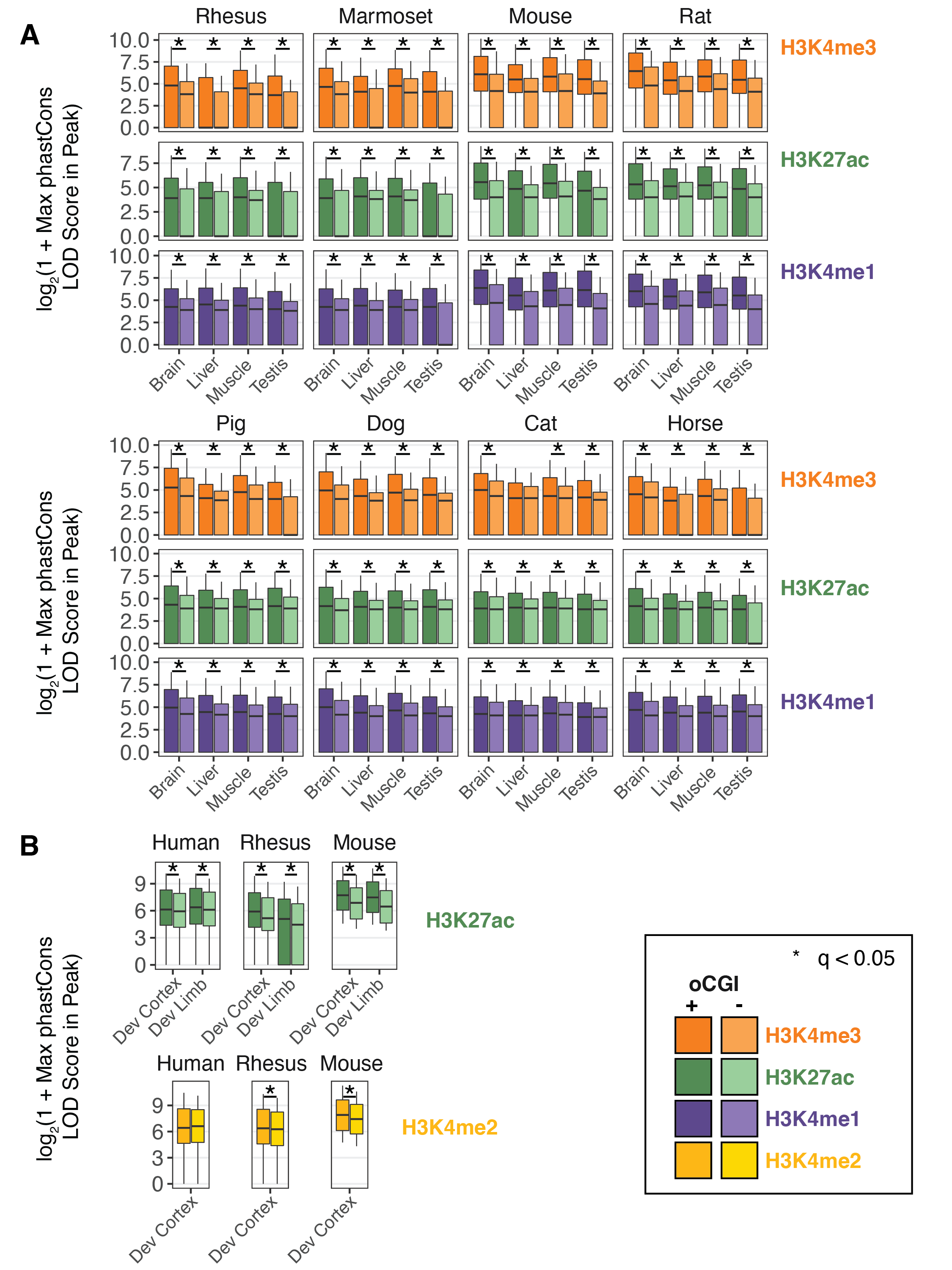


Fig S10. Histone modification peaks with oCGIs are more constrained

(A) Maximum phastCons LOD scores in peaks with and without an oCGI from adult tissues. Box plots show the interquartile range and median, and whiskers indicate the 80% confidence interval. Stars indicate a significant difference between peaks with and without an oCGI (q < 0.05, Wilcoxon rank-sum test, BH-corrected). (B) Maximum phastCons LOD scores in peaks with and without an oCGI from developing tissues. Box plots show the interquartile range and median, and whiskers indicate the 80% confidence interval. Stars indicate a significant difference between peaks with and without an oCGI (q < 0.05, Wilcoxon rank-sum test, BH-corrected).


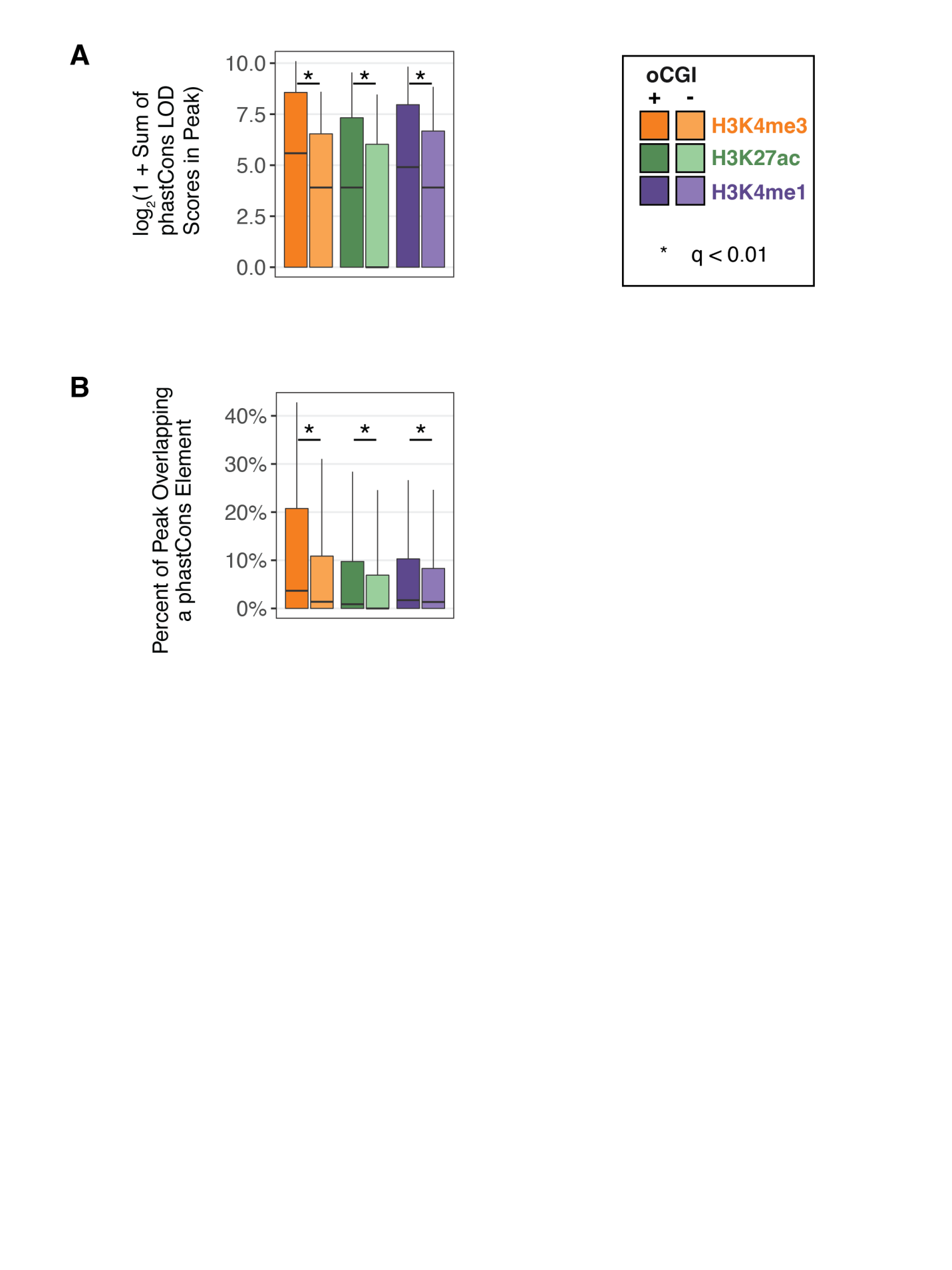


Fig S11. Alternative measures of evolutionary constraint

Results are shown for peaks with and without oCGIs for adult brain in rhesus. (A) Sum of phastCons LOD scores within histone modification peaks with and without an oCGI. Box plots show the interquartile range and median, and whiskers indicate the 80% confidence interval. Stars indicate a significant difference between peaks with and without an oCGI (q < 0.05, Wilcoxon rank-sum test, BH-corrected). (B) Percentage of bases in peak with and without an oCGI that overlap a phastCons element. Box plots show the interquartile range and median, and whiskers indicate the 80% confidence interval. Stars indicate a significant difference between peaks with and without an oCGI (q < 0.05, Wilcoxon rank-sum test, BH-corrected).


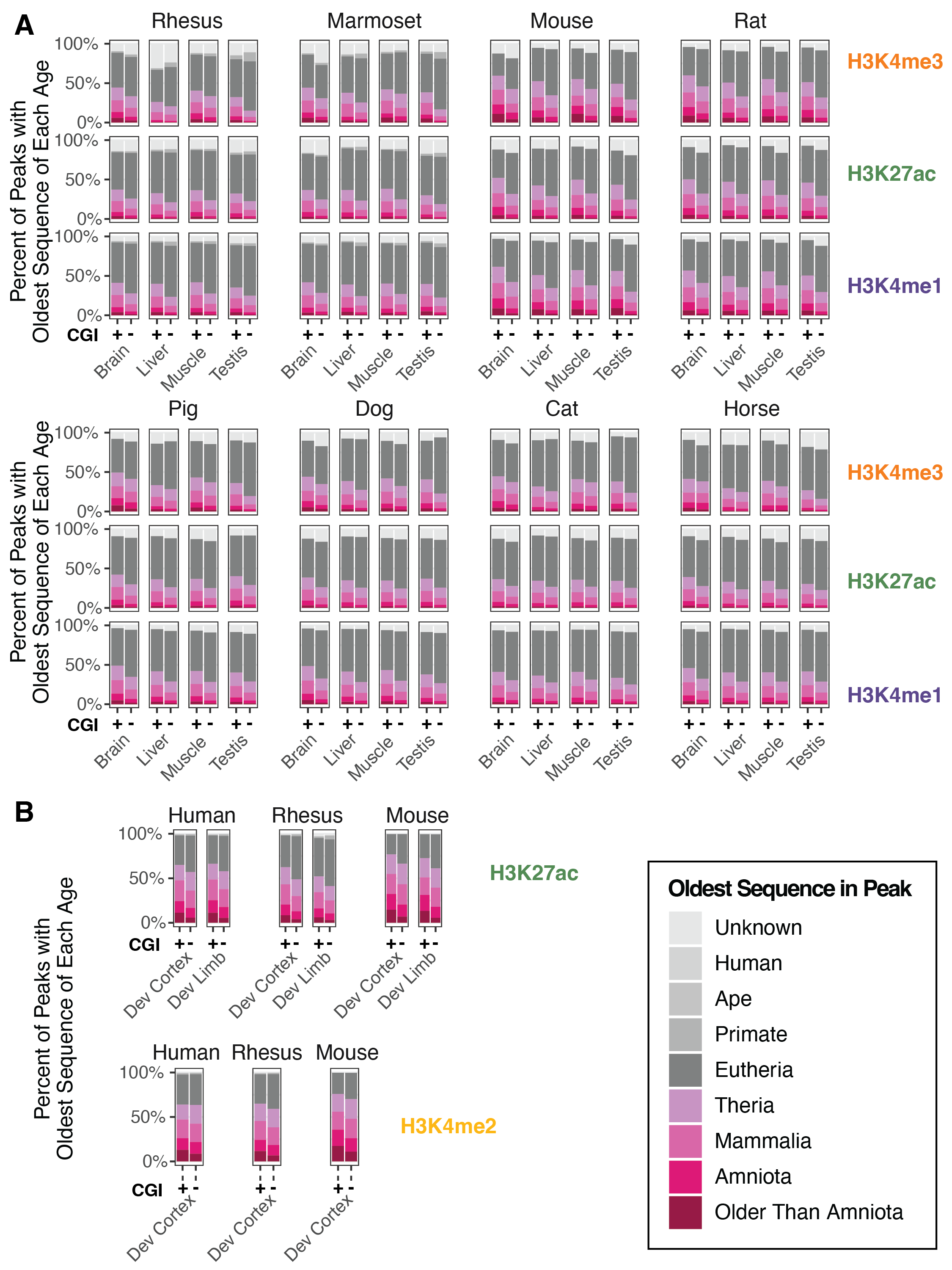


Fig S12. Histone modification peaks with oCGIs are present at older sequences

(A) The percentage of peaks with and without an oCGI from adult tissues whose oldest sequence belongs to each age category (see legend). (B) The percentage of peaks with and without an oCGI from developing tissues whose oldest sequence belongs to each age category (see legend).


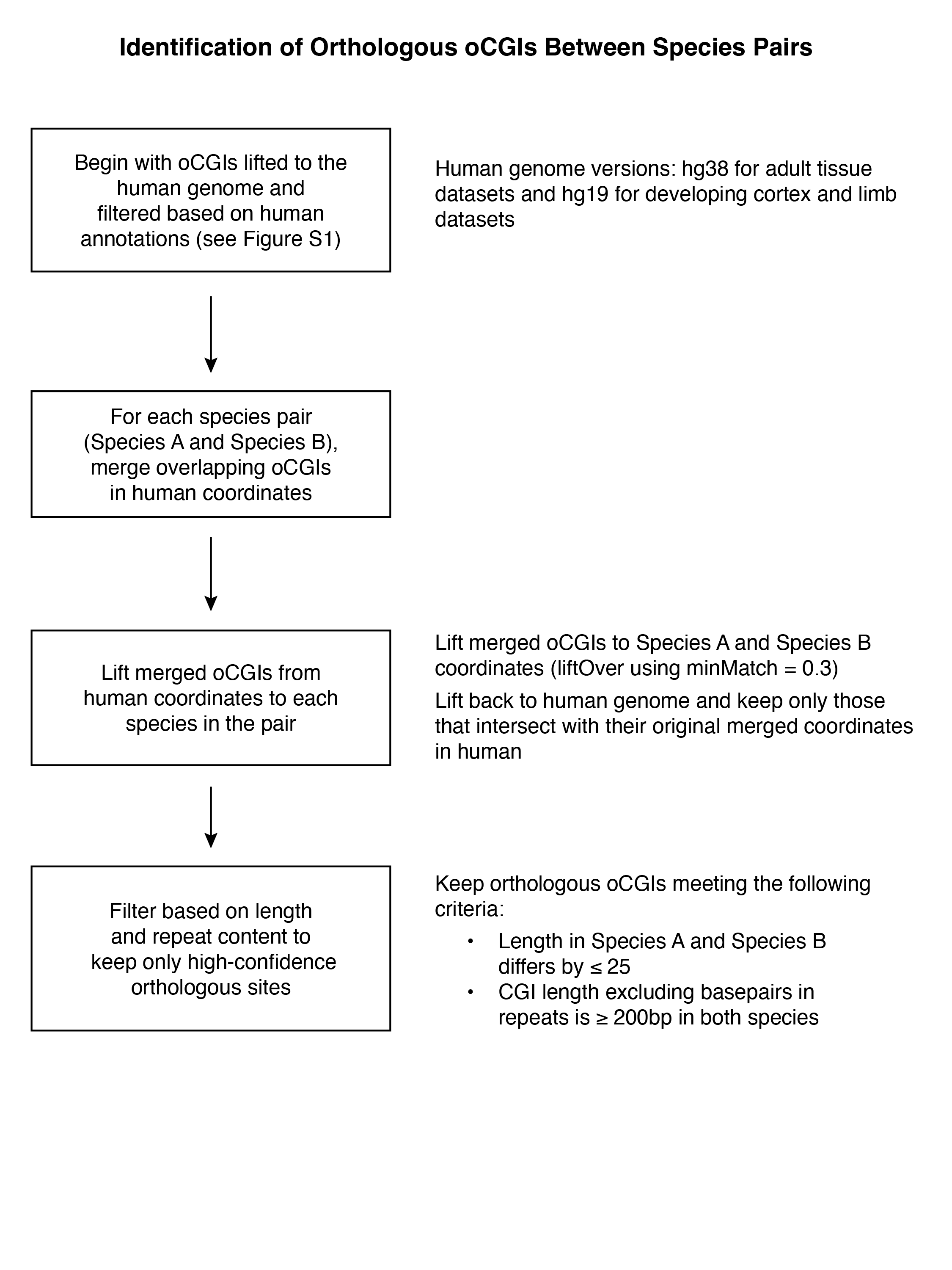


Fig S13. Pipeline for identifying orthologous oCGIs between species pairs

Flow chart of steps involved in identifying orthologous oCGIs between species pairs (*left*) and details of each step (*right*).


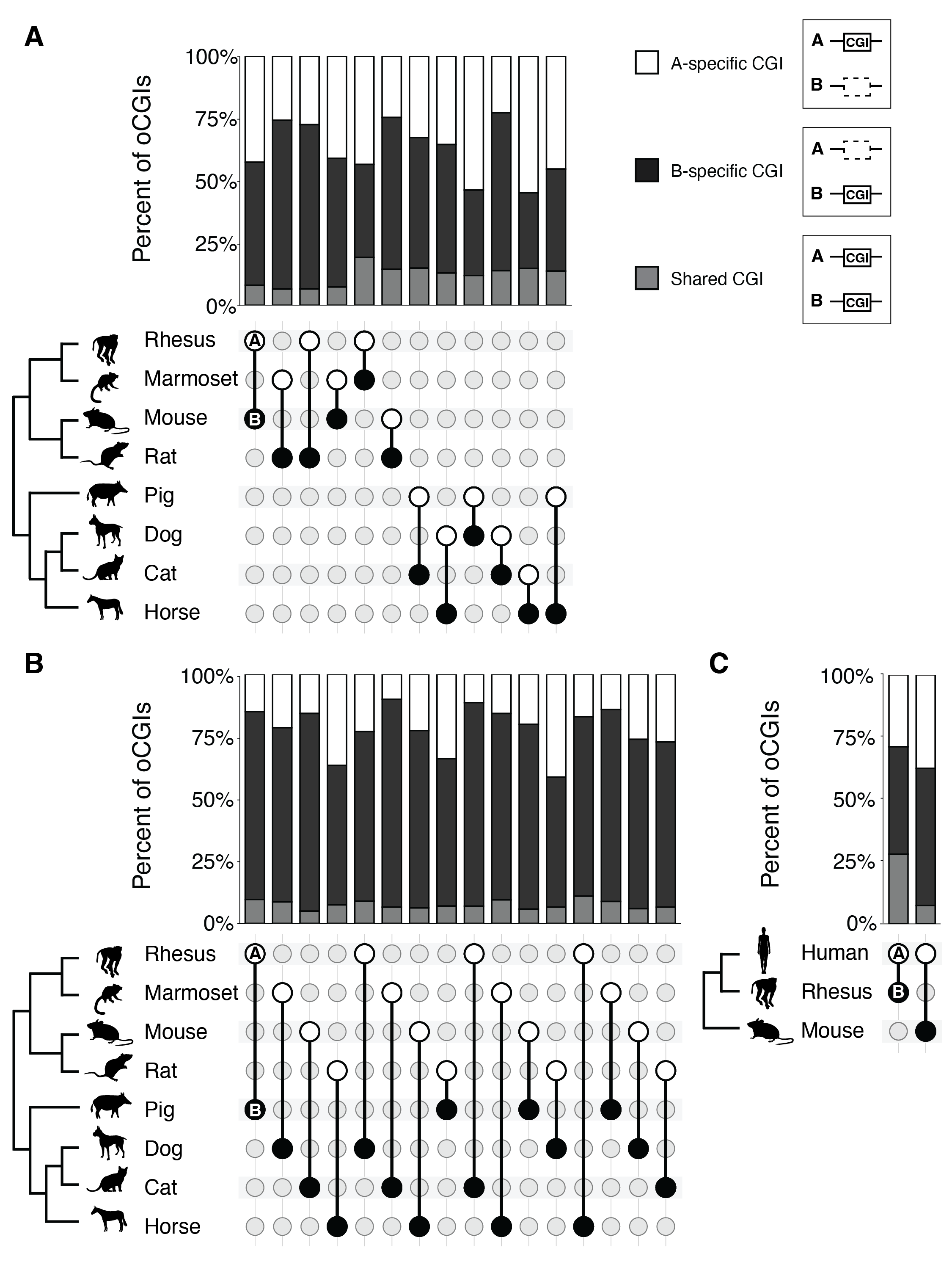


Fig S14. Divergence of oCGIs across all species pairs

Percent of oCGIs across species pairs (species A vs species B) that are A-only, B-only, or shared. In all cases species A is above species B on the phylogeny, with species A denoted by a white circle and species B denoted by a black circle. (A) More closely related species pairs. (B) More distantly related species pairs. (C) Species pairs involving human.


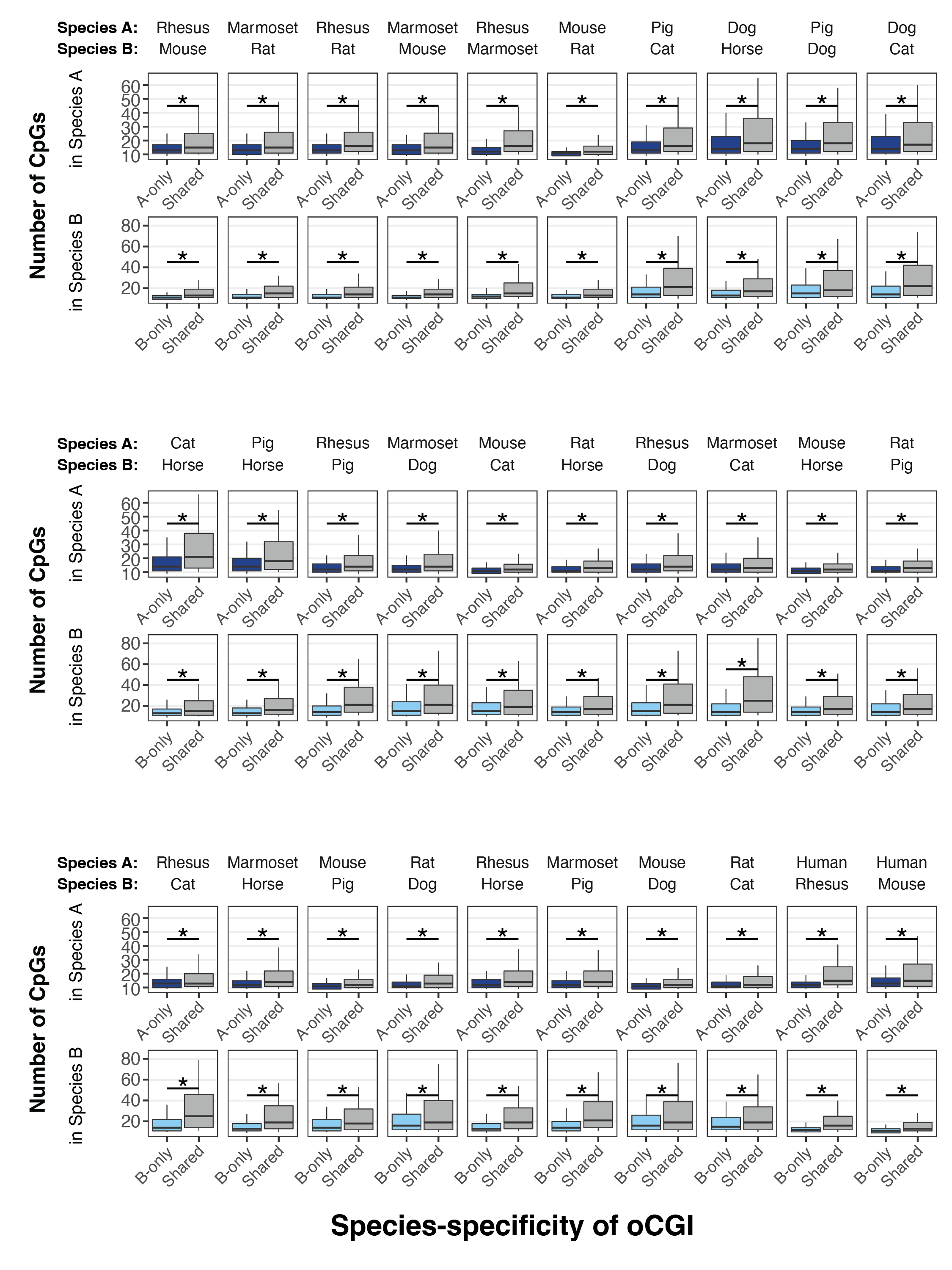


Fig S15. Shared oCGIs contain more CpG dinucleotides

Number of CpG dinucleotides in A-only (dark blue) or B-only (light blue) oCGIs compared to shared (gray) oCGIs across all species pairs considered. Box plots show the interquartile range and median, and whiskers indicate the 80% confidence interval. Stars indicate a significant difference between species-specific and shared oCGIs (q < 0.05, Wilcoxon rank-sum test, BH-corrected).


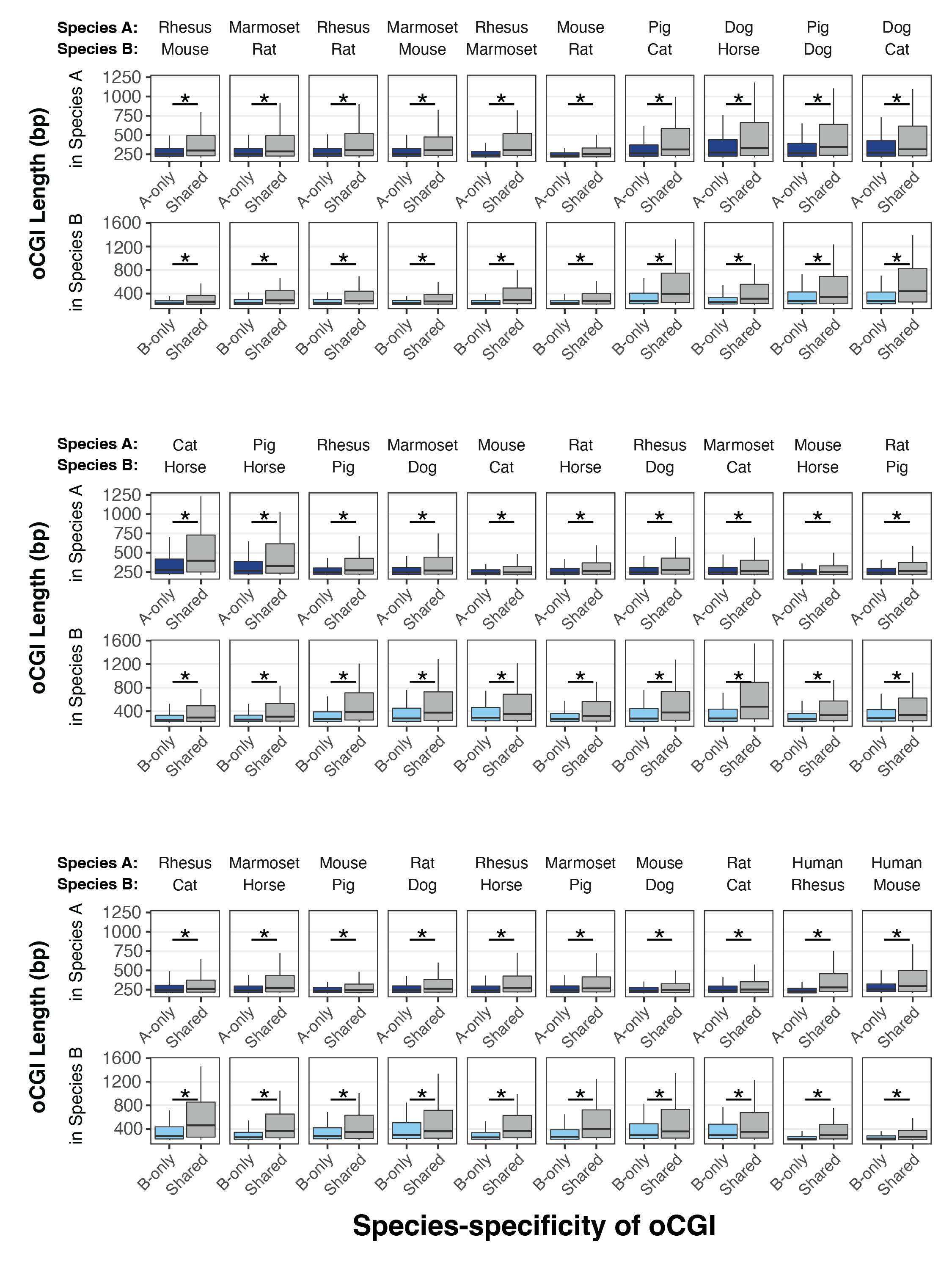


Fig S16. Shared oCGIs are longer

Length of A-only (dark blue) or B-only (light blue) oCGIs compared to shared (gray) oCGIs across all species pairs considered. Box plots show the interquartile range and median, and whiskers indicate the 80% confidence interval. Stars indicate a significant difference between species-specific and shared oCGIs (q < 0.05, Wilcoxon rank-sum test, BH-corrected).


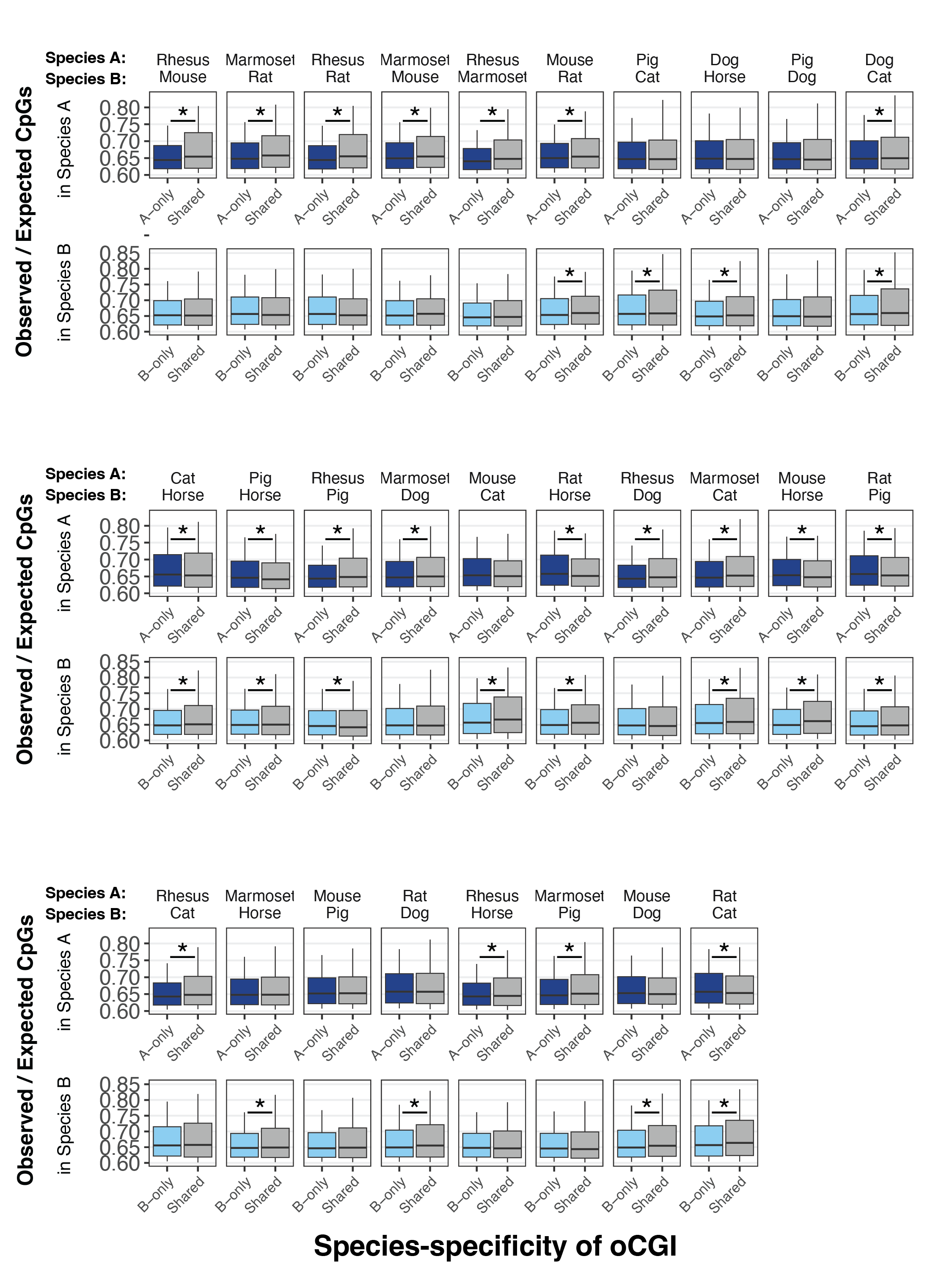


Fig S17. Shared oCGIs generally have higher observed/expected CpG dinucleotide ratios

Ratios of observed/expected CpG dinucleotides for A-only (dark blue) or B-only (light blue) oCGIs compared to shared (gray) oCGIs across all species pairs considered. Box plots show the interquartile range and median, and whiskers indicate the 80% confidence interval. Stars indicate a significant difference between species-specific and shared oCGIs (q < 0.05, Wilcoxon rank-sum test, BH-corrected).


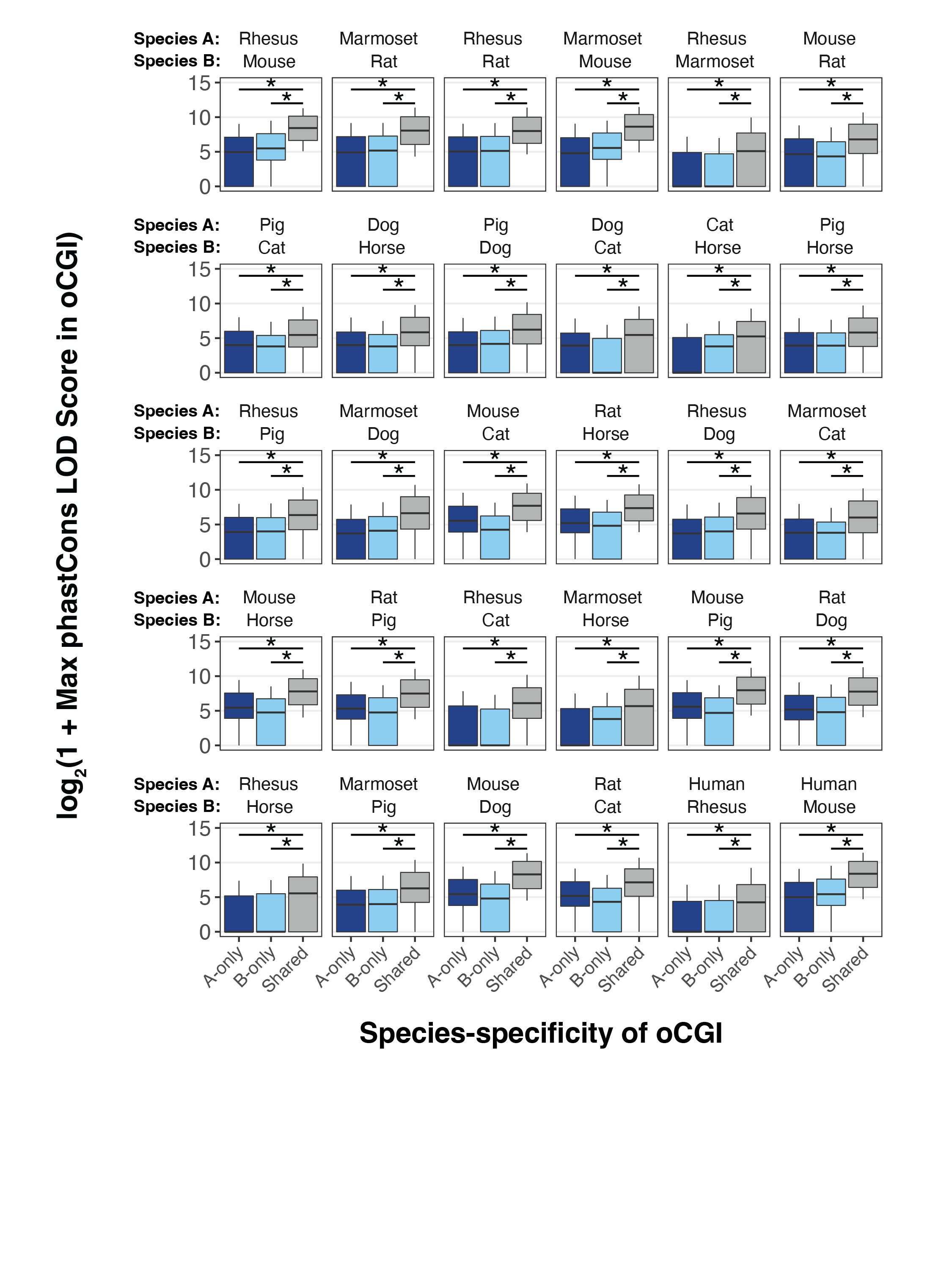


Fig S18. Shared oCGIs have higher maximum phastCons LOD scores

Maximum phastCons LOD scores in A-only (dark blue), B-only (light blue), and shared (gray) oCGIs across all species pairs considered. Box plots show the interquartile range and median, and whiskers indicate the 80% confidence interval. Stars indicate a significant difference between species-specific and shared oCGIs (q < 0.05, Wilcoxon rank-sum test, BH-corrected).


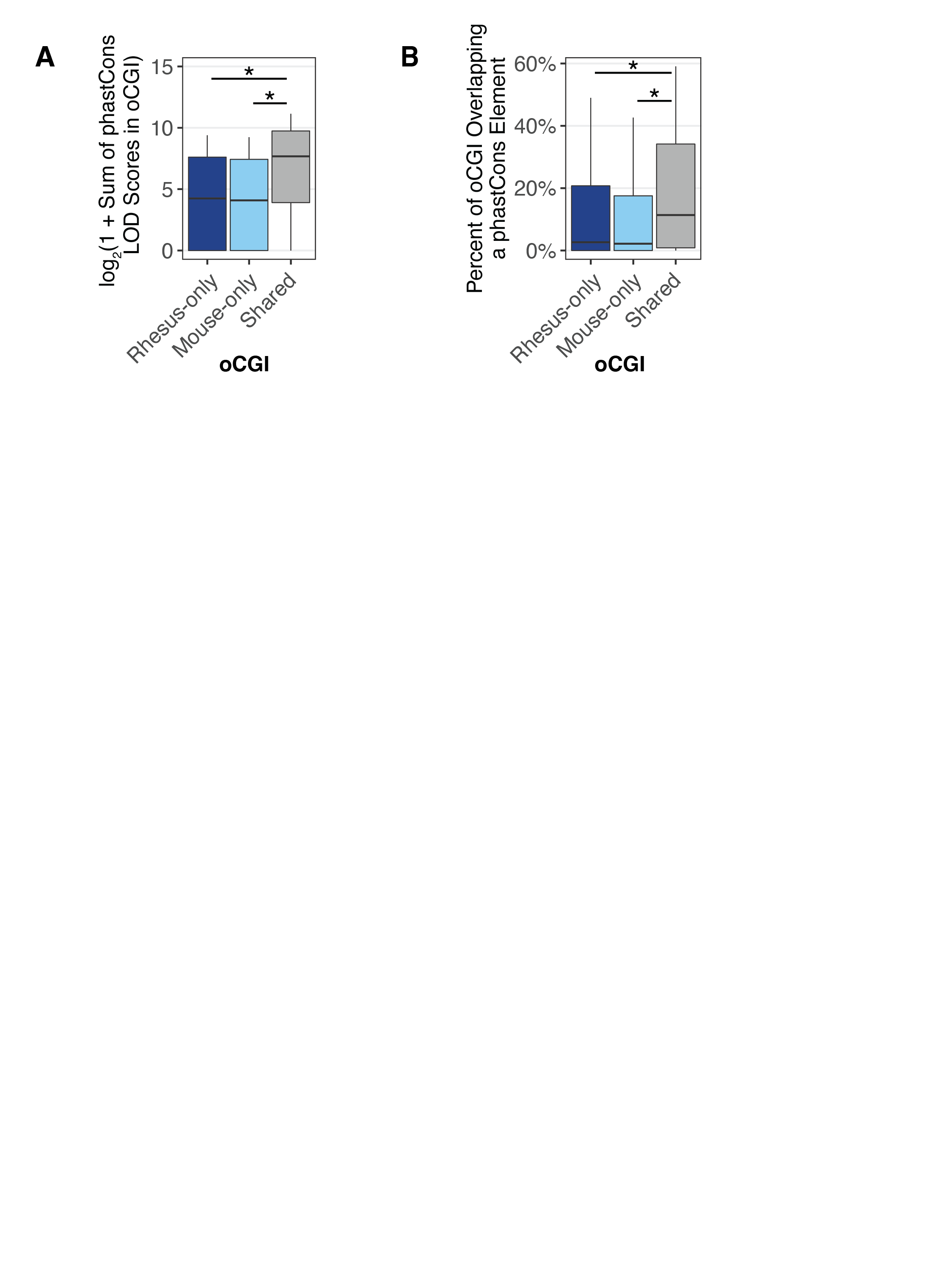


Fig S19. Alternative measures of oCGI constraint

Results are shown for the comparison between rhesus (species A) versus mouse (species B). (A) Sum of phastCons LOD scores within oCGIs that are rhesus-only, mouse-only, or shared. (B) Percent of bases that overlap a phastCons element within oCGIs that are rhesus-only, mouse-only, or shared. Box plots show the interquartile range and median, and whiskers indicate the 80% confidence interval. Stars indicate a significant difference between species-specific and shared oCGIs (q < 0.05, Wilcoxon rank-sum test, BH-corrected).


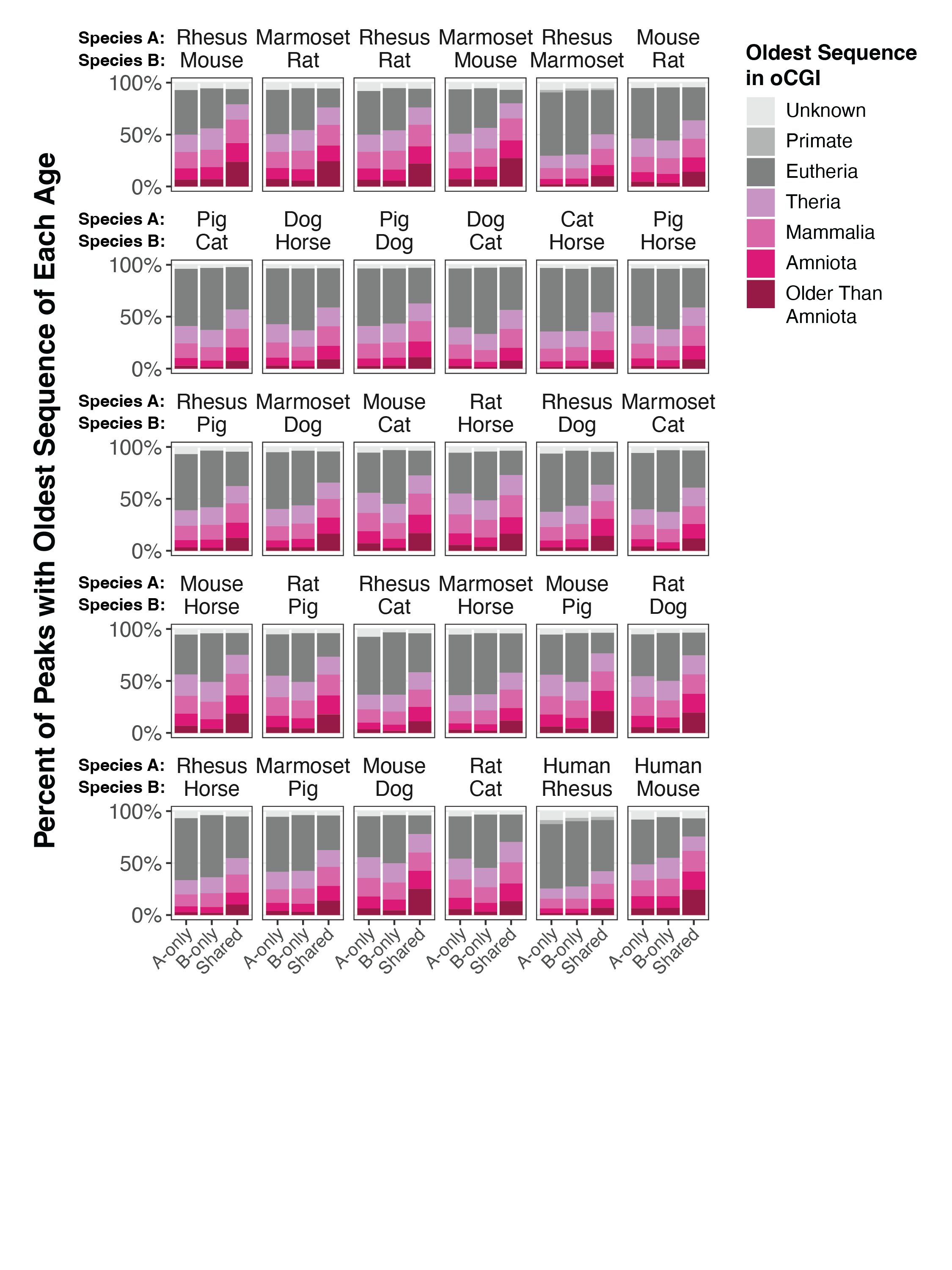


Fig S20. Shared oCGIs are present at older sequences

The percentage of A-only, B-only, and shared oCGIs whose oldest sequence belongs to each age category (see legend), across all species pairs considered.


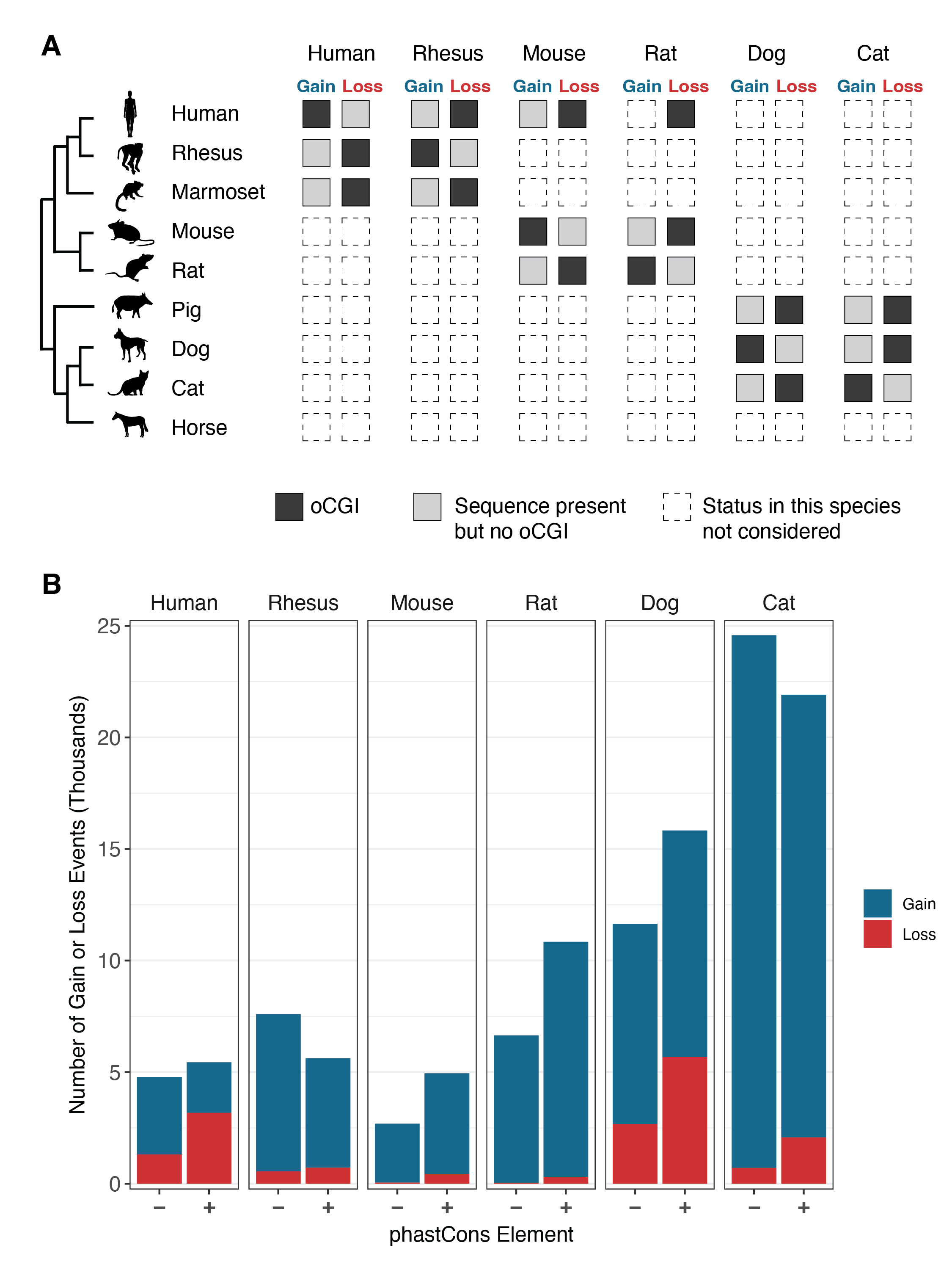


**Fig S21. Proportion of species-specific oCGI gain and oCGI loss events**

(A) Schematic of our strategy to identify gain and loss events in six species. Black boxes indicate species with an oCGI present. Gray boxes indicate species with orthologous sequence, but no oCGI. Dashed boxes indicate species in which sequence and oCGI status was not considered for identifying species-specific events. For example, status in mouse through horse was not considered for identifying human gain and loss events. (B) Number of oCGI gain and oCGI loss events identified in each species, broken down into whether they overlap a phastCons element or not as indicated in the figure. Gain events are in blue and loss events are in red.


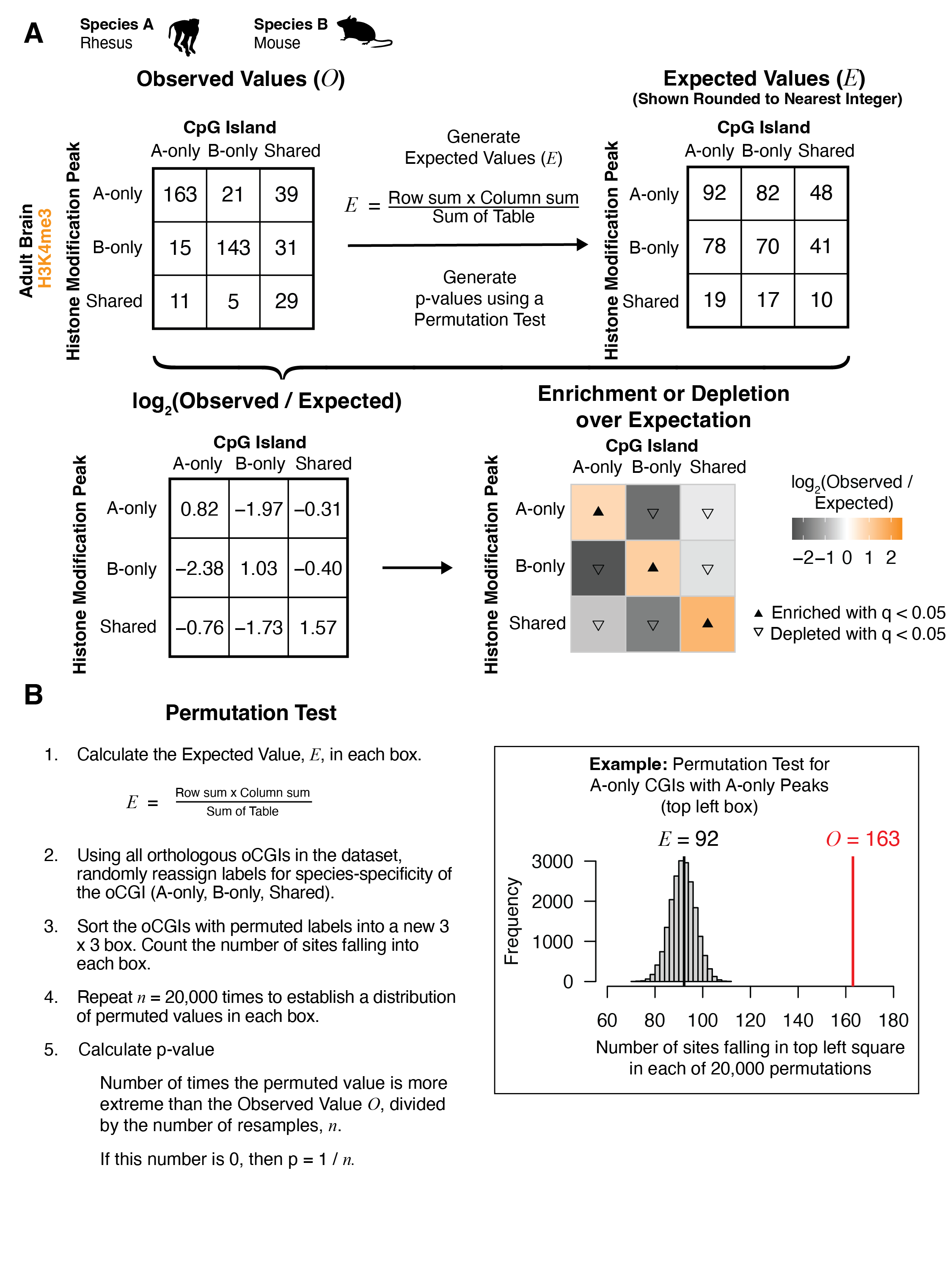


Fig S22. Permutation test used for enrichment analysis

(A) Determination of enrichment and depletion for each indicated comparison of species-specific and shared oCGIs and species-specific and shared peaks (rhesus macaque versus mouse, H3K4me3 peaks in adult brain). Observed Values and Expected Values (*top*) are used to generate log_2_-transformed ratios (*bottom left*), which are subsequently visualized as color gradients with each box colored according to the level of enrichment (orange) or depletion (gray) (*bottom right*) of genome-wide sites meeting the criteria for that box. The color bar illustrates the level of enrichment or depletion over expectation. The filled upward-pointing triangles denote significant enrichment and open downward-pointing triangles denote significant depletion (q < 0.05, permutation test, BH-corrected). (B) Explanation of permutation test to calculate statistical significance of the enrichment or depletion. The panel at right shows the distribution of values from 20,000 permutations for the top left box of the grid (A-only oCGIs with A-only peaks). The Observed Value of 163 (shown in red) is enriched compared to the Expected Value of 92, which is the mean of the 20,000 permuted values.


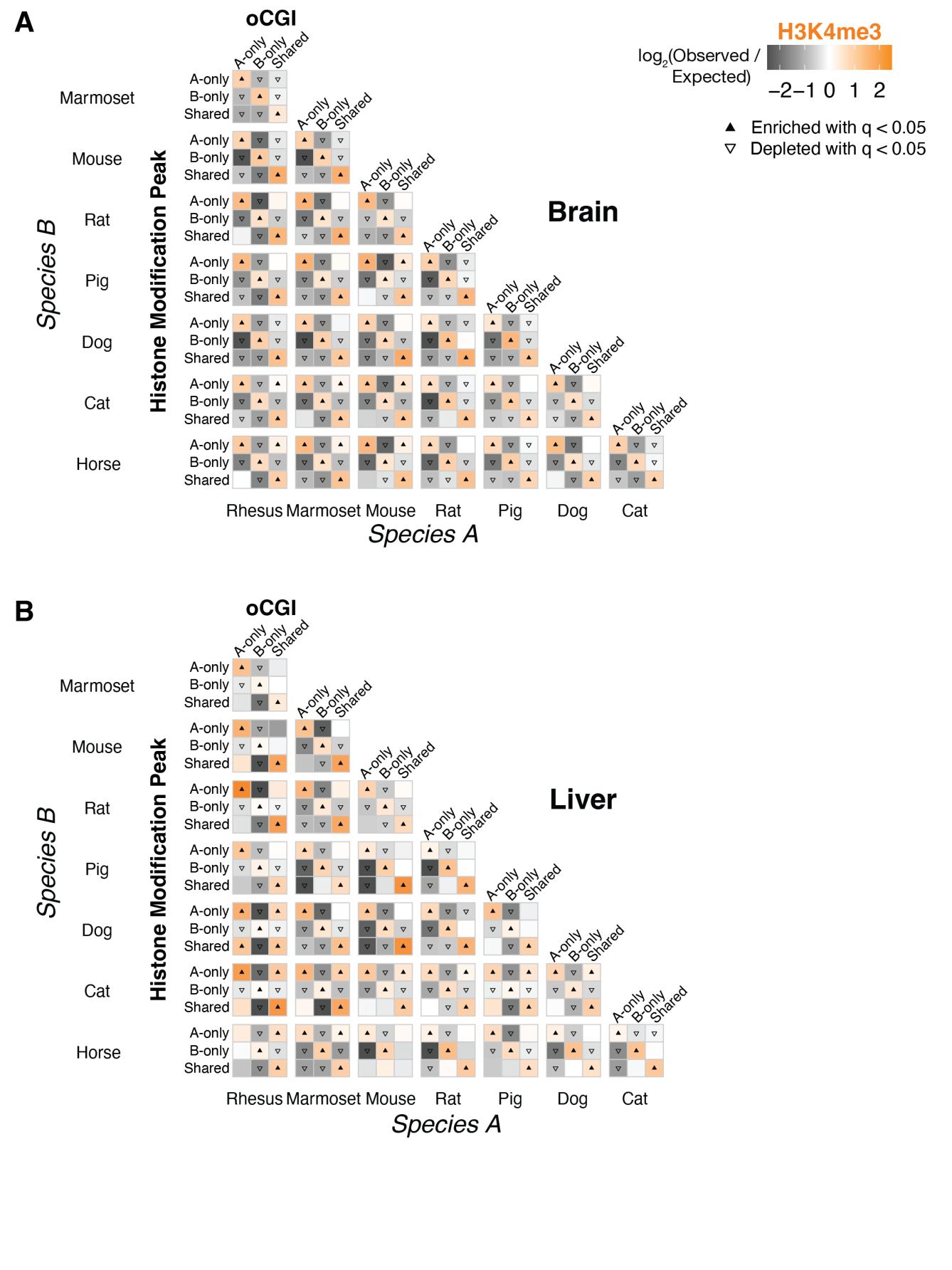


Fig S23. Species with oCGI predicts species with H3K4me3 peak (brain and liver)

Enrichment and depletion in each indicated comparison of species-specific and shared oCGIs (*top:* A-only, B-only, Shared) and species-specific and shared peaks (*left*: A-only, B-only, Shared), compared to a null expectation of no association between oCGI turnover and peak turnover. Each 3 x 3 grid shows the results for a specific test examining oCGIs and their overlap with H3K4me3 in adult brain (A) or liver (B) within a given species pair. Each box in each grid is colored according to the level of enrichment over expectation (orange) or depletion (gray) of genome-wide sites that meet the criteria for that box. The color bar illustrates the level of enrichment or depletion over expectation. For visualization, values greater than 2.5 and less than -2.5 were set to 2.5 and -2.5, respectively. The filled upward-pointing triangles denote significant enrichment and open downward-pointing triangles denote significant depletion (q < 0.05, permutation test, BH-corrected, see Fig. S22 and Methods).


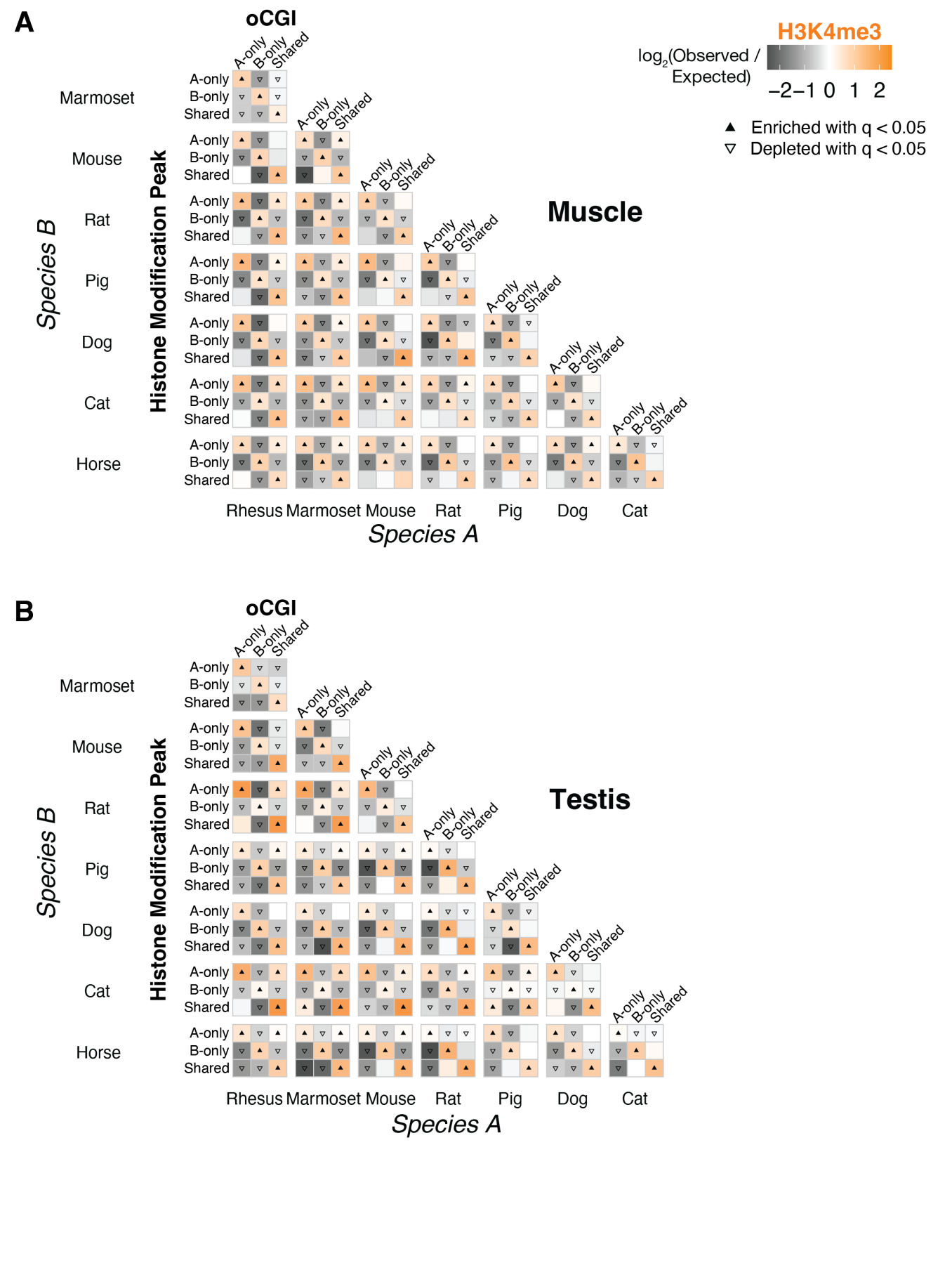


Fig S24. Species with oCGI predicts species with H3K4me3 peak (muscle and testis)

Enrichment and depletion in each indicated comparison of species-specific and shared oCGIs (*top:* A-only, B-only, Shared) and species-specific and shared peaks (*left*: A-only, B-only, Shared), compared to a null expectation of no association between oCGI turnover and peak turnover. Each 3 x 3 grid shows the results for a specific test examining oCGIs and their overlap with H3K4me3 in adult muscle (A) or testis (B) within a given species pair. Each box in each grid is colored according to the level of enrichment over expectation (orange) or depletion (gray) of genome-wide sites that meet the criteria for that box. The color bar illustrates the level of enrichment or depletion over expectation. For visualization, values greater than 2.5 and less than -2.5 were set to 2.5 and -2.5, respectively. The filled upward-pointing triangles denote significant enrichment and open downward-pointing triangles denote significant depletion (q < 0.05, permutation test, BH-corrected, see Fig. S22 and Methods).


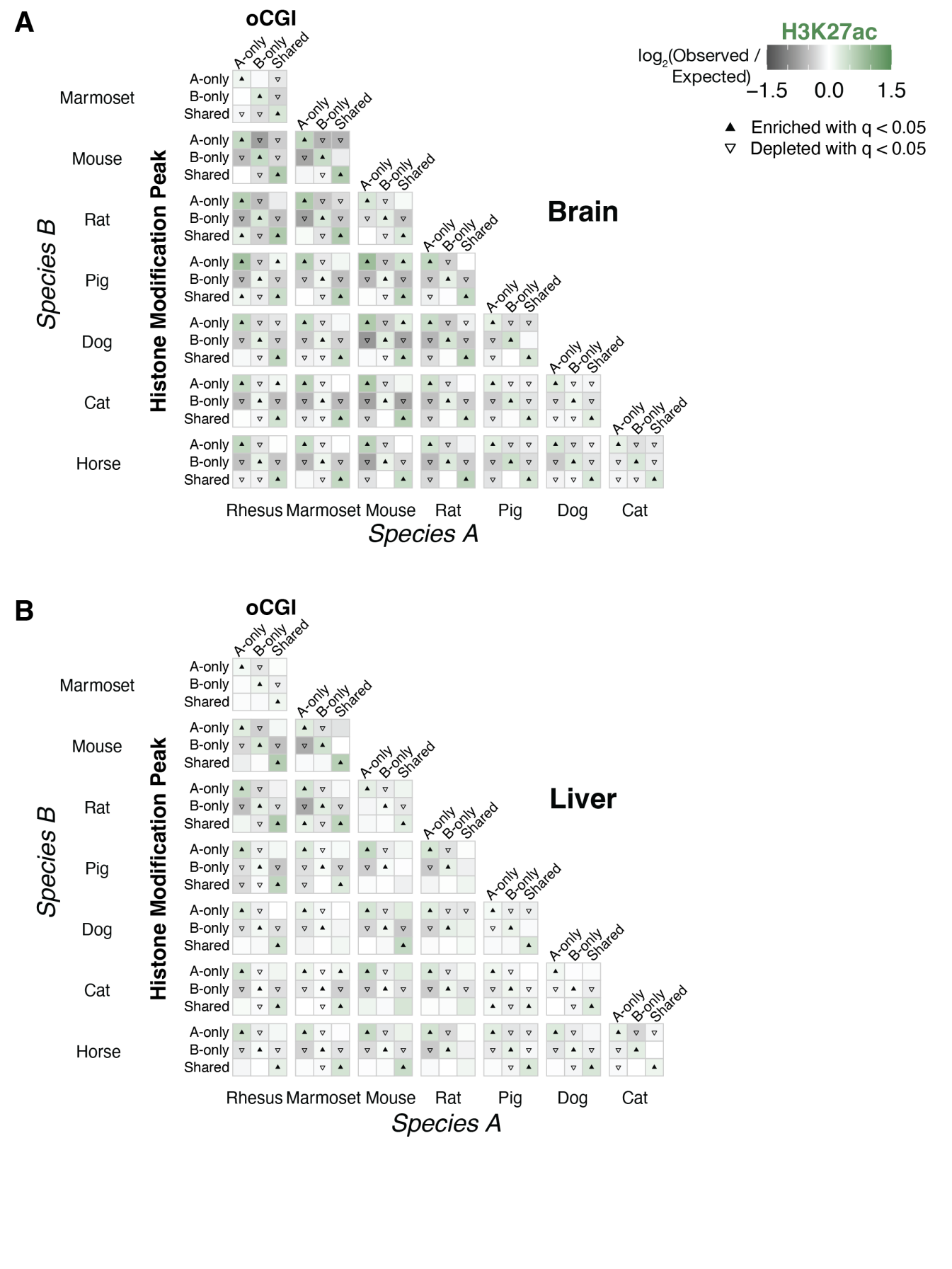


Fig S25. Species with oCGI predicts species with H3K27ac peak (brain and liver)

Enrichment and depletion in each indicated comparison of species-specific and shared oCGIs (*top:* A-only, B-only, Shared) and species-specific and shared peaks (*left*: A-only, B-only, Shared), compared to a null expectation of no association between oCGI turnover and peak turnover. Each 3 x 3 grid shows the results for a specific test examining oCGIs and their overlap with H3K27ac in adult brain (A) or liver (B) within a given species pair. Each box in each grid is colored according to the level of enrichment over expectation (green) or depletion (gray) of genome-wide sites that meet the criteria for that box. The color bar illustrates the level of enrichment or depletion over expectation. For visualization, values greater than 1.5 and less than -1.5 were set to 1.5 and -1.5, respectively. The filled upward-pointing triangles denote significant enrichment and open downward-pointing triangles denote significant depletion (q < 0.05, permutation test, BH-corrected, see Fig. S22 and Methods).


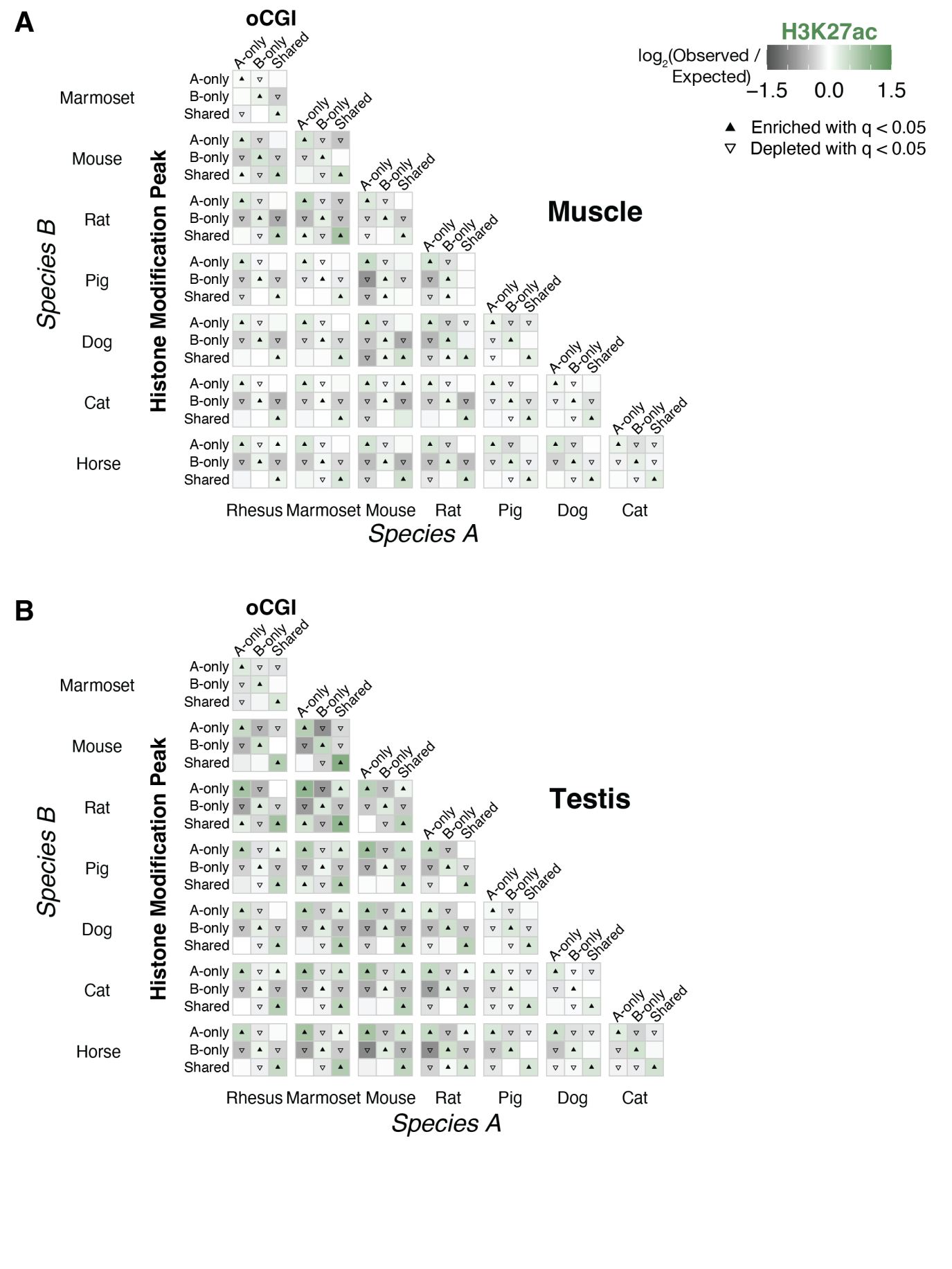


Fig S26. Species with oCGI predicts species with H3K27ac peak (muscle and testis)

Enrichment and depletion in each indicated comparison of species-specific and shared oCGIs (*top:* A-only, B-only, Shared) and species-specific and shared peaks (*left*: A-only, B-only, Shared), compared to a null expectation of no association between oCGI turnover and peak turnover. Each 3 x 3 grid shows the results for a specific test examining oCGIs and their overlap with H3K27ac in adult muscle (A) or testis (B) within a given species pair. Each box in each grid is colored according to the level of enrichment over expectation (green) or depletion (gray) of genome-wide sites that meet the criteria for that box. The color bar illustrates the level of enrichment or depletion over expectation. For visualization, values greater than 1.5 and less than -1.5 were set to 1.5 and -1.5, respectively. The filled upward-pointing triangles denote significant enrichment and open downward-pointing triangles denote significant depletion (q < 0.05, permutation test, BH-corrected, see Fig. S22 and Methods).


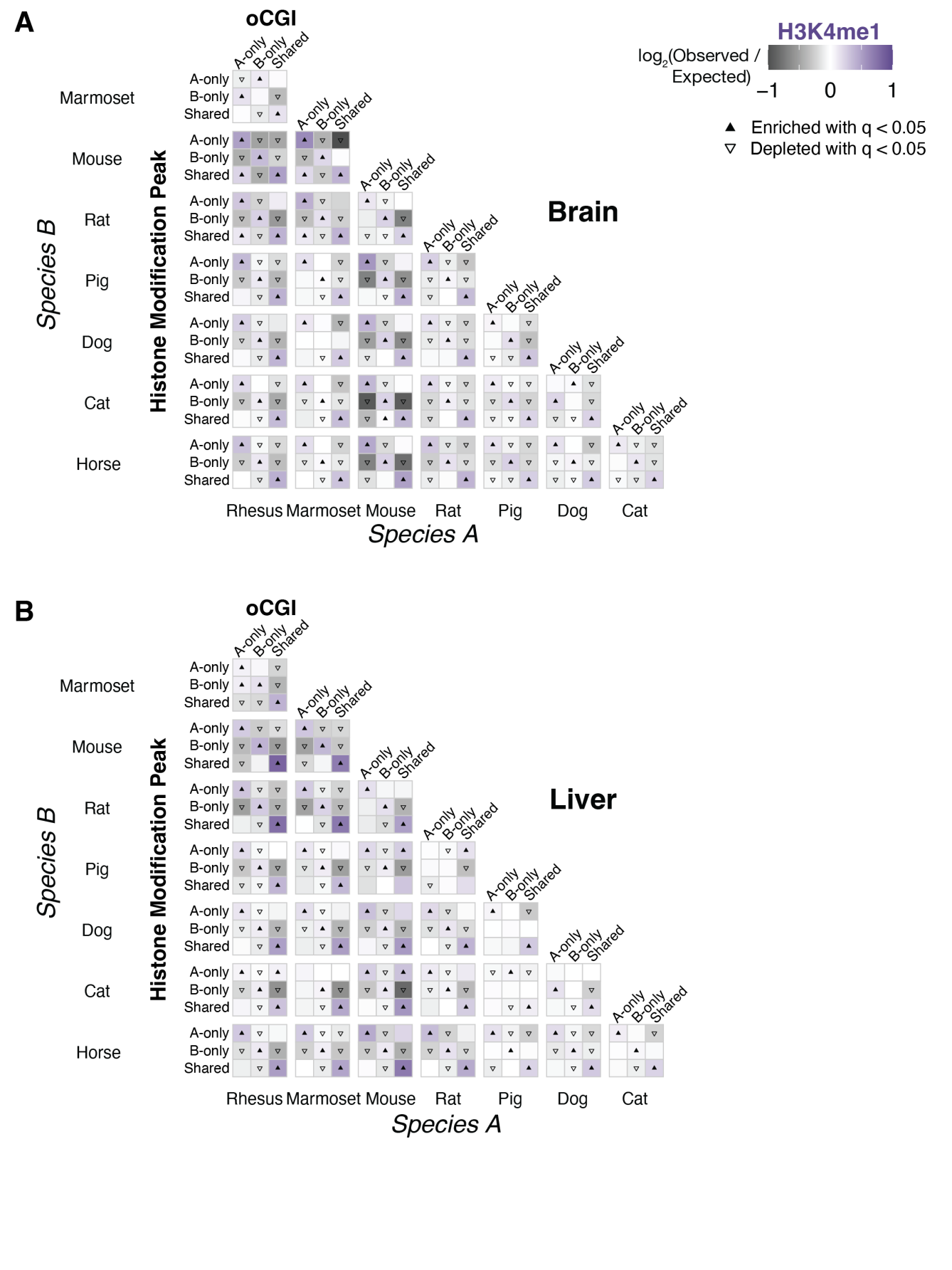


Fig S27. Species with oCGI predicts species with H3K4me1 peak (brain and liver)

Enrichment and depletion in each indicated comparison of species-specific and shared oCGIs (*top:* A-only, B-only, Shared) and species-specific and shared peaks (*left*: A-only, B-only, Shared), compared to a null expectation of no association between oCGI turnover and peak turnover. Each 3 x 3 grid shows the results for a specific test examining oCGIs and their overlap with H3K4me1 in adult brain (A) or liver (B) within a given species pair. Each box in each grid is colored according to the level of enrichment over expectation (purple) or depletion (gray) of genome-wide sites that meet the criteria for that box. The color bar illustrates the level of enrichment or depletion over expectation. For visualization, values greater than 1 and less than -1 were set to 1 and -1, respectively. The filled upward-pointing triangles denote significant enrichment and open downward-pointing triangles denote significant depletion (q < 0.05, permutation test, BH-corrected, see Fig. S22 and Methods).


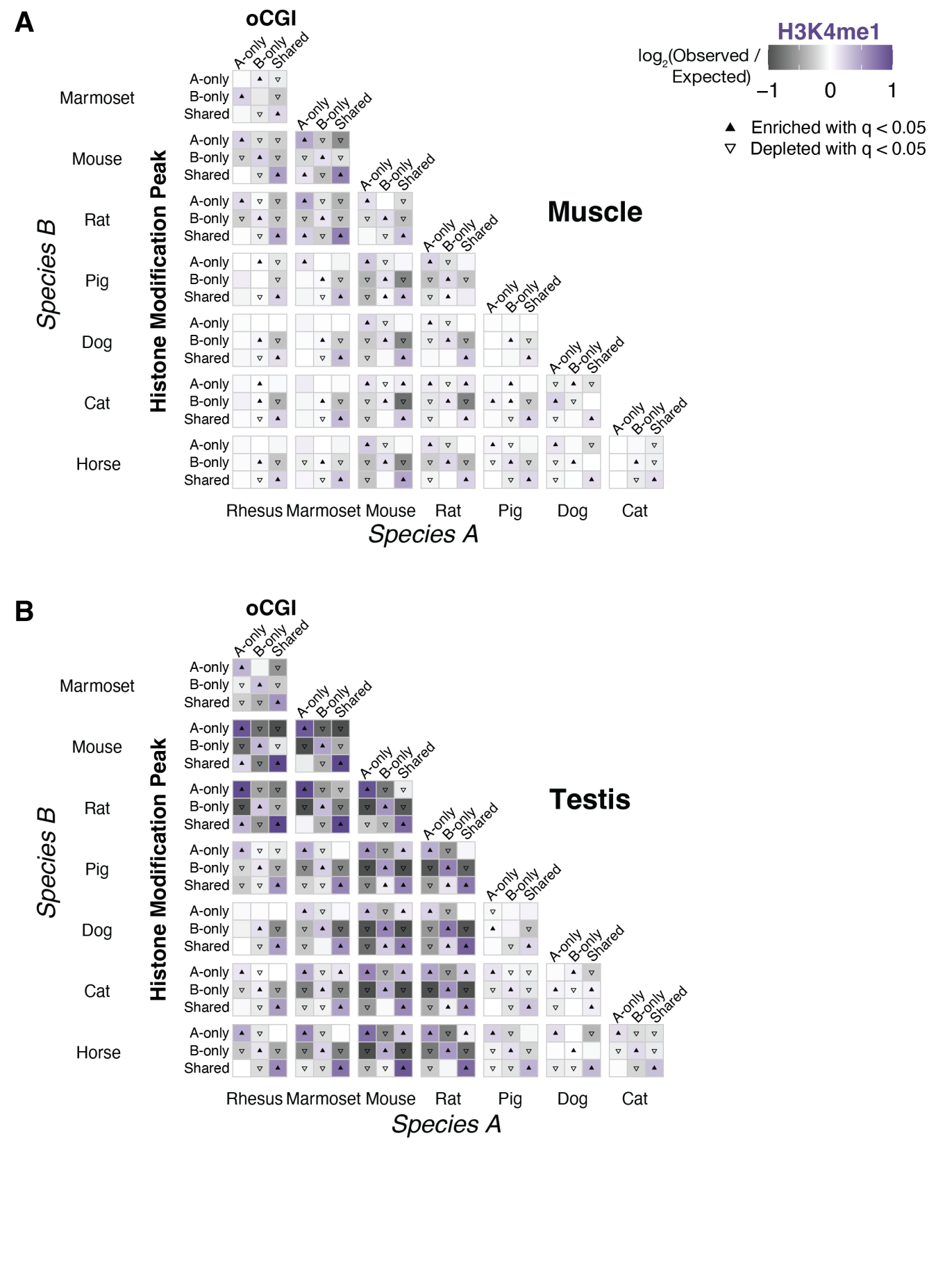


Fig S28. Species with oCGI predicts species with H3K4me1 peak (muscle and testis)

Enrichment and depletion in each indicated comparison of species-specific and shared oCGIs (*top:* A-only, B-only, Shared) and species-specific and shared peaks (*left*: A-only, B-only, Shared), compared to a null expectation of no association between oCGI turnover and peak turnover. Each 3 x 3 grid shows the results for a specific test examining oCGIs and their overlap with H3K4me1 in adult muscle (A) or testis (B) within a given species pair. Each box in each grid is colored according to the level of enrichment over expectation (purple) or depletion (gray) of genome-wide sites that meet the criteria for that box. The color bar illustrates the level of enrichment or depletion over expectation. For visualization, values greater than 1 and less than -1 were set to 1 and -1, respectively. The filled upward-pointing triangles denote significant enrichment and open downward-pointing triangles denote significant depletion (q < 0.05, permutation test, BH-corrected, see Fig. S22 and Methods).


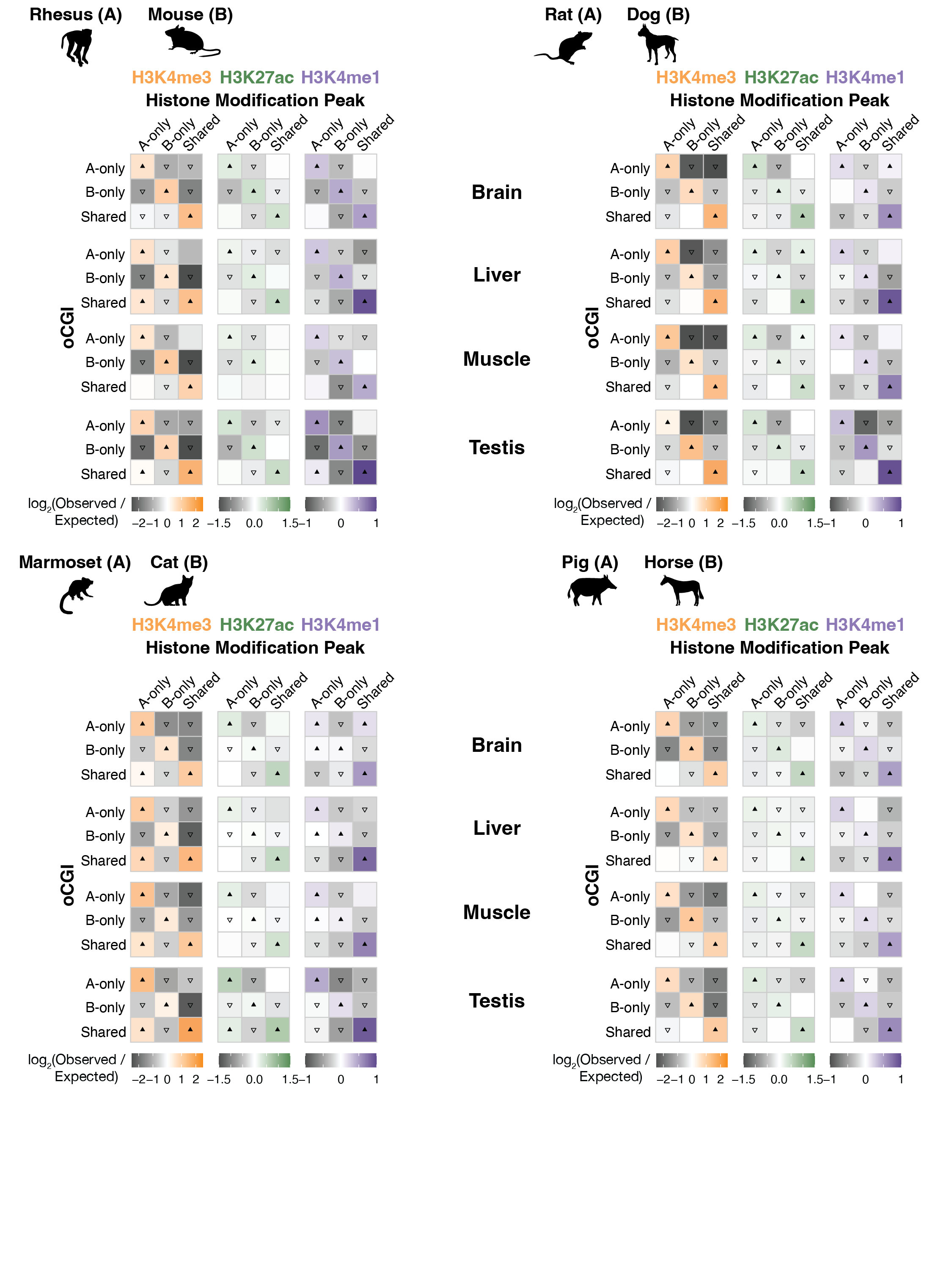


Fig S29. Species with peak predicts species with oCGI (peak-centric analysis)

Enrichment and depletion in each indicated comparison of species-specific and shared peaks (*top:* A-only, B-only, Shared) and species-specific and shared oCGIs (*left*: A-only, B-only, Shared), compared to a null expectation of no association between peak turnover and oCGI turnover. Each 3 x 3 grid shows the results for a specific test examining H3K4me3, H3K27ac, or H3K4me1 peaks and their overlap with oCGIs in adult brain, liver, muscle, or testis within a given species pair. Each box in each grid is colored according to the level of enrichment over expectation (orange for H3K4me3, green for H3K27ac, purple for H3K4me1) or depletion (gray for all marks) of genome-wide sites that meet the criteria for that box. The color bars illustrate the level of enrichment or depletion over expectation. For visualization, values greater than the scale maximum (2.5 for H3K4me3, 1.5 for H3K27ac, and 1 for H3K4me1) and less than the scale minimum (-2.5 for H3K4me3, -1.5 for H3K27ac, and -1 for H3K4me1) were set to the maximum or minimum, respectively. The filled upward-pointing triangles denote significant enrichment and open downward-pointing triangles denote significant depletion (q < 0.05, permutation test, BH-corrected, see Fig. S22 and Methods).


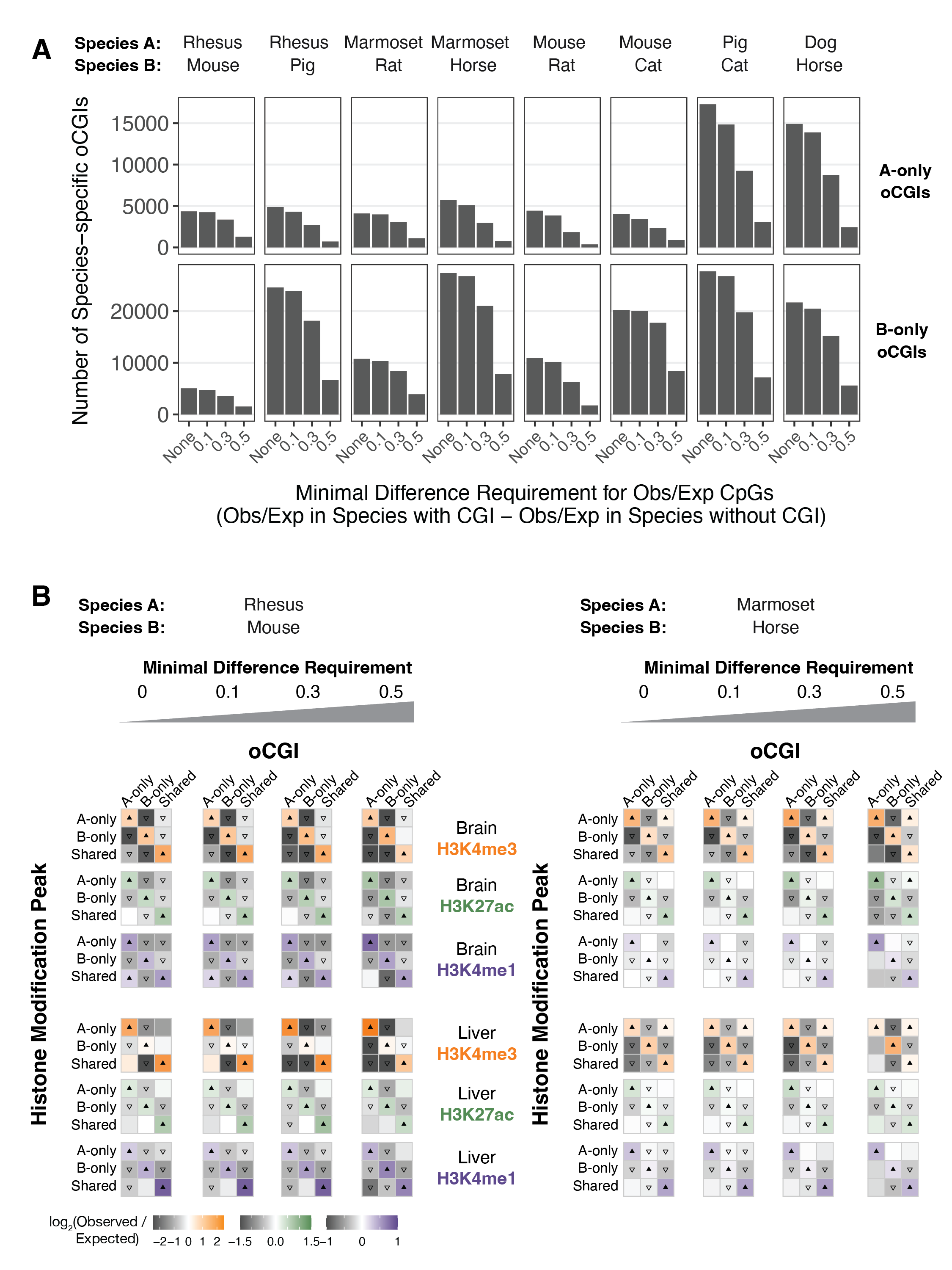


**Fig S30. Evaluating minimal CpG difference requirements for identifying species-specific oCGIs**

(A) Number of species-specific oCGIs identified for several representative species pairs using several difference minimal difference requirements (none, 0.1, 0.3, and 0.5) for the observed/expected CpG dinucleotide ratio. (B) Enrichment and depletion in each indicated comparison of species-specific and shared oCGIs (A-only, B-only, Shared) and species-specific and shared peaks (A-only, B-only, Shared), compared to a null expectation of no association between oCGI turnover and peak turnover. Species-specific oCGIs were filtered based on the minimal difference requirement listed for each column (ranging from none to 0.5). Each 3 x 3 grid shows the results for a specific test examining oCGIs and their overlap with a specific histone modification in a specific tissue (for example Brain H3K4me3) within a given species pair. Each box in each grid is colored according to the level of enrichment over expectation (orange for H3K4me3, green for H3K27ac, and purple for H3K4me1) or depletion (gray for all marks) of genome-wide sites that meet the criteria for that box. Color bars illustrate the level of enrichment or depletion over expectation. For visualization, values greater than the scale maximum (2.5 for H3K4me3, 1.5 for H3K27ac, and 1 for H3K4me1) and less than the scale minimum (-2.5 for H3K4me3, -1.5 for H3K27ac, and -1 for H3K4me1) were set to the maximum or minimum, respectively. The filled upward-pointing triangles denote significant enrichment and open downward-pointing triangles denote significant depletion (q < 0.05, permutation test, BH-corrected, see Fig. S22 and Methods).


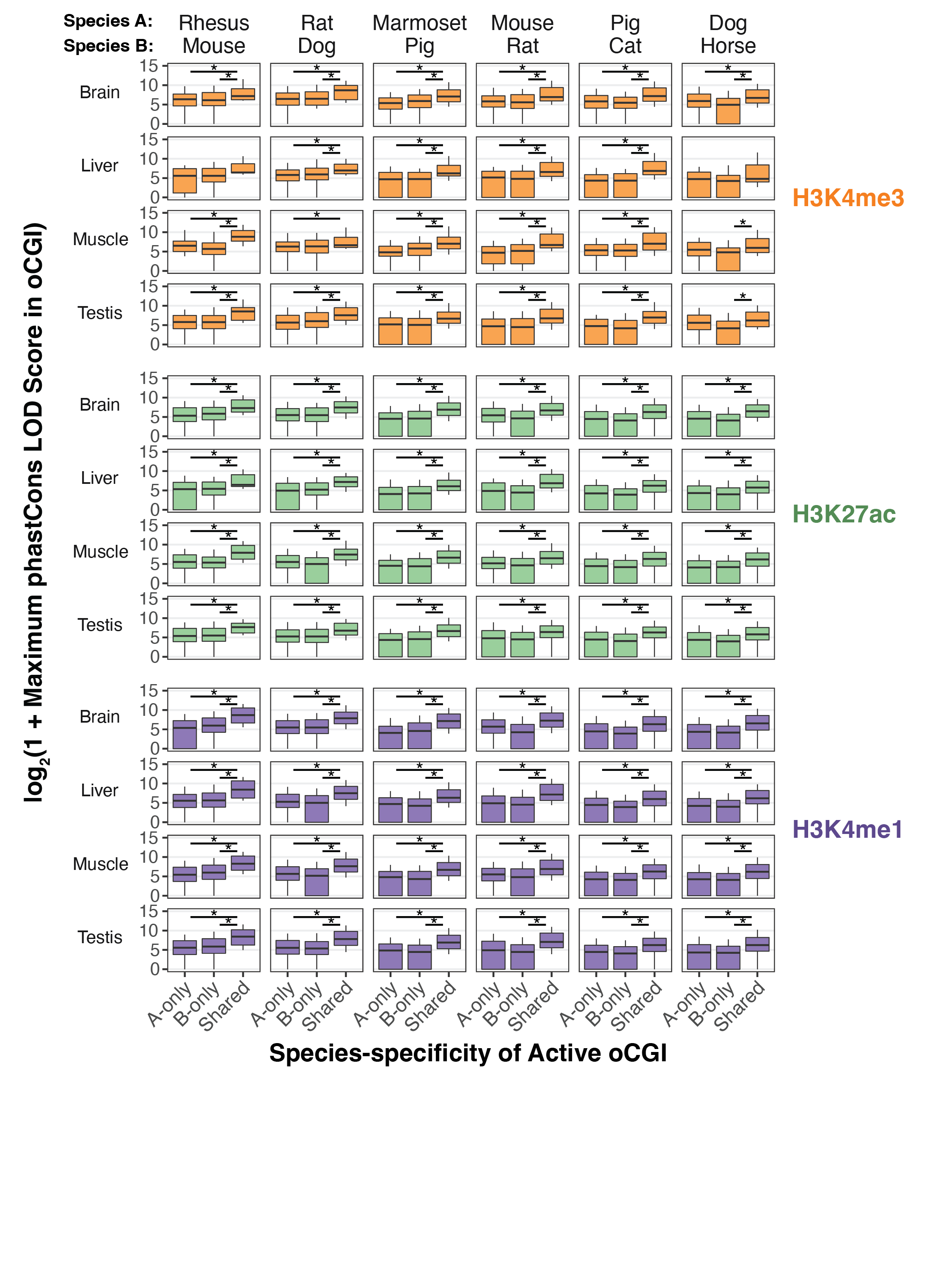


Fig S31. Shared active oCGIs have higher maximum phastCons LOD scores

Maximum phastCons LOD scores for species-specific oCGIs in species-specific peaks and shared oCGIs in shared peaks across all tissues and histone modifications for six species pairs. Box plots show the interquartile range and median, and whiskers indicate the 80% confidence interval. Stars indicate significant differences (q < 0.05 Wilcoxon rank-sum test, BH-corrected).


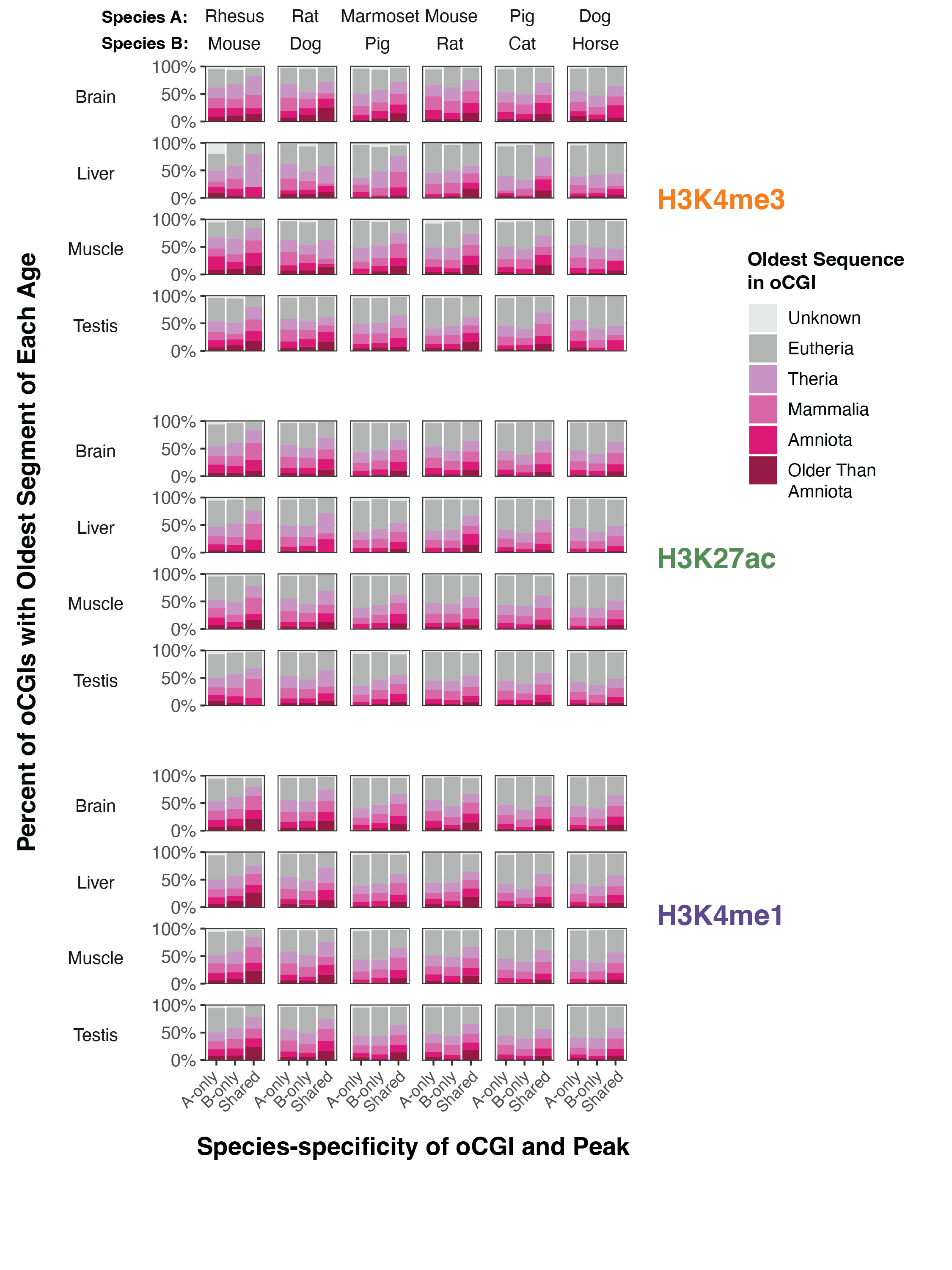


Fig S32. Shared active oCGIs are present at older sequences

The percent of species-specific oCGIs in species-specific peaks and shared oCGIs in shared peaks whose oldest sequence belongs to each age category (see legend). Results are shown for all tissues and histone modifications for six species pairs.


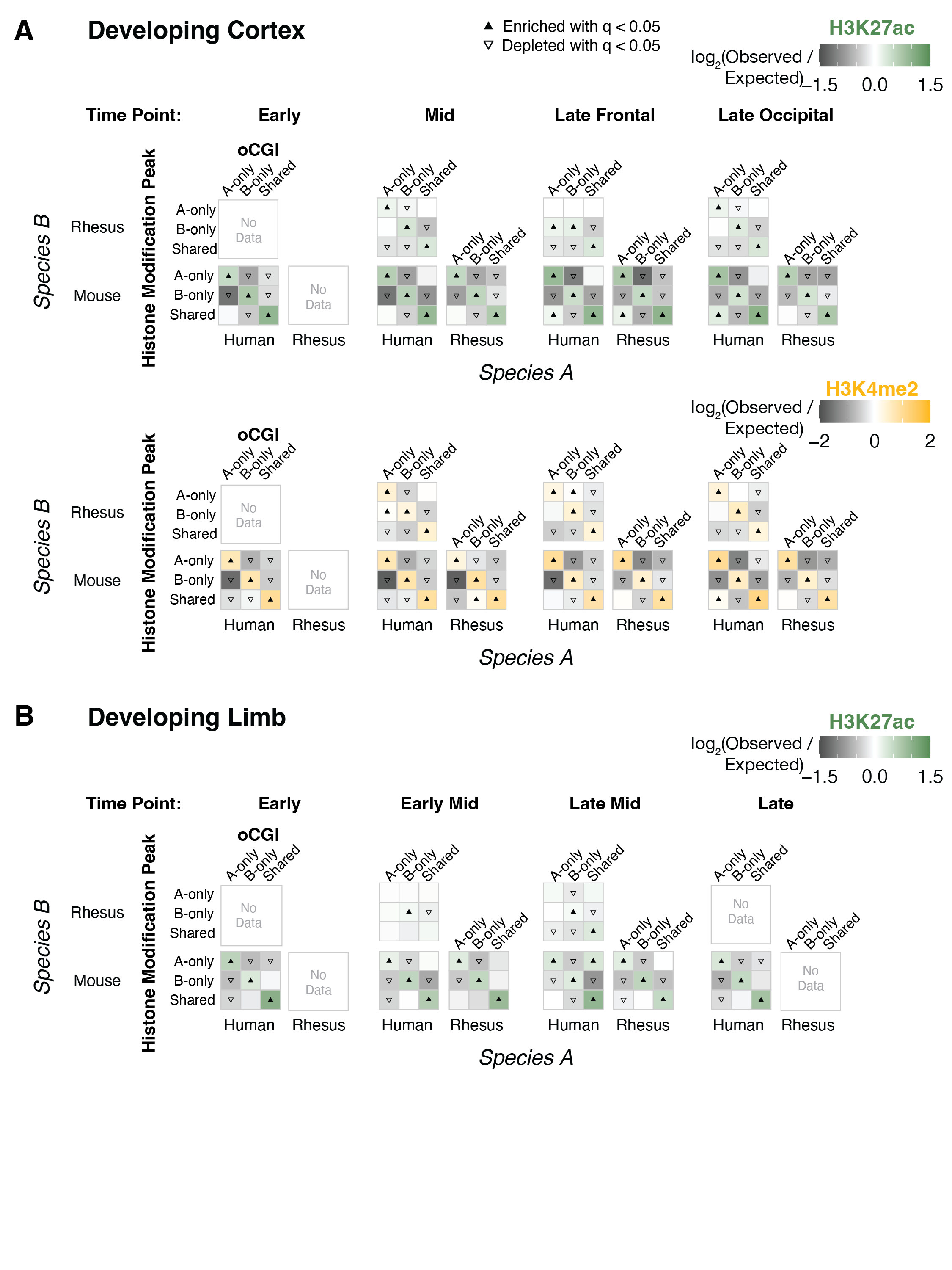


Fig S33. oCGIs predict histone modification peaks in developing tissues

(A) Enrichment and depletion in each indicated comparison of species-specific and shared oCGIs (*top:* A-only, B-only, Shared) and species-specific and shared peaks (*left*: A-only, B-only, Shared), compared to a null expectation of no association between oCGI turnover and peak turnover. Each 3 x 3 grid shows the results for a specific test examining oCGIs and their overlap with H3K27ac or H3K4me2 in developing cortex within a given species pair. Each box in each grid is colored according to the level of enrichment over expectation (green for H3K27ac, yellow for H3K4me2) or depletion (gray for both marks) of genome-wide sites that meet the criteria for that box. The color bars illustrate the level of enrichment or depletion over expectation. For visualization, values greater than the scale maximum (1.5 for H3K27ac, 2 for H3K4me2) and less than the scale minimum (-1.5 for H3K27ac, -2 for H3K4me2) were set to the scale maximum or minimum, respectively. The filled upward-pointing triangles denote significant enrichment and open downward-pointing triangles denote significant depletion (q < 0.05, permutation test, BH-corrected, see Fig. S22 and Methods). Developmental stages in each species for each time point are shown in Table S5. (B) Enrichment and depletion in each indicated comparison of species-specific and shared oCGIs (*top:* A-only, B-only, Shared) and species-specific and shared peaks (*left*: A-only, B-only, Shared), shown as in (A) but using H3K27ac peak data from developing limb.

**
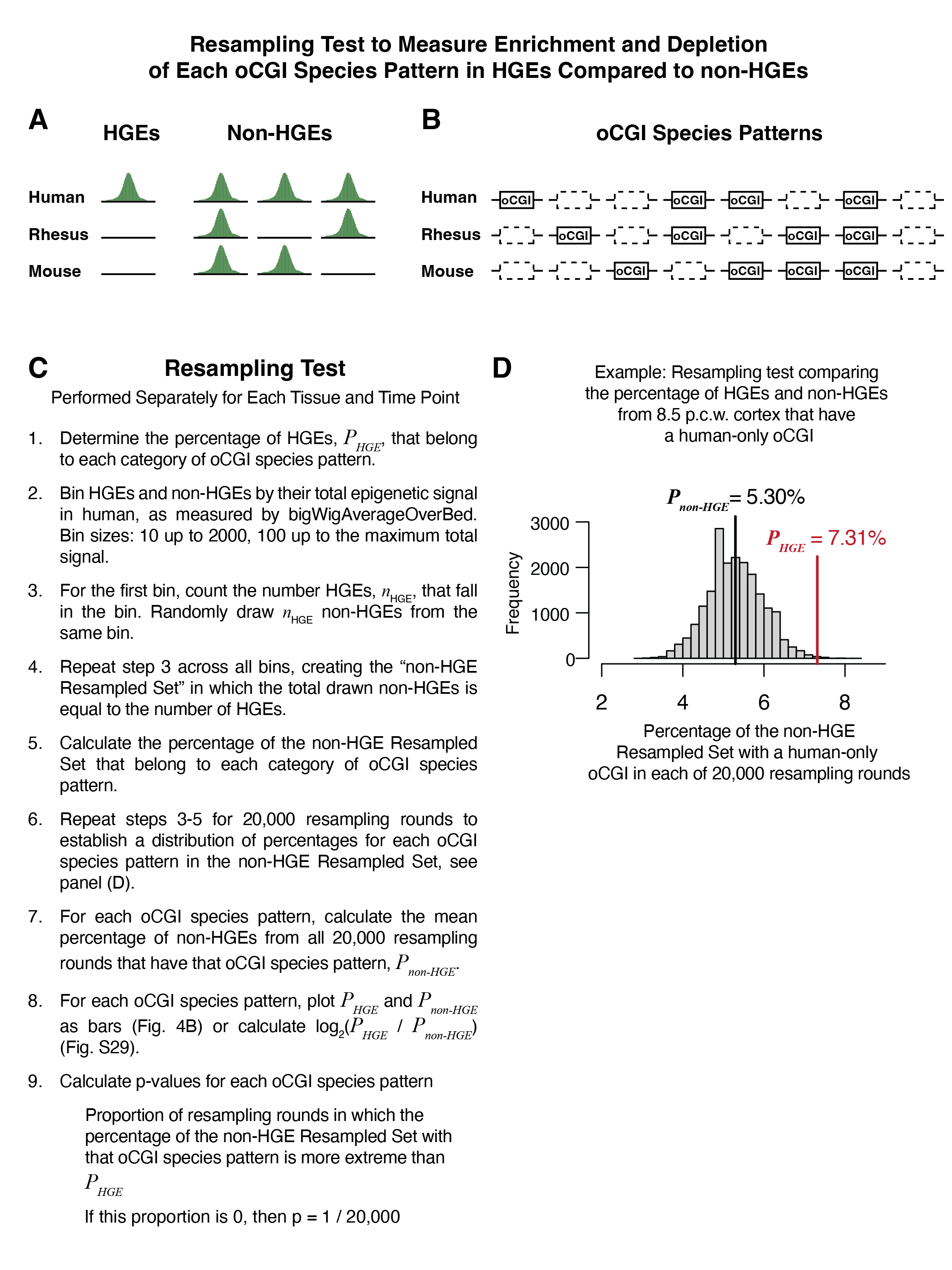
**

**Fig S34. Resampling test for analysis of oCGI species patterns in HGEs**

Explanation of resampling test to determine enrichment and depletion of each oCGI species pattern in HGEs compared to non-HGE human enhancers. (A) Schematic showing H3K27ac ChIP-seq peaks in human, rhesus macaque, and mouse for HGEs (*left*) compared to non-HGE human enhancers (*right*). (B) The oCGI species patterns considered in this analysis. Boxes with solid lines indicate oCGIs, and boxes with dashed lines indicate orthologous sequence that does not contain an oCGI. (C) Explanation of the resampling test used to compare HGEs to activity-matched non-HGEs. (D) Example results from a resampling test comparing the percent of HGEs that have a human-only oCGI to activity-matched non-HGE enhancers, using data from human cortex at 8.5 post-conception weeks (p.c.w.).


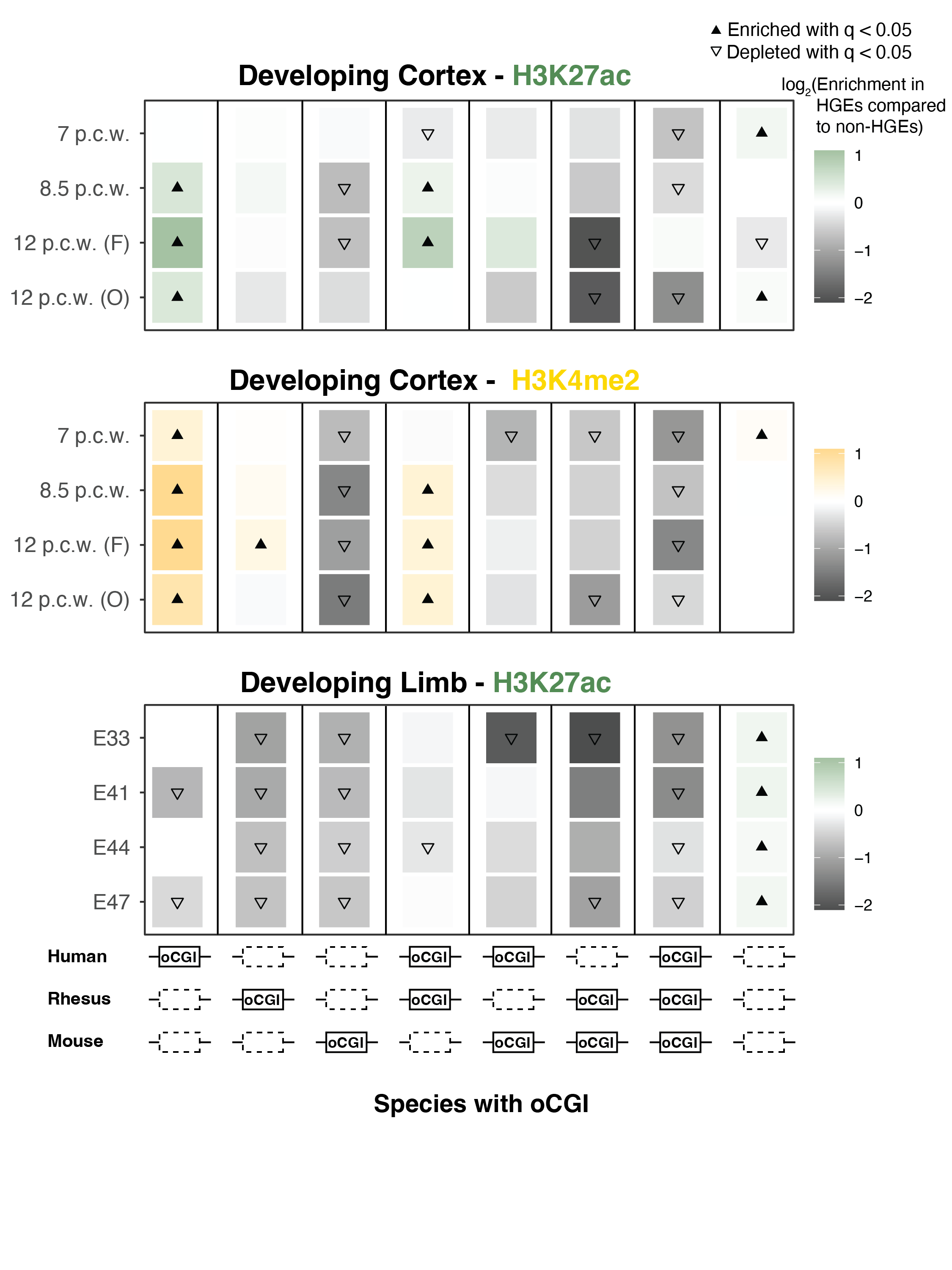


Fig S35. oCGI patterns enriched and depleted in HGEs

Enrichment of each specified oCGI pattern (*bottom*) in HGEs compared to activity-matched non-HGE human enhancers, across all human tissues, marks, and time points. Boxes are colored according to their enrichment (green for H3K27ac, yellow for H3K4me2) or depletion (gray for both marks). The filled upward-pointing triangles denote significant enrichment and open downward-pointing triangles denote significant depletion (q < 0.05, resampling test comparing HGEs to activity-matched non-HGEs, BH-corrected; see Methods and Fig. S34). p.c.w = post-conception weeks, F = frontal region, O = occipital region, E = embryonic day.


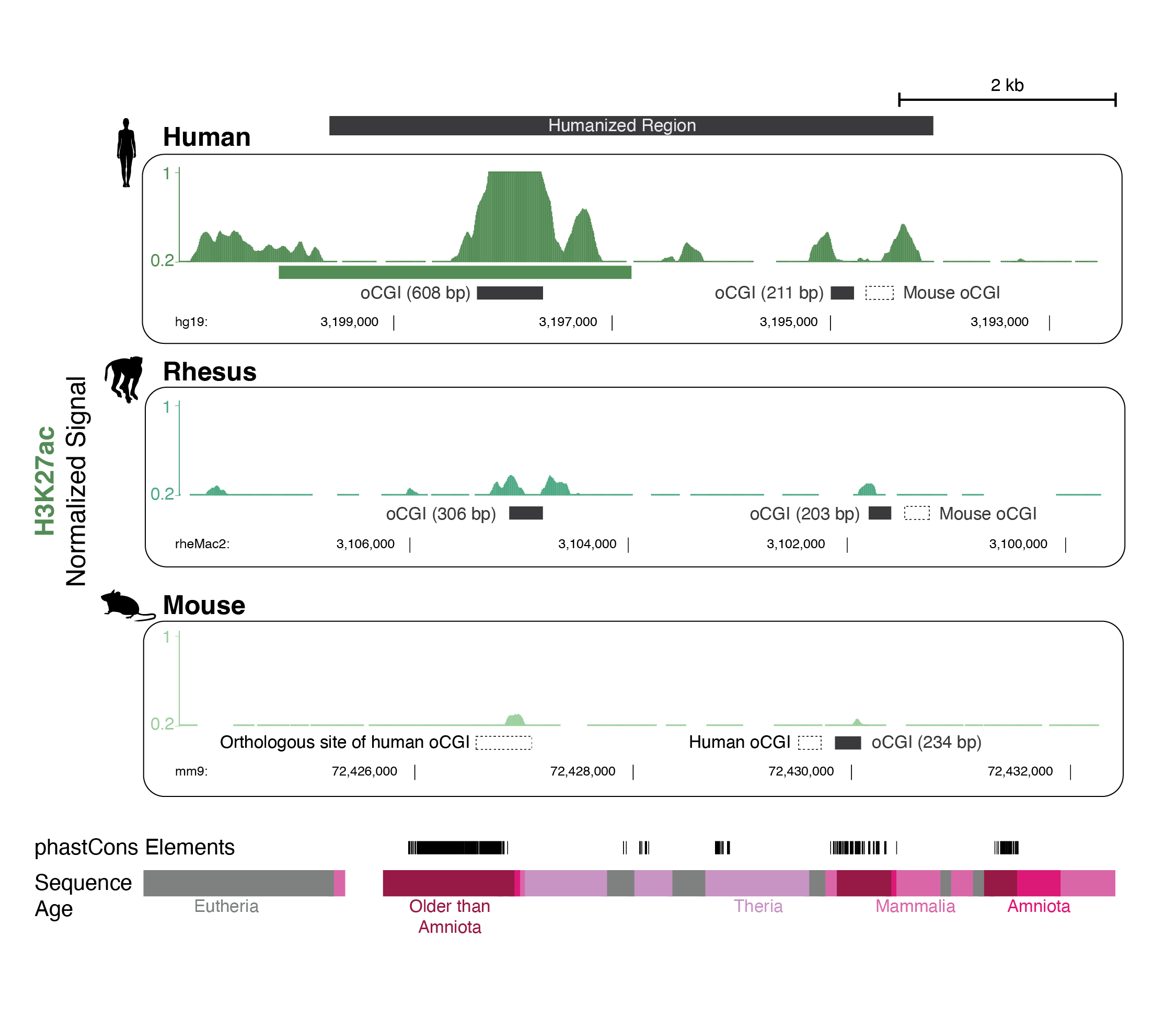


**Fig S36. The Human Gain Enhancer hs754**

H3K27ac levels are shown in developing cortex at human 8.5 p.c.w., rhesus embryonic day 55, and mouse embryonic day 14.5 [36]. H3K27ac signal tracks show the number of sequenced fragments per million overlapping each base pair. Black bars denote the locations of oCGIs in each species, and empty bars with dotted lines denote the locations where an orthologous sequence in another species contains an oCGI. Additional tracks show the locations of phastCons elements and sequences of the indicated evolutionary origin. For the purposes of visualization, features in rhesus and mouse have been aligned to the location of an orthologous base pair within the human peak due to overall differences in orthologous sequence lengths.


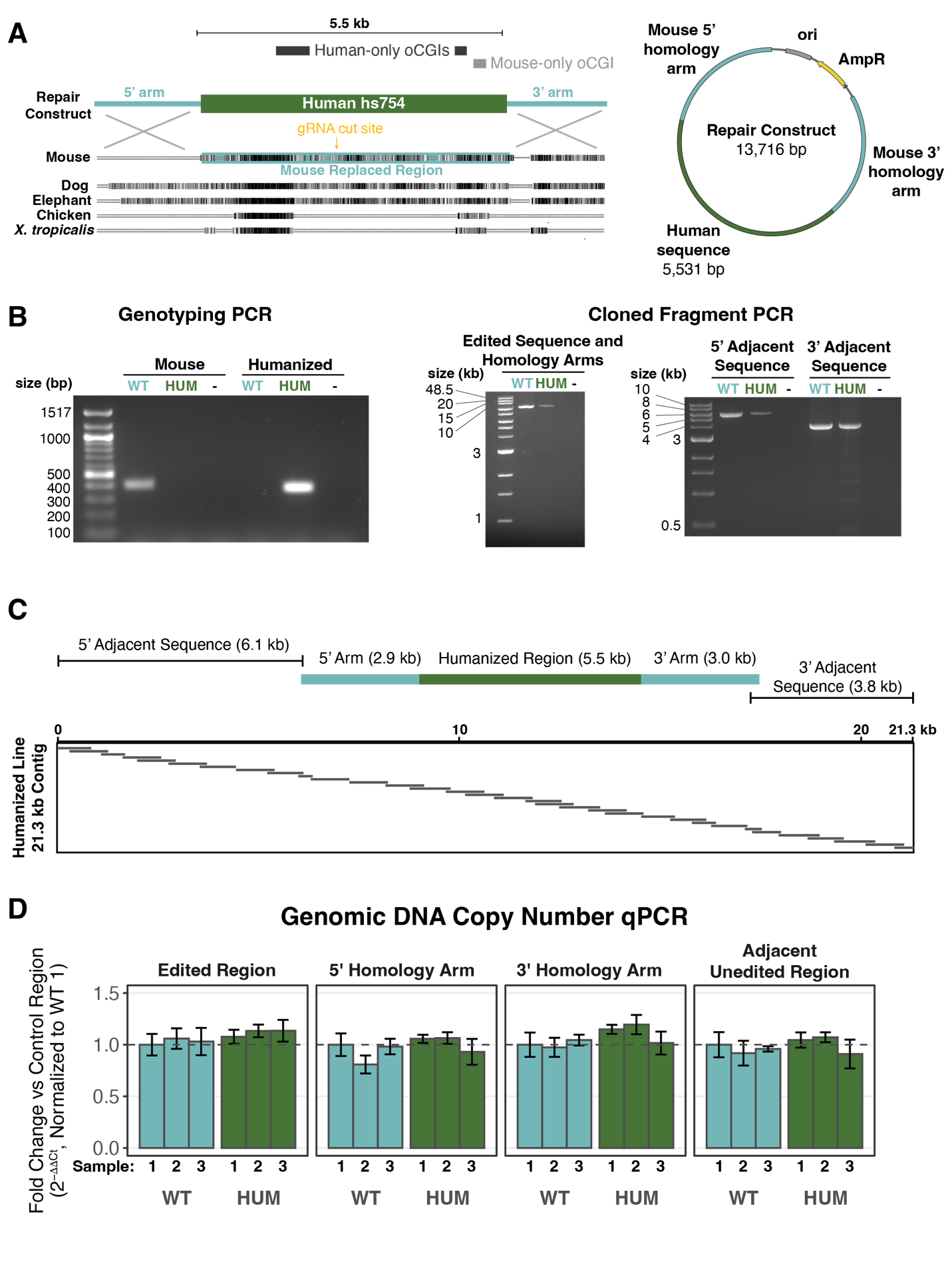


Fig S37. Generation and validation of the hs754 humanized mouse model hs754

(A) *Left*: schematic of the humanized locus with human-based alignment to mouse, dog, elephant, chicken, and *Xenopus tropicalis* showing the humanized region, its highly conserved core sequence, and the location of the gRNA cut site. *Right*: the repair construct used to generate the humanized mouse line. (B) *Left*: Example genotyping PCR. Center: Amplification across the 5’ homology arm, the humanized region, and the 3’ homology arm. *Right*: Amplification of the mouse sequence adjacent to the 5’ and 3’ homology arms. (C) Alignment of Sanger sequencing reads across a 21.3-kb region centered on the humanized region on chromosome 13. (D) Genomic DNA copy number qPCR for the edited region, the homology arms, and an adjacent unedited region, normalized to a control region on chromosome 5. WT = wild type, HUM = humanized.


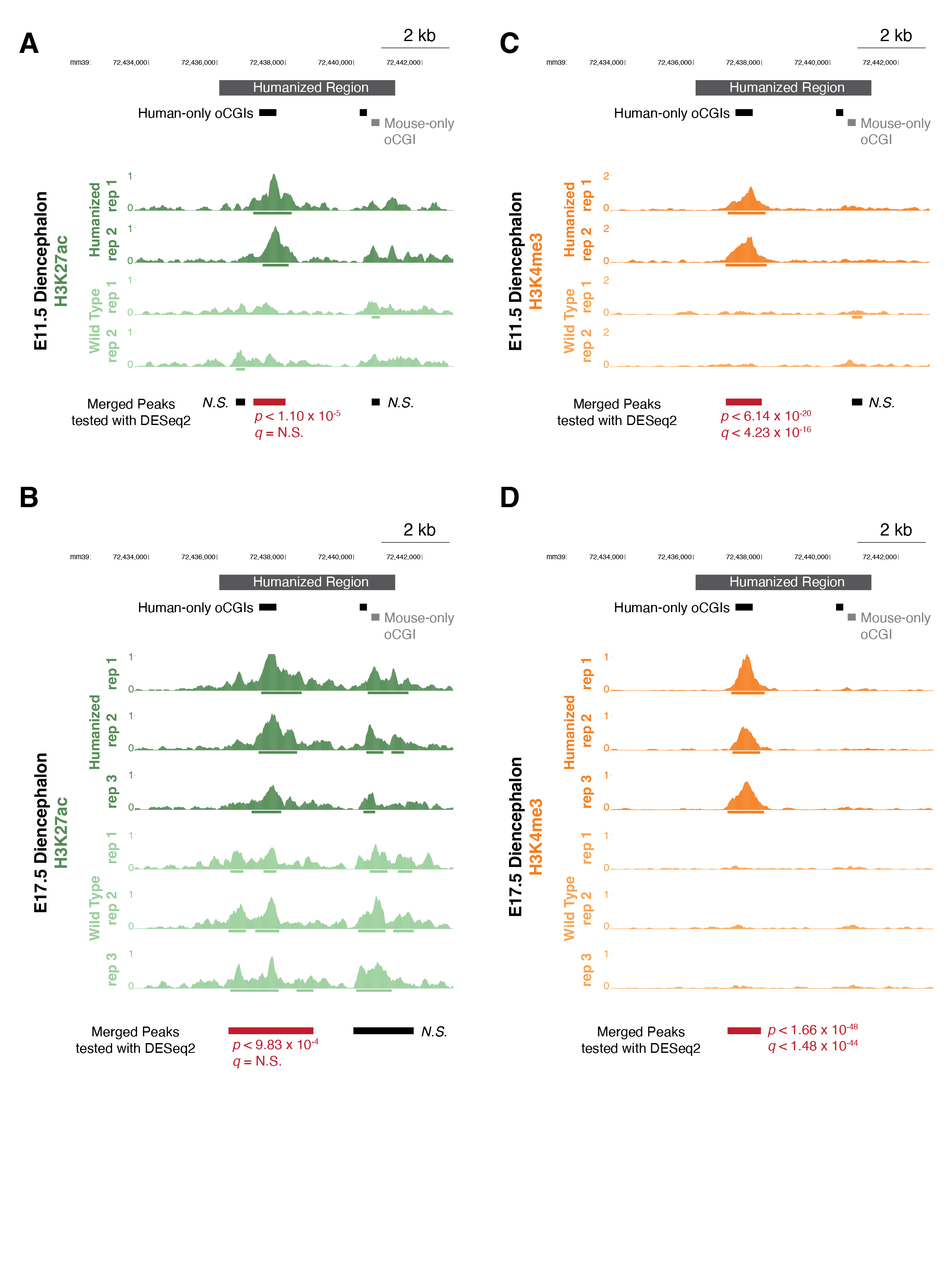


**Fig S38. Gain of H3K27ac and H3K4me3 associated with a human oCGI in a humanized mouse model, data for all replicates**

(A) H3K27ac levels for the humanized (dark green) and wild type (light green) mouse in embryonic day 11.5 (E11.5) diencephalon. Signal tracks display read counts per million in adjacent 10-bp bins. Bars under peaks show peak calls within each replicate. Merged peaks at the bottom show the union of peaks across all replicates and genotypes (shown in wild type coordinates), which were tested for differential levels between genotypes using DESeq2. Significance was determined using a Wald test to identify p-values, which were BH-corrected to q-values (both are shown). The humanized tracks have been shifted 190 bp to the left to align an orthologous base within the oCGI, due to overall differences in orthologous sequence lengths. (B) H3K27ac levels in E17.5 diencephalon, shown as in (A). (C) H3K4me3 levels in E11.5 diencephalon, shown as in (A) but with humanized tracks in dark orange and wild type tracks in light orange. (D) H3K4me3 levels in E17.5 diencephalon, shown as in (A) but with humanized tracks in dark orange and wild type tracks in light orange.


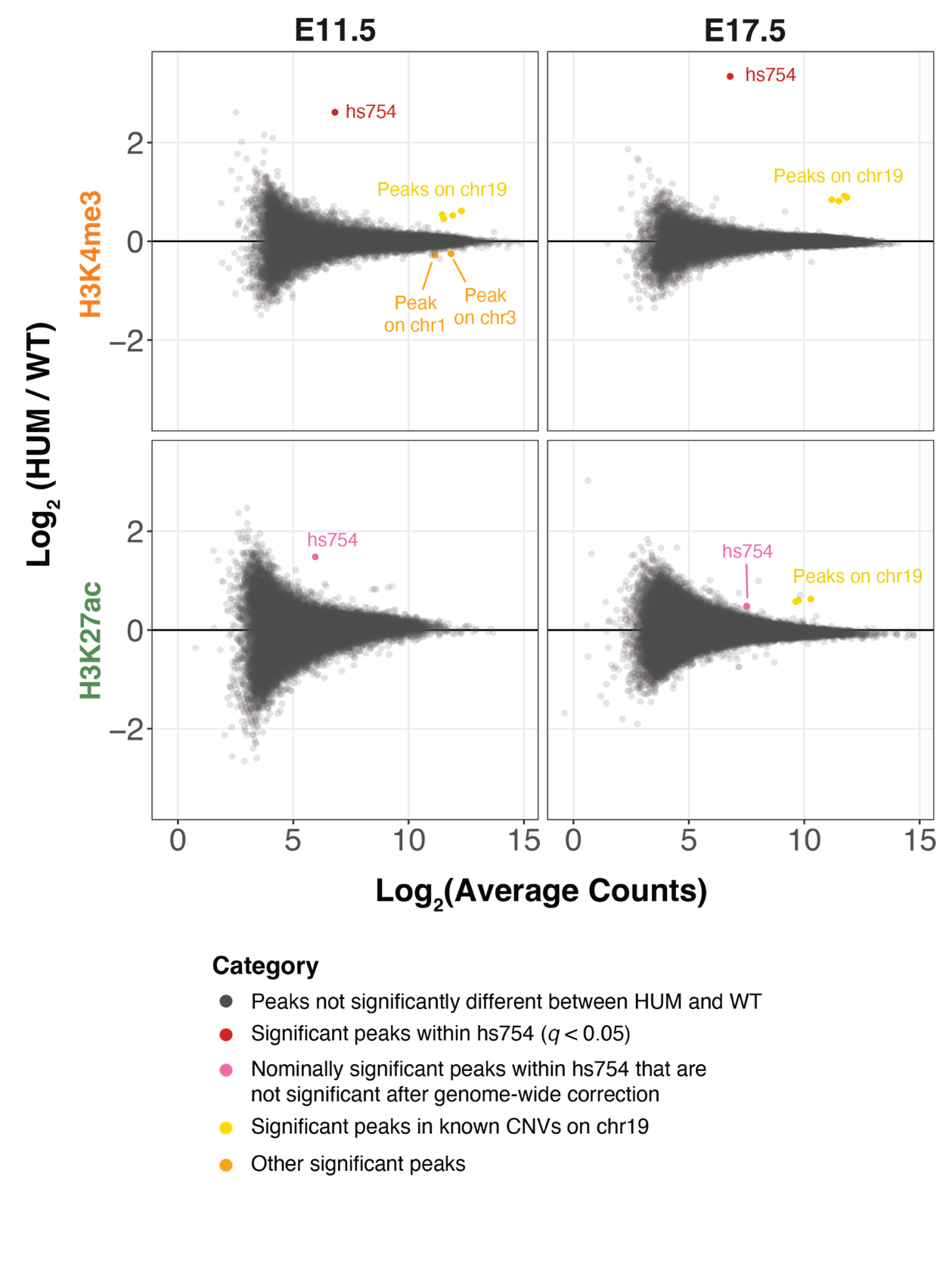


Fig S39. Genome-wide analysis of differential histone modification levels in the hs754 humanized mouse versus wild type

Log_2_-transformed average counts across all replicates and genotypes versus the log_2_-transformed ratio of normalized reads between humanized (HUM) and wild type (WT) mouse diencephalon, at time points E11.5 (*left*) and E17.5 (*right*), for H3K4me3 (*top*) and H3K27ac (*bottom*). Differential peaks are indicated (see legend and Methods). See Fig. S51 for additional information on the peaks on chr 19.


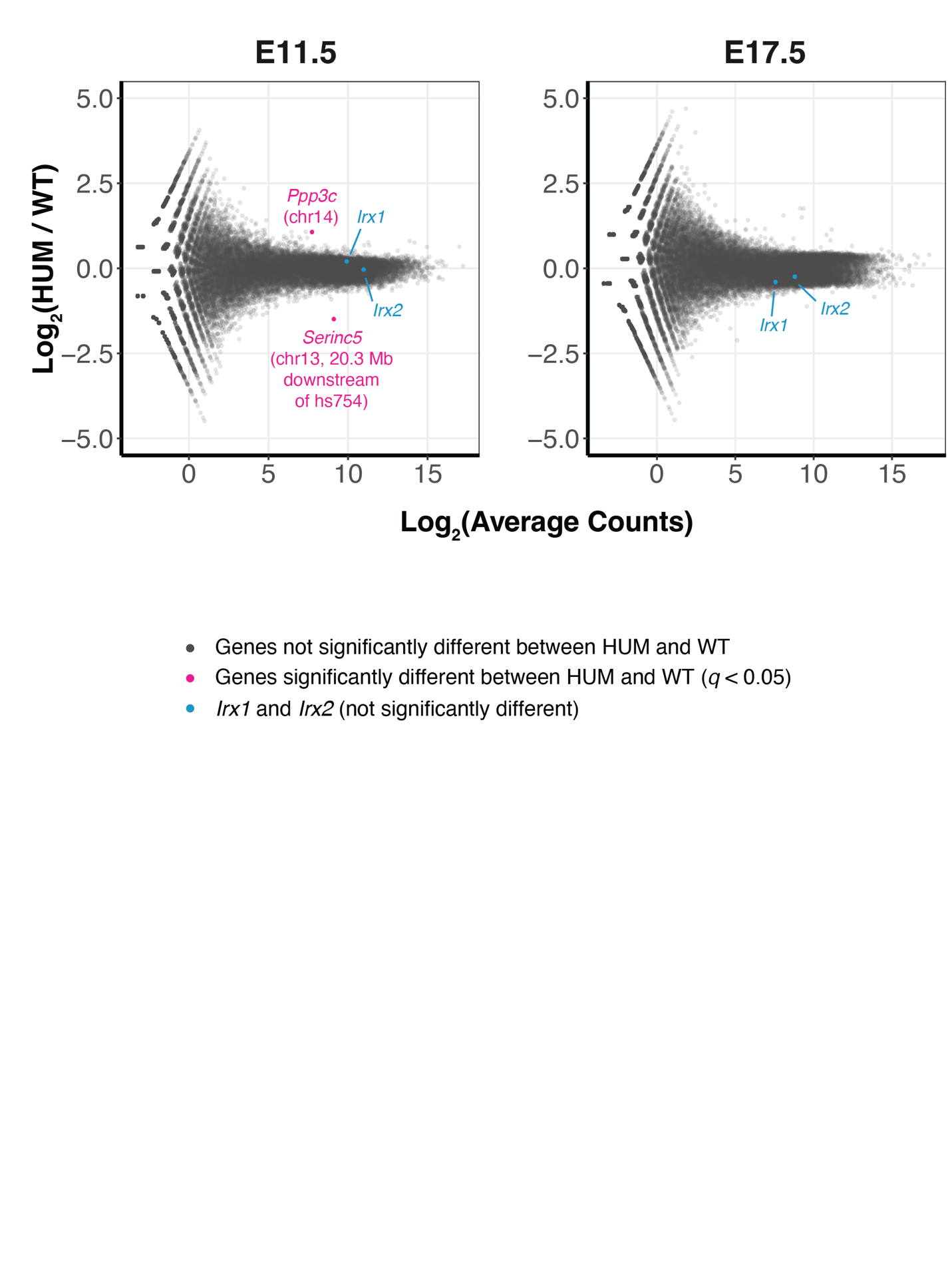


Fig S40. Genome-wide analysis of differential gene expression in the hs754 humanized mouse versus wild type

Log_2_-transformed Average Counts across all replicates and genotypes versus the Log_2_-transformed ratio of normalized reads between humanized (HUM) and wild type (WT) diencephalon at E11.5 (*left*) and E17.5 (*right*). All genes are shown in gray, with differentially expressed genes indicated in pink (see Methods). The putative target genes on either side of hs754 (*Irx1* and *Irx2*) are indicated in blue and are not called as differentially expressed.


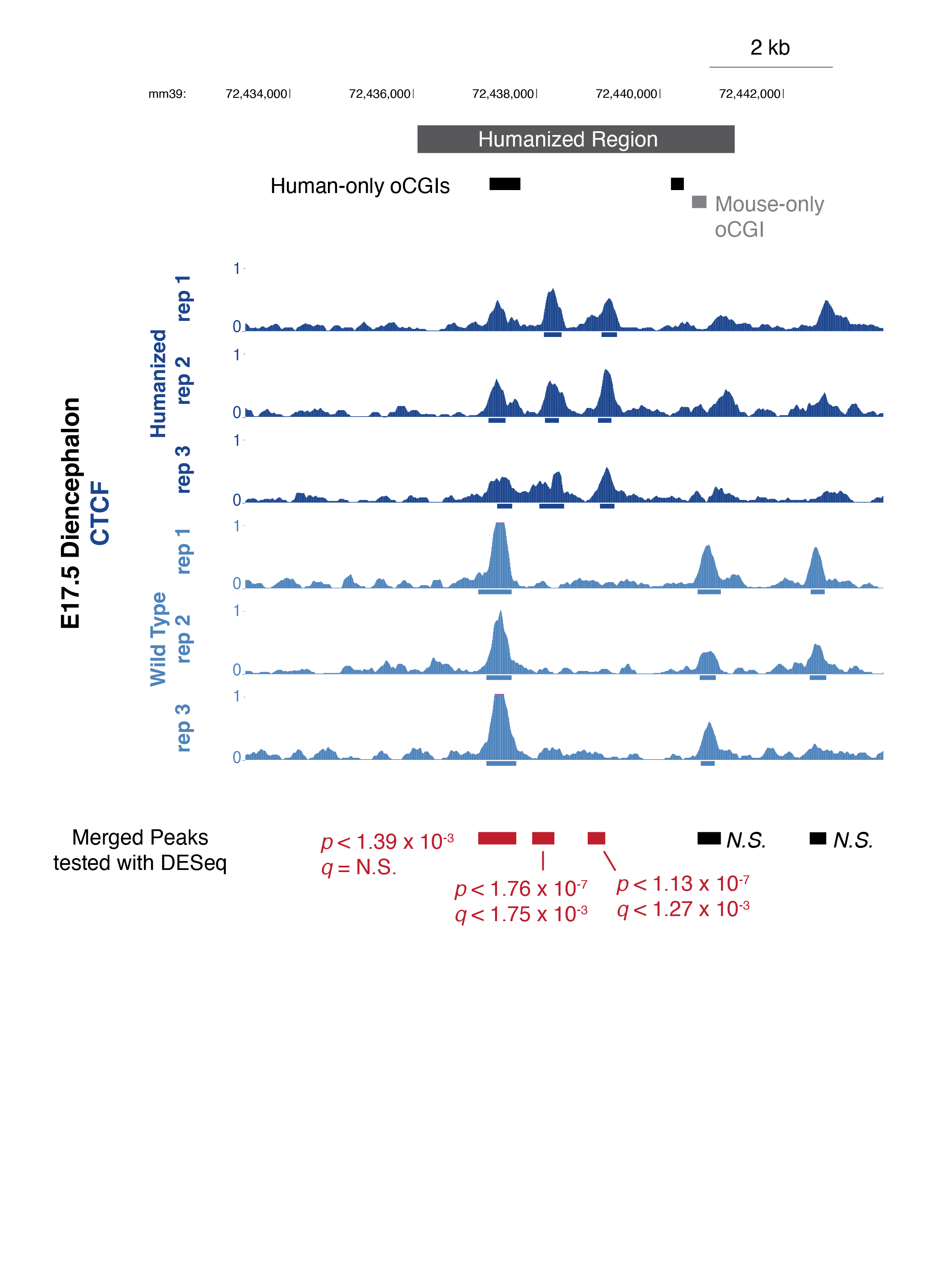


Fig S41. The humanized locus recruits two additional CTCF binding events

CTCF binding levels in developing diencephalon at embryonic day 17.5 (E17.5) from the humanized (dark blue) and wild type (light blue) mouse. Signal tracks display read counts per million in adjacent 10-bp bins. Bars under peaks show peak calls within each replicate. Merged peaks at the bottom show the union of peaks across all replicates and genotypes (shown in wild type coordinates), which were tested for differential signal between genotypes using DESeq2 (see Methods). Significance was determined using a Wald test to identify p-values, which were BH-corrected to q-values (both are shown). The humanized tracks have been shifted 190 bp to the left to align an orthologous base within the oCGI, due to overall differences in orthologous sequence lengths.


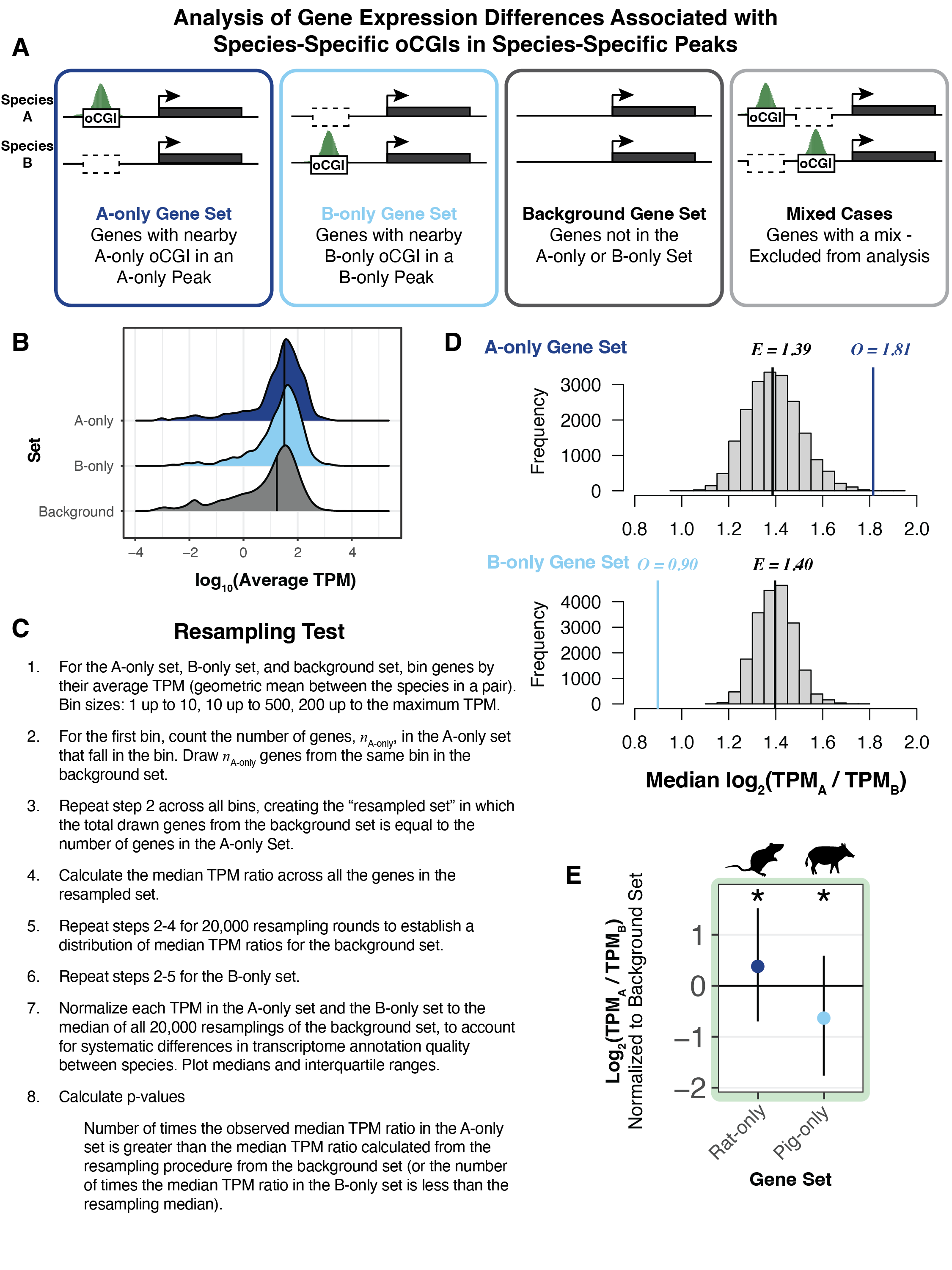


Fig S42. Analysis pipeline for species-specific active oCGIs and gene expression

(A) Gene sets defined in this analysis based on their linear proximity to species-specific oCGIs in species-specific peaks. (B) Density plot showing the average TPM of genes in the A-only, B-only, and background sets using H3K27ac data from adult brain in rat versus pig. Medians are indicated with vertical black lines. The medians for the A-only and B-only set are higher than the background set, motivating the use of a binned resampling test to compare the A-only set and B-only set to the background set, as explained in the remainder of the figure. (C) Explanation of binned resampling test to compare the medians of the A-only set and the B-only set to expected values generated by expression-matched resampling from the background set. (D) Example resampling results for the comparison between rat and pig for genes associated with species-specific oCGIs in species-specific H3K27ac peaks in adult brain. Histograms show the distribution of median log_2_-transformed TPM ratios across 20,000 resampling rounds, in each of which expression-matched genes were drawn from the background set. (E) Example results plot for the same comparison as in panel (D), between rat and pig using H3K27ac peaks from adult brain. All values in the rat-only set and pig-only set are normalized to the median of 20,000 resampling medians from the background set.


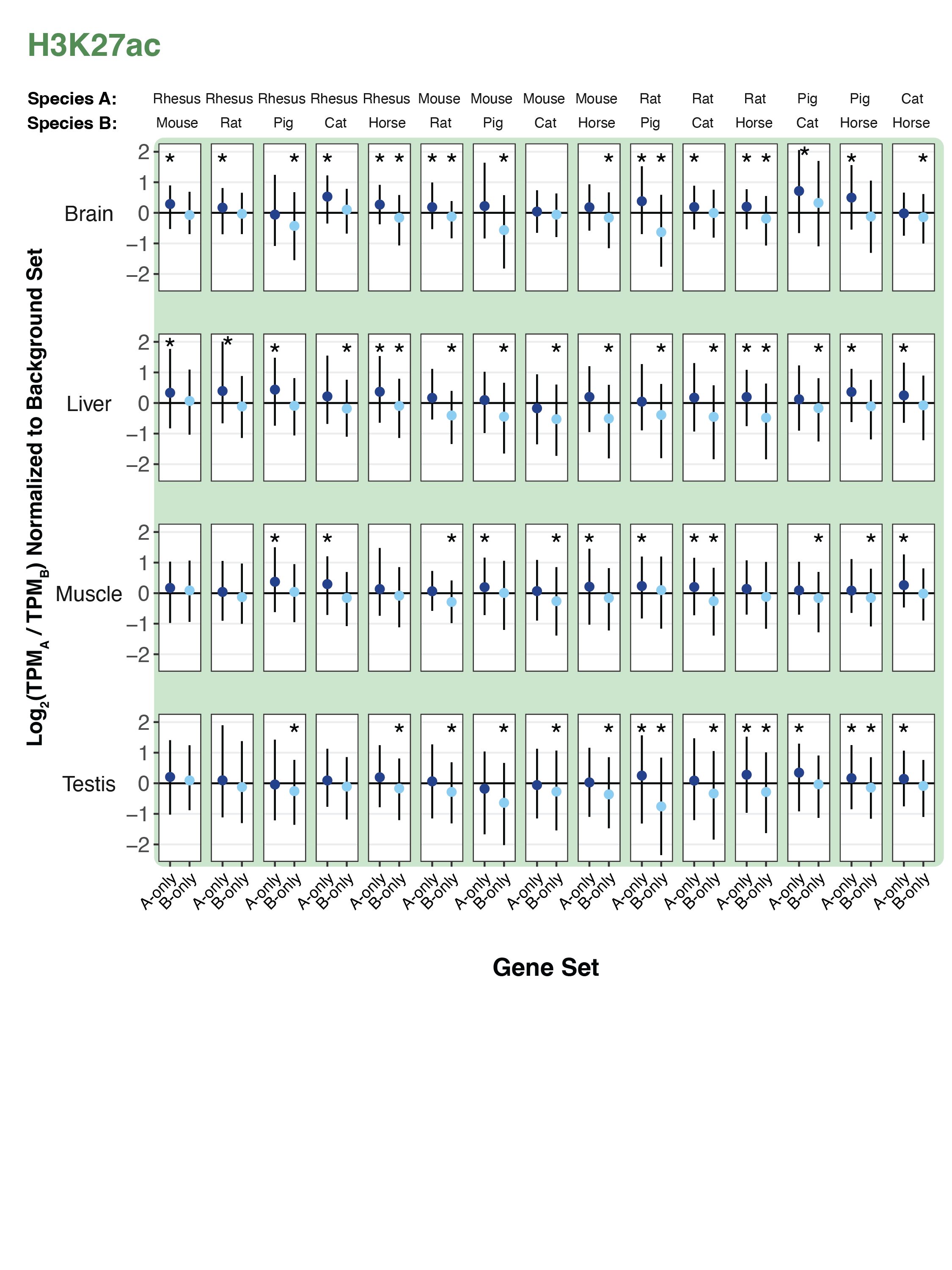


Fig S43. Species-specific oCGIs in species-specific H3K27ac peaks are associated with gene expression changes

The TPM ratio between species A and species B for genes in the A-only set and the B-only set. Results are shown for all species pairs and adult tissues, when genes are sorted based on nearby species-specific oCGIs in species-specific H3K27ac peaks. Points indicate median values for genes in the A-only set (dark blue) and genes in the B-only set (light blue). Lines indicate the interquartile range. All values in the A-only set and B-only set are normalized to the median of 20,000 resampling medians from the background set. Stars indicate a significant difference between the observed median and the expected median (q < 0.05, resampling test to compare to the background set, see Fig. S42 and Methods).


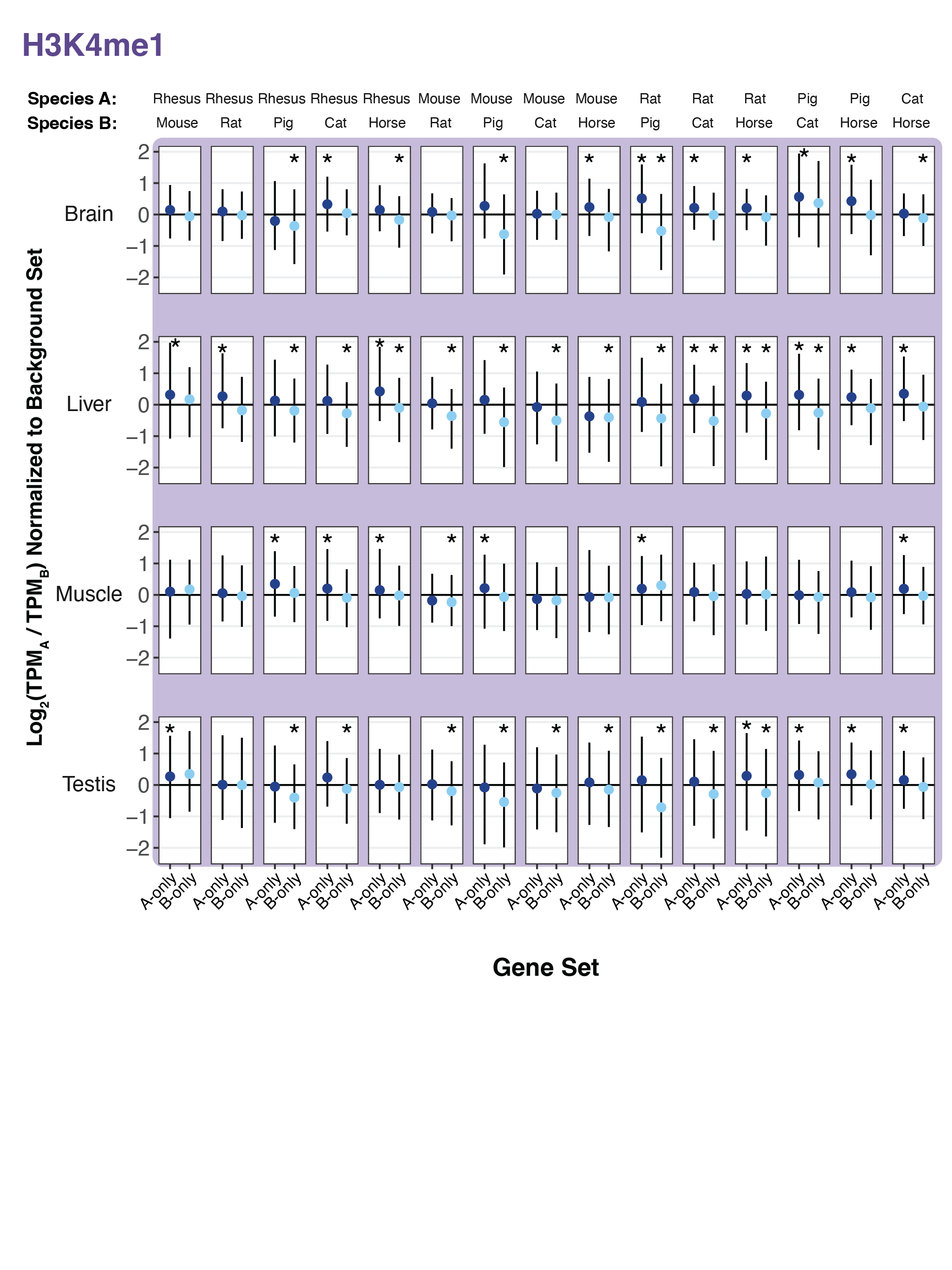


Fig S44. Species-specific oCGIs in species-specific H3K4me1 peaks are associated with gene expression changes

The TPM ratio between species A and species B for genes in the A-only set and the B-only set. Results are shown for all species pairs and adult tissues, when genes are sorted based on nearby species-specific oCGIs in species-specific H3K4me1 peaks. Points indicate median values for genes in the A-only set (dark blue) and genes in the B-only set (light blue). Lines indicate the interquartile range. All values in the A-only set and B-only set are normalized to the median of 20,000 resampling medians from the background set. Stars indicate a significant difference between the observed median and the expected median (q < 0.05, resampling test to compare to the background set, see Fig. S42 and Methods).


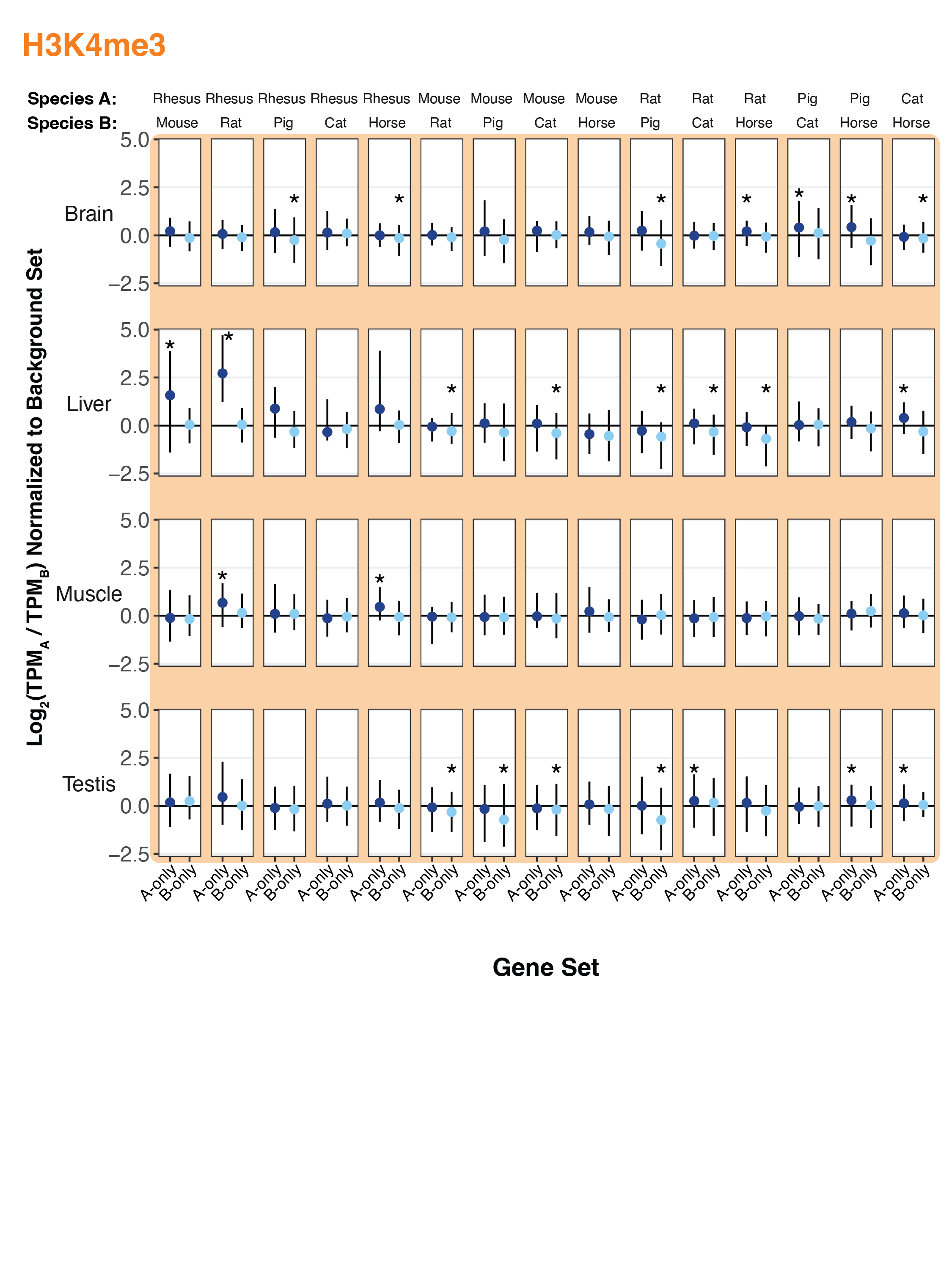


Fig S45. Species-specific oCGIs in species-specific H3K4me3 peaks are associated with gene expression changes

The TPM ratio between species A and species B for genes in the A-only set and the B-only set. Results are shown for all species pairs and adult tissues, when genes are sorted based on nearby species-specific oCGIs in species-specific H3K4me3 peaks. Points indicate median values for genes in the A-only set (dark blue) and genes in the B-only set (light blue). Lines indicate the interquartile range. All values in the A-only set and B-only set are normalized to the median of 20,000 resampling medians from the background set. Stars indicate a significant difference between the observed median and the expected median (q < 0.05, resampling test to compare to the background set, see Fig. S42 and Methods).


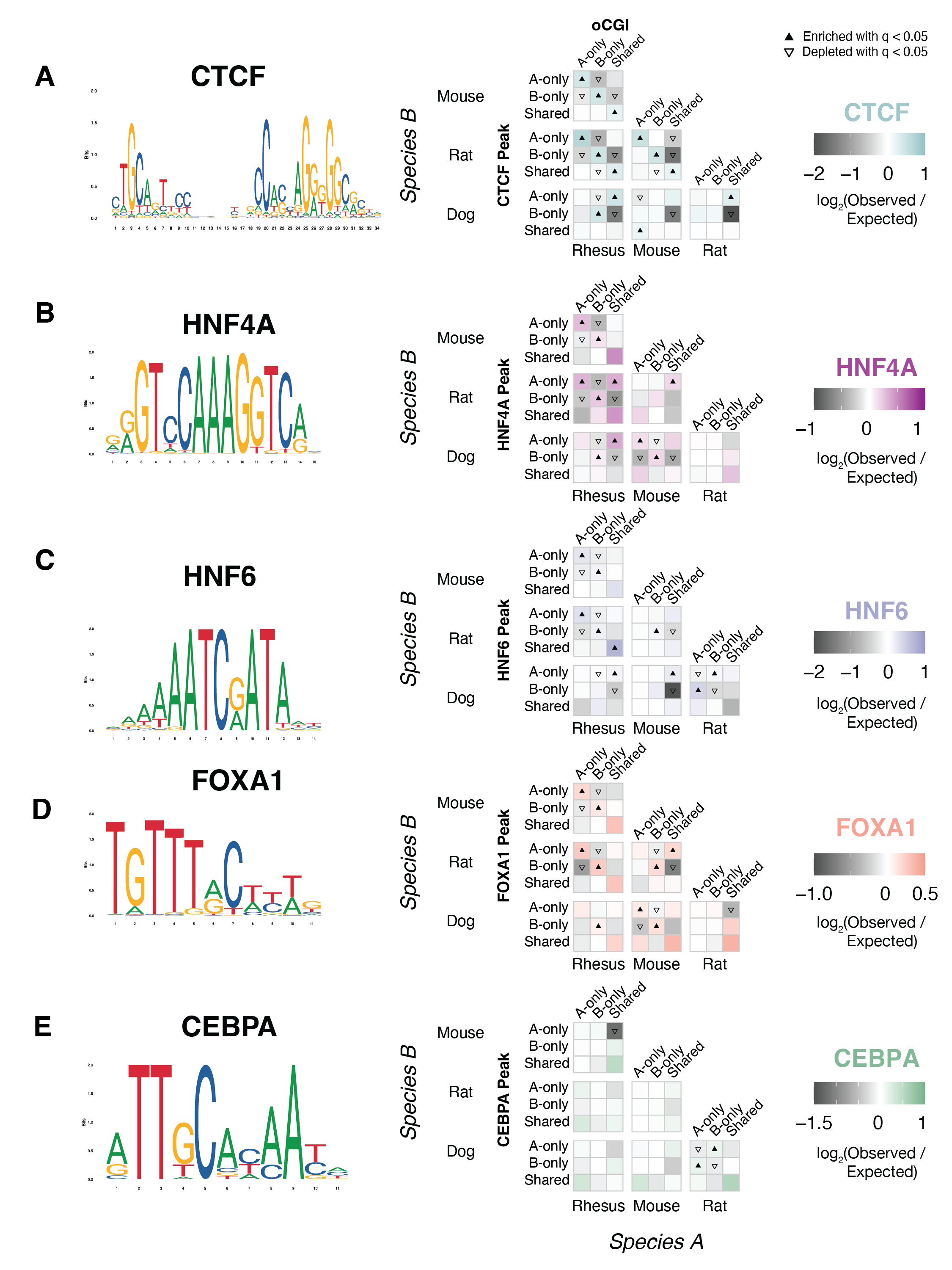


Fig S46. Enrichment and depletion results for all investigated TFs

*Left*: consensus motifs for each TF. Motifs are from the JASPAR database. CTCF: MA1929.1, FOXA1: MA0148.1, HNF4A: MA1494.1, HNF6: MA0679.1, CEBPA: MA0102.3. *Right*: Enrichment and depletion in each indicated comparison of species-specific and shared oCGIs (*top:* A-only, B-only, Shared) and species-specific and shared TF peaks (*left*: A-only, B-only, Shared), compared to a null expectation of no association between oCGI turnover and peak turnover. Each 3 x 3 grid shows the results for a specific test examining oCGIs and their overlap with TF peaks in adult liver within a given species pair. Each box in each grid is colored according to the level of enrichment over expectation (teal for CTCF, magenta for HNF4A, purple for HNF6, red for FOXA1, and green for CEBPA) or depletion (gray for all TFs) of genome-wide sites that meet the criteria for that box. The color bars illustrate the level of enrichment or depletion over expectation. The filled upward-pointing triangles denote significant enrichment and open downward-pointing triangles denote significant depletion (q < 0.05, permutation test, BH-corrected; see Fig. S22 and Methods).


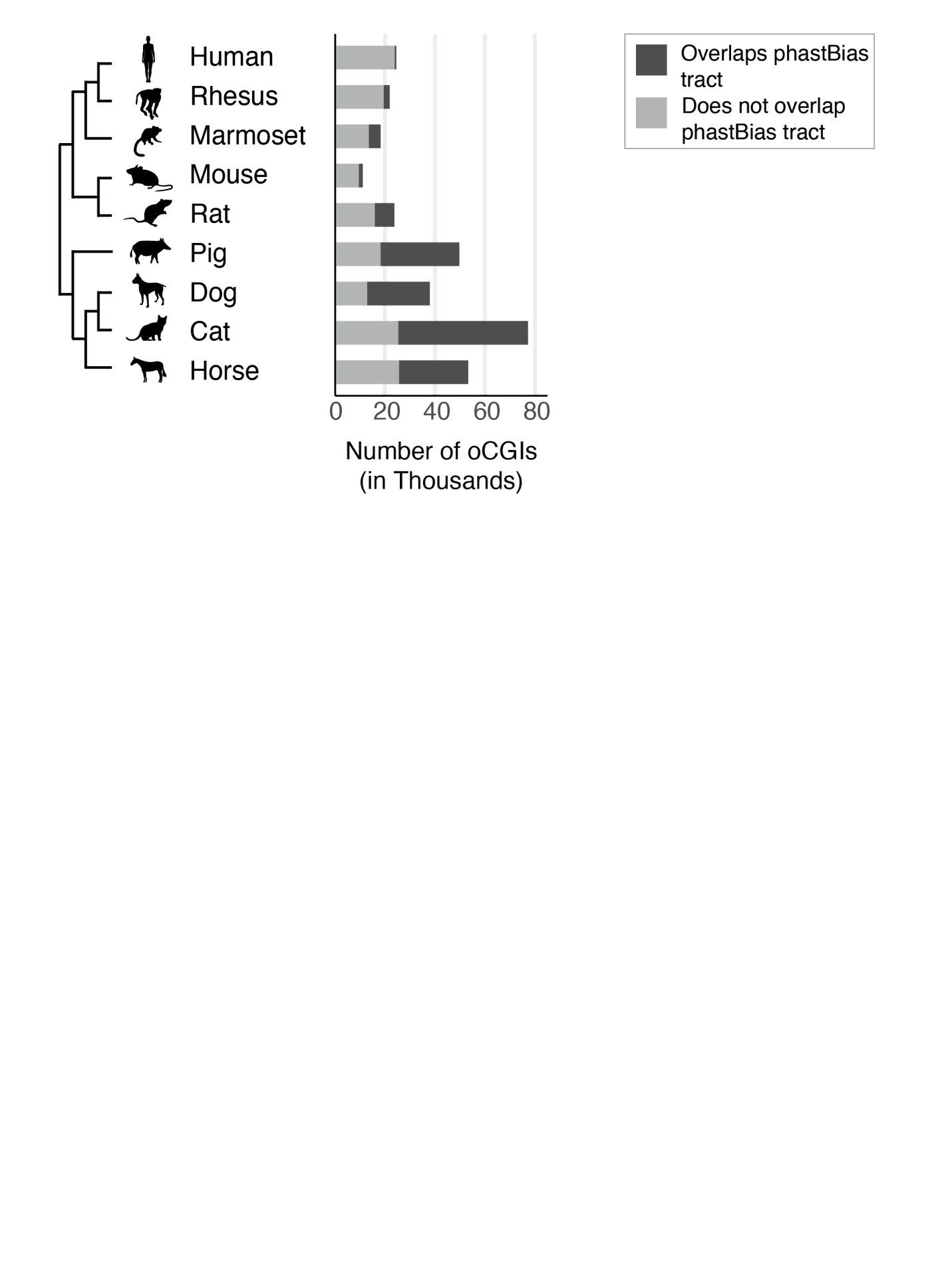


Fig S47. GC-biased gene conversion tracts overlap many oCGIs

Bar plots show the number of oCGIs in each species, with sites overlapping a phastBias tract in dark gray and sites not overlapping a phastBias tract in light gray.


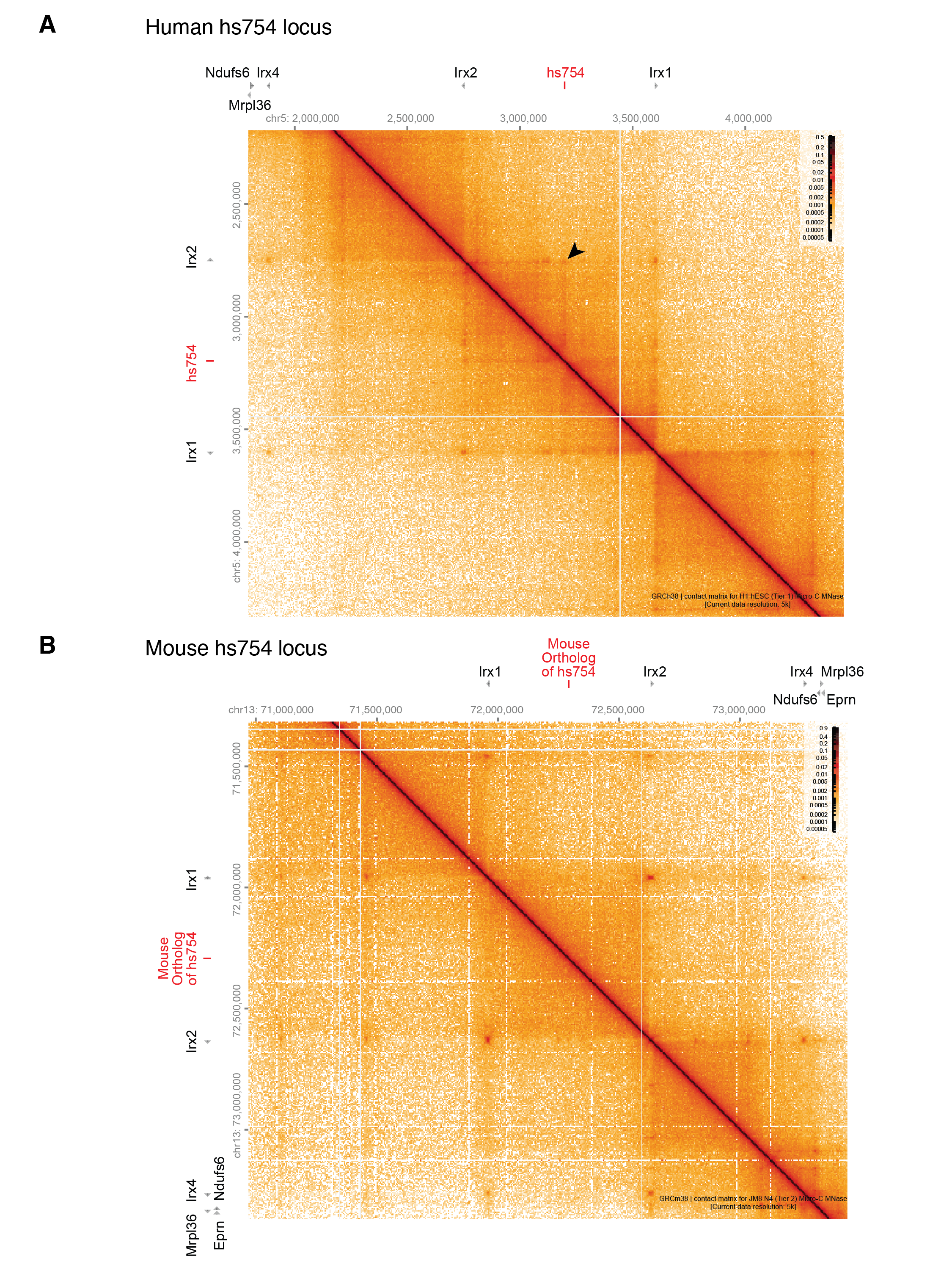


**Fig S48. Hs754 contacts *Irx2* in human embryonic stem cells.**

(A) Micro-C map for the locus surrounding hs754 in human embryonic stem cells (ESCs) at 5-kb resolution [85]. Color indicates contact intensity between a pair of genomic locations. Genomic features (genes and hs754) are indicated above and on the left of the heatmap. The contact between hs754 and *Irx2* is indicated by a black arrow. (B) Micro-C map for the locus surrounding hs754 in mouse ESCs at 5-kb resolution [84]. Data are shown as in panel A, but note that the locus is reversed in mouse genomic coordinates (*Irx2* is downstream of hs754 in mouse compared to upstream in human).


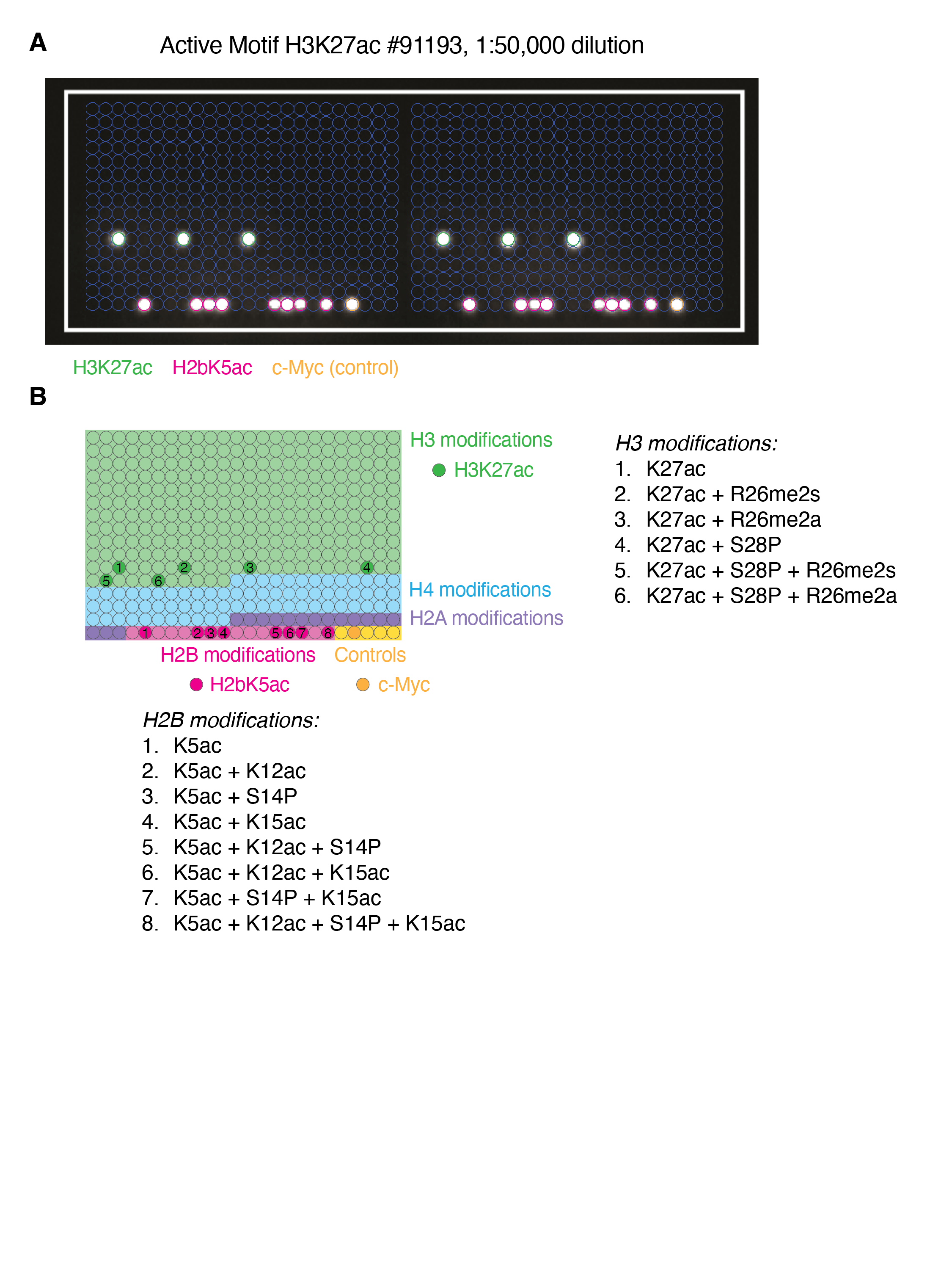


**Fig S49. Specificity of H3K27ac antibody used for humanized mouse ChIP-seq**

(A) Results of an analysis of H3K27ac antibody specificity using the MODified peptide array from Active Motif. Each blue-outlined circle shows the location of a peptide containing one or several posttranslational modifications. White signal shows peptides bound by the antibody of interest. Circles show the locations of peptides containing H3K27ac (green) or H2bK5ac (magenta), or the positive control antigen c-Myc (yellow). (B) Key showing locations of peptides from different histones (green background for histone H3, blue background for histone H4, purple background for histone H2A, pink background for histone H2B, yellow for control antigens). Circles filled with darker green show peptides with H3K27ac, and the additional modifications on these six peptides are listed to the right. Circles filled with darker pink show peptides with H2bK5ac, and the additional modifications on these eight peptides are listed below. Circle filled with darker yellow shows the location of the c-myc control antigen.


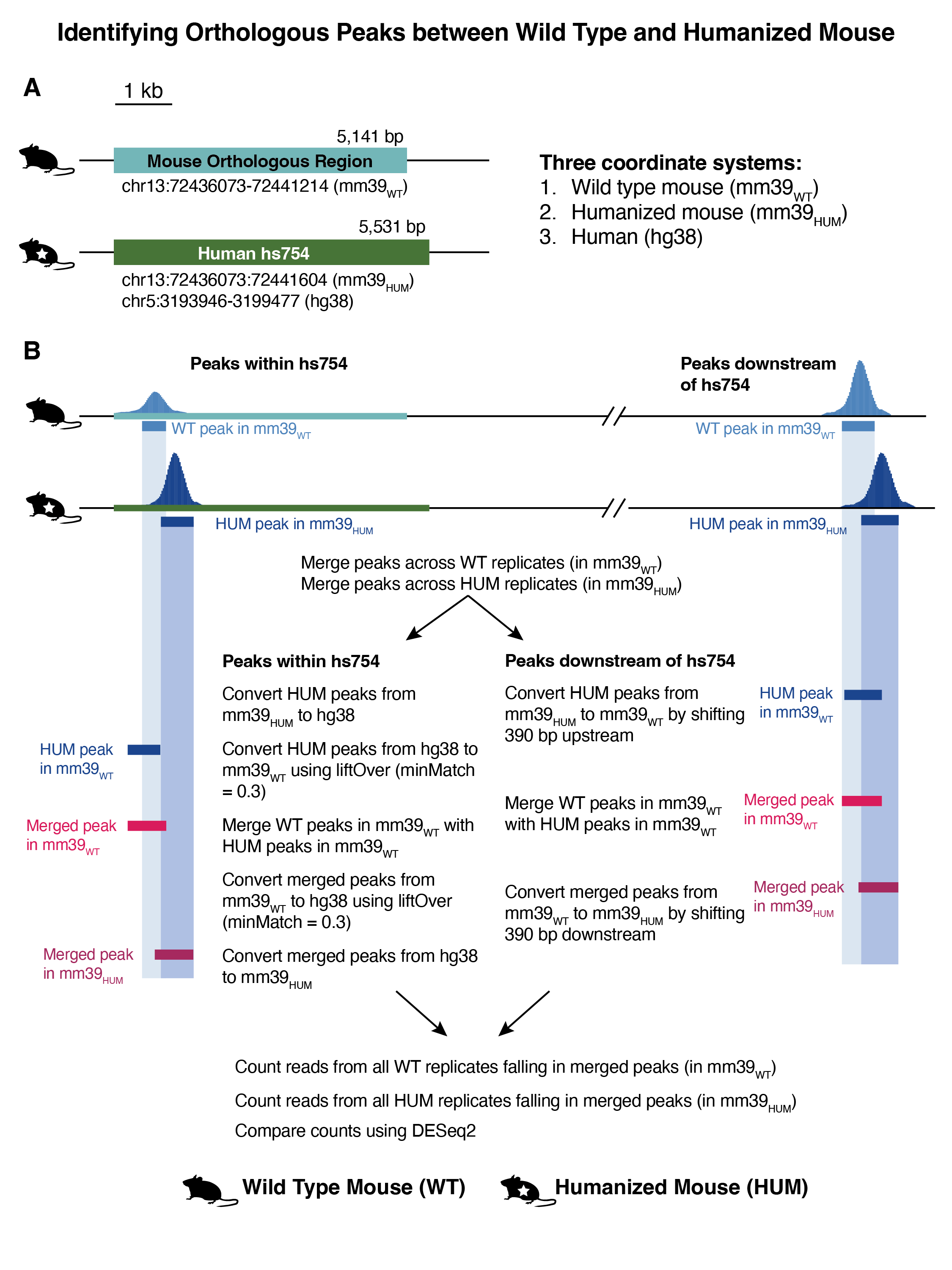


**Fig S50. Converting peak coordinates between wild type and humanized mouse**

(A) Schematic of the hs754 humanized locus and orthologous wild type locus, showing the size difference that requires a special procedure for comparing peaks between the two genotypes. Coordinates in the wild type mouse locus are referred to in mm39, denoted here as mm39_WT_. Coordinates in the humanized mouse locus can be referred to either in humanized mouse coordinates (mm39_HUM_) or human coordinates (hg38). (B) Procedure for comparing peaks between the wild type and humanized mouse loci, either within the humanized locus (*left*) or downstream of it on chromosome 13 (*right*).


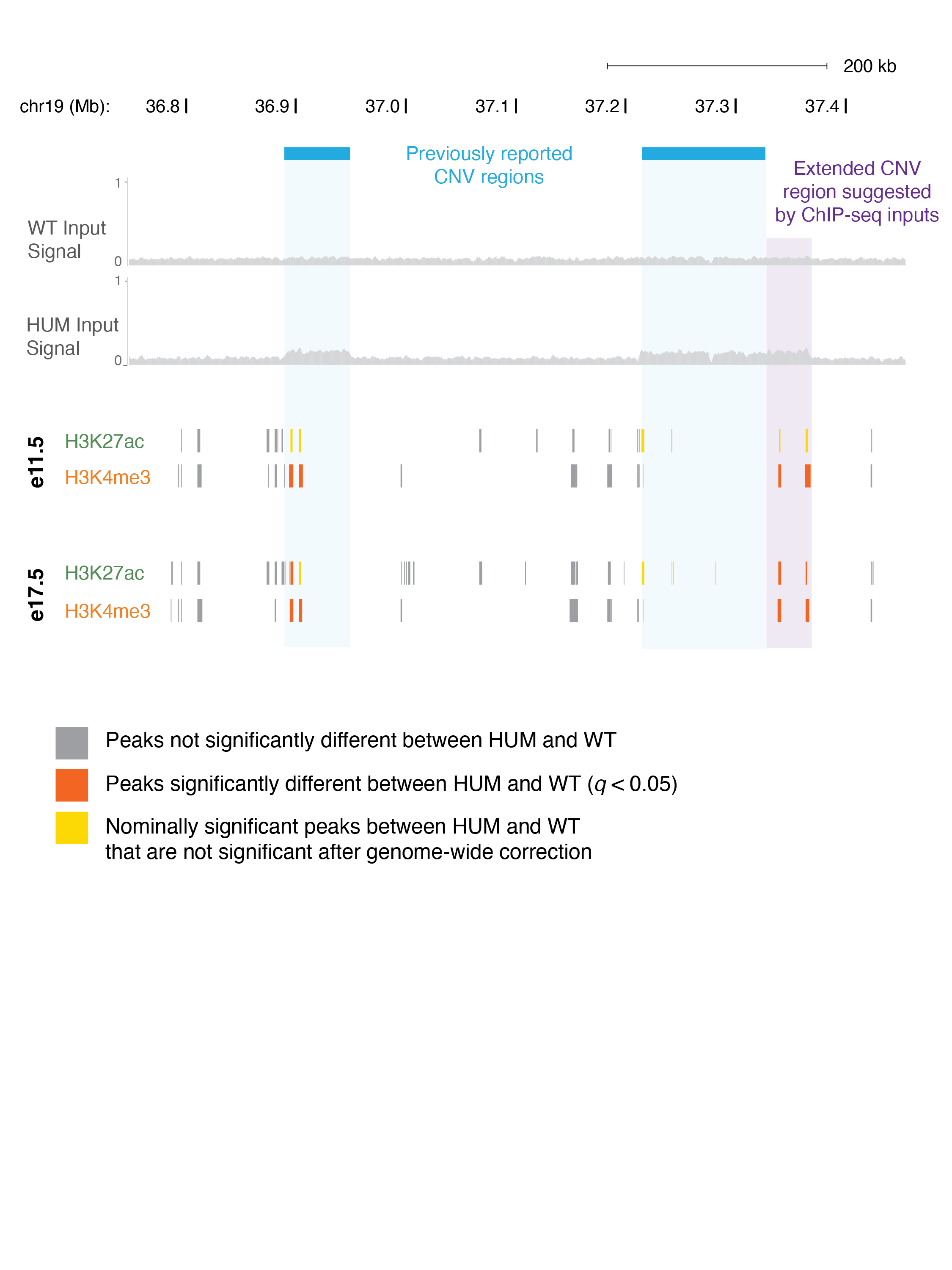


Fig S51. Copy-number variants (CNVs) on chromosome 19

Two genomic intervals on chromosome 19 containing known CNVs [104]. ChIP input signal tracks from this study are shown in gray (from one replicate of wild type (WT) and humanized (HUM) E11.5 diencephalon) and suggest a duplicated signal in these regions (light blue), and in an extended region adjacent to the larger reported region (purple). All differential peaks on chromosome 19 that we called in this study fall within these CNV regions.
