# Supplementary material for: CpG island turnover events predict evolutionary changes in enhancer activity
Source: Genome Biol. 2024 Jun 13;25:156. doi: 10.1186/s13059-024-03300-z (PMC11170920; doi:10.1186/s13059-024-03300-z)
Supplement: Supplementary file 3 — Additional file 3: Review history. [file 13059_2024_3300_MOESM3_ESM.docx]

Review History

**First round of review**

**Reviewer 1**

**Were you able to assess all statistics in the manuscript, including the appropriateness of statistical tests used?**

Yes, and I have assessed the statistics in my report.

**Comments to author:**

In the current manuscript Kocher et al., investigate oCGi turnover between species. They show that oCGis are enriched for regulatory element associated histone modifications more strongly than enhancers without oCGis (Figure 1). Many oCGis are different between at least 2 species (Figure2) and species specific oCGis correlate with species specific histone modifications (most strongly H3K4me3), (Figures 3 and 4). A single predicted species-specific enhancer containing a species specific oCGi is modeled in mouse brain and shows species specific H3K4me3 enrichment (Figure 5). The authors go on to show that species specific oCGis with species specific histone modifications correlate with species specific gene expression differences (Figure 6) and that there are species specific transcription factor binding sites with those associated as well (Figure 7). There's a lot of descriptive analyses in this manuscript and most of the correlative data behaves as expected. As such the authors may consider slimming down the manuscript to improve readability. Overall, I'm not aware of other analysis of evolutionary turnover of CGis in enhancers so this is where the novelty of the manuscript lies. I have several suggestions or questions for the authors to consider.

It wasn't clear to me how oCGi turnover between species is measured. The examples shown in 2A and 3B look believable but what about oCGis that are more closely related say 45% CG content versus 50% CG content. Is there a hard threshold used at 50% CG content and 0.6 over expected ratio or does there need to be minimal CG difference between two orthologous elements as well to be considered species specific. Incorporating such requirement may enable the selection of larger differences and as such increase the effects sizes that are seen.

I was confused by the claim that H3K4me3 is an enhancer associated chromatin mark while its quite clear from the literature that this is associated with promoters and not with enhancers. Its deposition is also a direct result of CGi content which was clearly shown by Adrian Birds lab. Furthermore, work from Stark and others demonstrated that many intergenic enhancers are in fact orphan promoters that can function as enhancers. I think its important to get that distinction right throughout the manuscript.

Related to that I seems that the proto enhancer theory while probably correct in some cases may not be the best explanation for oCGi turnover. Isn't it also plausible that ancient promoters that are part of transposable elements erode promoter function while retaining enhancer function. On that note are CGis more often gained or lost from otherwise constrained enhancers? Or from constrained enhancers in transposable elements?

I'm not sure what the point is of figure 5. Since the entire 5kb enhancer is switched between human and mouse it doesn't day much about CGi involvement as the authors also discuss. As making the mice is significant work, was there more to this element as to why this one was chosen?

Do the authors envision a role for CGi in altered connectivity in their humanized mouse as was shown in ref 70?

Figure 2 is also labeled as Figure 1

**Reviewer 2**

**Were you able to assess all statistics in the manuscript, including the appropriateness of statistical tests used?**

Yes, and I have assessed the statistics in my report.

**Comments to author:**

In this study, Kocher et al. define a class of "orphan" CpG islands (oCGis, non-promoter associated) and examine their contribution to differences in enhancer activity across nine mammalian genomes. Using the Gardiner-Garden and Frommer CpG island definition, their approach identified orthologous and species specific CpG islands. From this, the authors discover a significant number of species-specific oCGIs that are enriched for species specific peaks of enhancer associated histone modifications. Lastly, they demonstrate that an oCGI-containing Human Gain Enhancer gains enhancer-associated histone modifications in a humanized mouse model. The authors propose that oCGIs are a novel class of sequence elements contributing to gene regulatory changes underlying mammalian evolution.

The strength of the study is that it addresses an intriguing question and elusive problem in mammalian genome evolution. The evolutionary depletion of CpG sites in mammalian genomes leading to CpG hotspots, their unique epigenetic regulation, and their contribution to changes in gene regulation is not well understood. The work is extensive and presents a substantial number of analyses to support their conclusions. I would support seeing this type of study published, but I have some reservations regarding the approach and conclusions made as a result. Listed below are points for the authors to consider.

1.	The CpG island definition is reliant on the definition established by Gardiner-Garden and Frommer prior to whole genome sequence availability and has been generalized across species. While this definition has been highly useful for identifying functionally important CpG islands, it is possible that some regions are missed that do not meet these specific criteria. This is especially important when considering the regions identified as species specific. Probabalistic models have been developed to define these regions, accounting for sequence content variation between species (see Mamm Genome. 2009 Sep-Oct;20(9-10):674-80 and Biostatistics. 2010 Jul;11(3):499-514). Can the authors discuss this point as it relates to their findings?

2.	Related to this, CpG dinucleotide counts are shown in Fig. S12 and Fig. 2C, but it would be better to show the Obs/Exp CpG content comparison, considering potential differences in CG content, etc, between genomes.

3.	Can the authors clarify whether the "shared" oCGI definition includes an oCGI defined in one species and shared with ANY species? For example, the way this section currently reads, it appears the "species-specific" definition is for each pair-wise comparison rather than any comparison.

4.	The authors argue that the overlap of oCGIs with histone modification peaks reveals putative enhancers with possible oCGI-dependent functions. For the human oCGIs it should be possible to intersect these with chromHMM and ENCODE ccREs definitions to understand their distribution among these regulatory element definitions. Given the enrichment of H3K4me3, what's the proportion of oCGIs that overlap TSS/Promoter-like regions vs. strong/weak enhancers called by these models?

5.	Considering the H3K4me3 enrichment and its association with transcription start-sites, I wonder if some of the oCGIs may be marking promoters of transposable elements (including species-specific ones), pseudogenes, or non-coding RNAs?

6.	The authors make pretty strong statements throughout the manuscript that oCGIs drive enhancer evolution by either carrying with them TF binding sites or by virtue of the fact that CG content directly recruits chromatin modifiers associated with transcriptional activity. But it seems that these two things cannot really be separated (without genetic manipulation), so this really is an overstatement. I would consider revising references to "transcription-factor independent" mechanisms.

Minor comments:

Throughout the manuscript, beware of inaccurate language - for example, "The 'recruitment' of H3K4me3….are directly mediated by the CpG…..(line 28, pg. 4, para. 2). H3K4me3 is not recruited, rather the enzymes that modify the histones are recruited.

**Reviewer 3**

**Were you able to assess all statistics in the manuscript, including the appropriateness of statistical tests used?**

No, I do not feel adequately qualified to assess the statistics.

**Comments to author:**

The manuscript by Kocher et al. try to demonstrate that the turnover of CpG islands (CGIs) is likely a mechanism driving gene regulatory changes, which could contribute to trait evolution in mammals.

Although some of data are intriguing and will be appreciated by researchers in the relevant fields, I have to say that findings here are not so advanced. Most importantly, the model proposed in Figure 8 seems to be mostly based on author's hypothesis and not supported by their data. I have the following comments for the authors.

Major points:

1. It is well accepted that CxxC-domain containing proteins such as CFP1, KDM2B, TET1 and etc. recognize CGIs and modify eigenomes including H3K4me, H3K36me, and DNA methylation. Therefore, it is not surprising to see orphan CGIs (oCGIs) accept histone modifications that authors observed. Especially, H3K4me is induced at almost all CGIs autonomously. Rather, I think that it will be critically important when and how oCGIs are activated (becoming active enhancers) during for example development. Because authors did not look into DNA methylation status or repressive histone modifications such as H3K27me3, it is not clear in terms of transition of the enhancer activity. It has been reported that CTCF binding could be affected by DNA methylation. The difference in CTCF binding among species might be the different DNA methylation pattern.

2. As I pointed above, CGIs accept histone modifications autonomously. Therefore, it is not clear whether histone modifications in oCGIs can predict enhancer activity unless authors examine Promoter-enhancer looping by 4C or Hi-C type of experiments.

**Authors Response**

**Point-by-point responses to the reviewers’ comments:**

Reviewer #1: In the current manuscript Kocher et al., investigate oCGi turnover between species. They show that oCGis are enriched for regulatory element associated histone modifications more strongly than enhancers without oCGis (Figure 1). Many oCGis are different between at least 2 species (Figure2) and species specific oCGis correlate with species specific histone modifications (most strongly H3K4me3), (Figures 3 and 4). A single predicted species-specific enhancer containing a species specific oCGi is modeled in mouse brain and shows species specific H3K4me3 enrichment (Figure 5). The authors go on to show that species specific oCGis with species specific histone modifications correlate with species specific gene expression differences (Figure 6) and that there are species specific transcription factor binding sites with those associated as well (Figure 7). There's a lot of descriptive analyses in this manuscript and most of the correlative data behaves as expected. As such the authors may consider slimming down the manuscript to improve readability. Overall, I'm not aware of other analysis of evolutionary turnover of CGis in enhancers so this is where the novelty of the manuscript lies. I have several suggestions or questions for the authors to consider.

We thank the reviewer for their feedback on the manuscript. We have edited the text to improve readability throughout. For example, we shortened passages on pages 6, 13, 21, 26, 36, and the legend for Figure 4A-B. In addition, we have combined Figures 4 and 5 into a single figure by moving Fig. 4C and Fig. 5C to the supplement.

It wasn't clear to me how oCGi turnover between species is measured. The examples shown in 2A and 3B look believable but what about oCGis that are more closely related say 45% CG content versus 50% CG content. Is there a hard threshold used at 50% CG content and 0.6 over expected ratio or does there need to be minimal CG difference between two orthologous elements as well to be considered species specific. Incorporating such requirement may enable the selection of larger differences and as such increase the effects sizes that are seen.

We did perform the original analysis with a hard threshold at 50% GC content and 0.6 CpG Obs/Exp ratio. We appreciate the reviewer’s suggestion and agree that setting a minimal CpG difference requirement for species comparisons could reveal sites with larger differences. To examine this, we evaluated several minimal difference thresholds (0.1, 0.3, and 0.5) in the CpG Obs/Exp ratio between species pairs. For example, if Species A had a ratio of 0.6 Obs/Exp CpGs at a specific oCGI, then a 0.1 difference requirement means that Species B was required to have a ratio of 0.5 or less for that site to be called as a species-specific oCGI.

In the revised Fig. S30A we present how imposing these thresholds reduce the numbers of sites identified as species-specific within a species pair. Shared sites remain unchanged because to be initially called as a shared oCGI, the oCGI was called in both species and therefore met the CpG threshold in the CGI definition.

We used these new CGI sets to perform histone modification enrichment analysis as before, using histone modification data from the adult brain and liver. In general, we found that increasing the minimal difference requirement results in stronger enrichments for species-specific oCGIs with species-specific histone modification peaks. It also results in stronger depletions for species-specific oCGIs with peaks specific to the other species in the pair and with shared peaks. In Fig. S30B we present the results for a subset of species pairs and tissues using three minimal difference requirements in CpG Obs/Exp ratio, in comparison to the previous results with no requirement. In some cases several of the conditions lose statistical significance using a difference threshold of 0.3 or 0.5, likely due to the smaller sample sizes available at this more stringent level.

We have included a description of this analysis on the revised page 17 (line 327). Because these results are consistent with the trends from our original results, we have retained the figures presenting the enrichment analyses using our original criteria in the revised manuscript.

I was confused by the claim that H3K4me3 is an enhancer associated chromatin mark while its quite clear from the literature that this is associated with promoters and not with enhancers. Its deposition is also a direct result of CGi content which was clearly shown by Adrian Birds lab. Furthermore, work from Stark and others demonstrated that many intergenic enhancers are in fact orphan promoters that can function as enhancers. I think its important to get that distinction right throughout the manuscript.

We recognize the notion that H3K4me3 is specific for promoters is widespread in the literature (e.g., Heintzman *et al Nature* 2007, PMID: 17277777), and its presence is often used to identify promoters, and exclude enhancers, using epigenetic data (e.g, Villar *et al Cell* 2015, PMID: 25635462; Roller *et al Genome Biology* 2021, PMID: 33602314). However, there is now evidence from multiple studies that H3K4me3 is not exclusive to promoters, but is also associated with enhancers. In particular, higher levels of H3K4me3 correlate with higher levels of enhancer RNA transcription, another feature associated with enhancer activity, and with RNA Pol II occupancy (Pekowska *et al EMBO Journal* 2011, PMID: 21847099; Core *et al Nature Genetics* 2014, PMID: 25383968; Henriques *et al Genes & Development* 2017, PMID: 29378787).

In line with this changing view of H3K4me3 at enhancers, the ENCODE project has recently adopted a strategy for defining enhancers and promoters which takes into account not only histone modifications, but also genomic proximity to an annotated transcription start site (TSS). We discuss this approach below in response to point 4 from Reviewer #2, and provide a schematic of the ENCODE criteria in the revised Fig. S6A. In this approach, distal enhancers are allowed to overlap H3K4me3 peaks. We have also added text highlighting this on page 8 (line 186).

Finally, we also performed an analysis for this study supporting that H3K4me3 (and H3K4me di-and mono-methylation) correlates with enhancer activity in a LacZ reporter assay (revised Fig. S3). We discuss these results further in our response to point 1 from Reviewer #3.

Related to that I seems that the proto enhancer theory while probably correct in some cases may not be the best explanation for oCGi turnover. Isn't it also plausible that ancient promoters that are part of transposable elements erode promoter function while retaining enhancer function.

It is certainly possible that some enhancers are born from the erosion of ancient promoters, including in transposable elements. We excluded annotated repeats from our analysis by only calling oCGIs on repeat-masked genomes, and furthermore by excluding species-specific oCGIs whose orthologs in the other species in a pair have more than 20% repeat content. Therefore we are not able to identify recently-inserted transposons, although our oCGI sets could contain very ancient transposable elements that have decayed enough that we cannot recognize them as repeats. We now mention this possibility in the revised Discussion (page 35, line 626).

On that note are CGis more often gained or lost from otherwise constrained enhancers? Or from constrained enhancers in transposable elements?

To address this question, we compared the number of observed oCGI gains and losses on terminal branches of the phylogeny shown in Fig. 1A by comparing each genome to a sister species and an outgroup. For example, using human as a terminal branch, we identified all sites with a gained oCGI in human (present in human, absent in rhesus and marmoset) or with a lost oCGI in human (absent in human, present in rhesus and marmoset). We performed this analysis separately for sites overlapping and not overlapping a phastCons element.

The results are presented in Fig. S21 and are discussed in the text on page 14. In general, we identify more gain events than loss events. However, more loss events are identified in sites with a phastCons element than sites without. In most cases (except human), the number of gained oCGIs is larger than the number of lost oCGIs. This bias towards gains may be because they are easier to detect: to identify a gain, an oCGI must be present in only one species, whereas to identify a loss, an oCGI must be present in two species.

We are unable to address the question about constrained regions of transposable elements because we filtered all annotated repeats out of our analysis, due to the difficulty of identifying orthologous sequences for these sites across species.

I'm not sure what the point is of figure 5. Since the entire 5kb enhancer is switched between human and mouse it doesn't day much about CGi involvement as the authors also discuss. As making the mice is significant work, was there more to this element as to why this one was chosen?

We agree with the reviewer that further experiments would be necessary to understand the contribution of the oCGI versus other components of the enhancer to the gained activity of hs754 in our humanized mouse model. Initially we chose to make a humanized mouse model for this enhancer based on evidence of its human-specific activity during brain development that has potential relevance for human brain evolution (Reilly *et al Science* 2015, PMID: 25745175; Fig. S36). In working to understand the molecular mechanisms underlying its modified enhancer activity, we noticed the difference in oCGI content between human and mouse. This observation inspired the larger-scale analysis of oCGI turnover and enhancer activity presented in the rest of the study. We would like to include the mouse model in the study, as it may be a resource for others interested in studying mechanisms of enhancer evolution generally, and the role of oCGI turnover specifically, in an *in vivo* system. As noted by the reviewer, we do discuss the limitations of our results (now on page 34 in the revised manuscript). In light of these limitations and the reviewer’s recommendation to streamline the manuscript, we have reduced the main figures

devoted to the humanized mouse model, moving our original Figures 4B and 5C into the supplement (as Figure S36 & S38) and moving the original Fig. 5A-B into a single panel, Figure 4C.

Do the authors envision a role for CGi in altered connectivity in their humanized mouse as was shown in ref 70?

Reference 73 (now reference 82) identified hs754 as a “poised enhancer” in human ESCs, but not mouse ESCs. This was based on the chromatin state in human ESCs compared to mouse ESCs, specifically the combination of open chromatin and H3K27me3. This study did not examine looping of hs754 to potential target genes. However, it has recently been shown that several other poised enhancers are tethered to their target promoters in ESCs, and that these loops are mediated by Polycomb-group proteins binding to CpG islands in both the enhancer and promoter (reference 79, formerly reference 70 as mentioned by the reviewer). Because hs754 is a poised enhancer in human but not in mouse, we examined its contacts with target genes in human and mouse ESCs using published Micro-C data (Hsieh *et al Molecular Cell* 2020, PMID: 32213323; Krietenstein *et al Molecular Cell* 2020, PMID: 32213324). We find a loop between hs754 and *Irx2* in human ESCs, but not in mouse ESCs (Fig. S48), in agreement with predictions based on promoter loops present at other poised enhancers. We have incorporated this result into the manuscript on page 38.

Genetic ablation of other poised enhancers alters the activation of their target genes during early development (Cruz-Molina *et al Cell Stem Cell* 2017, PMID: 28285903, Crispatzu *et al Nature Comm* 2021, PMID: 34272393). We hypothesize that hs754 may affect the activation dynamics of *Irx2* in human compared to mouse. However, we did not perform experiments at such early time points to address this. *Irx2* expression is first detected in the developing mouse hindbrain at E8.5, which is 3 embryonic days before our earliest experiments (Bosse *et al Mech Dev* 1997, PMID: 9486539). It is possible that humanization of our enhancer alters the timing, level, or spatial extent of *Irx2* expression in tissues or at time points we did not examine here. We believe these experiments are outside the scope of the current study but do introduce this hypothesis in the Discussion.

Figure 2 is also labeled as Figure 1
We have corrected this error.

Reviewer #2: In this study, Kocher et al. define a class of "orphan" CpG islands (oCGis, non-promoter associated) and examine their contribution to differences in enhancer activity across nine mammalian genomes. Using the Gardiner-Garden and Frommer CpG island definition, their approach identified orthologous and species specific CpG islands. From this, the authors discover a significant number of species-specific oCGIs that are enriched for species specific peaks of enhancer associated histone modifications. Lastly, they demonstrate that an oCGI-containing Human Gain Enhancer gains enhancer-associated histone modifications in a humanized mouse model. The authors propose that oCGIs are a novel class of sequence elements contributing to gene regulatory changes underlying mammalian evolution.

The strength of the study is that it addresses an intriguing question and elusive problem in mammalian genome evolution. The evolutionary depletion of CpG sites in mammalian genomes leading to CpG hotspots, their unique epigenetic regulation, and their contribution to changes in gene regulation is not well understood. The work is extensive and presents a substantial number of analyses to support their conclusions. I would support seeing this type of study published, but I have some reservations regarding the approach and conclusions made as a result. Listed below are points for the authors to consider.

1. The CpG island definition is reliant on the definition established by Gardiner-Garden and Frommer prior to whole genome sequence availability and has been generalized across species. While this definition has been highly useful for identifying functionally important CpG islands, it is possible that some regions are missed that do not meet these specific criteria. This is especially important when considering the regions identified as species specific. Probabalistic models have been developed to define these regions, accounting for sequence content variation between species (see Mamm Genome. 2009 Sep-Oct;20(9-10):674-80 and Biostatistics. 2010 Jul;11(3):499-514). Can the authors discuss this point as it relates to their findings?

We thank the reviewer for their feedback on the manuscript. We appreciate this question about CGIs defined by probabilistic models, and we agree that the choice of criteria in defining CpG islands is important. We describe our rationale for choosing the CGI annotations we used below, and then the additional analyses we conducted to compare the number of CGIs identified using our criteria compared to a probabilistic model.

Early in our study, we initially chose to use the CGI annotations available from the UCSC genome browser. However, we realized these were defined using very restrictive criteria that impose additional requirements on the Gardiner-Garden and Frommer definition that only retain the strongest, most CpG-dense CGIs. This was chosen intentionally in the early 2000s so that the number of CGIs would more closely match the number of genes/promoters in the genome, and is described on the Genome Browser wiki:

<https://genomewiki.ucsc.edu/index.php/CpG_Islands.>

For our study, we chose to use more permissive CpG island annotations defined using criteria that do not impose these additional requirements, especially because oCGIs tend to be shorter and contain fewer CpGs than promoter-associated CGIs. Therefore, we used a more permissive algorithm developed by the UCSC Genome Browser annotation team (described on the wiki page linked above) that more closely matches the Gardiner-Garden and Frommer definition. We discuss

our rationale for this choice in the Methods section and in the revised main text on page 7 (line 137). We also assessed these differences and present the results in Fig. S2A (*left*), with our oCGIs called “UCSC permissive oCGIs” and the standard set called “UCSC standard oCGIs.”

To address the reviewer’s comment, we decided to compare both of these CGI annotation sets to CGIs (here called “HMM oCGIs”) defined by a probabalistic model described in the 2010 study by Wu *et al*, and which are available here: <https://www.haowulab.org/software/makeCGI/index.html.>

We retrieved HMM oCGIs for the following species: human, rhesus macaque, mouse, rat, dog, and horse. In order to facilitate comparisons with our CGIs, we used liftOver (minMatch=0.8) to map the HMM oCGIs to the genome builds we used in our study (rheMac2 to rheMac10, mm10 to mm39, rn4 to rn7, canFam2 to canFam6, and equCab2 to equCab3). As we did for our CGIs, we removed HMM oCGIs that overlapped annotated promoters and gene bodies, including for pseudogenes and ncRNAs. We then assessed the overlap of oCGIs from each annotation strategy. Both UCSC permissive oCGIs and HMM oCGIs identify many more sites than UCSC standard oCGIs (Fig. S2A, *left* and *middle*). Additionally, there is overlap between UCSC permissive oCGIs and HMM oCGIs, but both strategies identify a large number of sites not identified by the other (Fig. S2A, *right*).

However, there is a fundamental difference between the UCSC permissive oCGIs and the HMM oCGIs: our UCSC permissive oCGIs were called from hard-masked genomes, unlike the HMM oCGIs called on unmasked genomes. We chose to exclude oCGIs called in repeats in our analysis, because of the challenges in reliably identifying orthologous sequences for repeats across species. Small segments of repeat-masked bases (i.e., marked as N in the alignments) can still be found in our oCGI sets when the rest of the interval still meets the Gardiner-Garden & Frommer criteria, but this is not common. However, we found that HMM oCGIs are present within repeats. We calculated the percentage of repeat-masked bases in each oCGI, and present the distributions for all three oCGI annotations we considered in Fig. S2B. When we remove oCGIs with >50% repeat-masked bases, the number of sites identified only in the HMM oCGI sets substantially decreases (Fig. S2C), while sites only identified by the UCSC permissive oCGIs are retained.

Based on these results, we are confident that the UCSC permissive criteria are more sensitive at identifying oCGIs in the non-repeat genome, and therefore can more accurately identify species-specific sites. We describe this analysis in further detail on page 7.

2. Related to this, CpG dinucleotide counts are shown in Fig. S12 and Fig. 2C, but it would be better to show the Obs/Exp CpG content comparison, considering potential differences in CG content, etc, between genomes.

We agree that visualizing Obs/Exp CpG ratio would be valuable, and we now present these results in the revised Fig. S17 and discuss them on page 13 (line 261). We generally see that shared oCGIs have a higher ratio of Obs/Exp CpGs than A-only or B-only oCGIs (significant in 29 out of 56 tests). In a small number of species pairs, shared oCGIs have lower ratios of Obs/Exp CpGs than A-only or B-only oCGIs (significant in 7 out of 56 tests). In all cases, the differences are small in magnitude compared to the other measures we consider (CpG number and CpG length, as shown

in Figures S15 and S16). For these measures, shared oCGIs always have a higher number of CpGs and are longer compared to species-specific oCGIs.

We would like to include CpG dinucleotide counts in main Figure 2C. We would like to note that the comparisons presented the revised Figs. S15-S17 and Fig. 2C are done within a single genome, so concerns about differences in GC content between genomes are not applicable. Furthermore, concerns about GC content across species are typically more important when looking across diverse vertebrates, rather than just within placental mammals as in our study. For example, the human genome has an overall mean GC content of 41%, compared to 42% in the mouse genome (Mouse Genome Sequencing Consortium *Nature* 2002, PMID: 12466850). We originally chose to show CpG dinucleotide counts instead of other metrics (e.g. length, CpG density, Obs/Exp ratio etc) because we believed that the number of CpG dinucleotides would best reflect the ability of the element to recruit CpG-dependent mechanisms that modify chromatin, as the number of CpG dinucleotides may scale proportionally to the number of CpG-binding proteins that can be recruited (Hughes *et al Biochim Biophys Acta Gene Regul Mech* 2020, PMID: 32360393).

3. Can the authors clarify whether the "shared" oCGI definition includes an oCGI defined in one species and shared with ANY species? For example, the way this section currently reads, it appears the "species-specific" definition is for each pair-wise comparison rather than any comparison.

The shared oCGIs are always defined within the context of one pair-wise comparison. We have clarified this in the text (page 13, line 250).

4. The authors argue that the overlap of oCGIs with histone modification peaks reveals putative enhancers with possible oCGI-dependent functions. For the human oCGIs it should be possible to intersect these with chromHMM and ENCODE ccREs definitions to understand their distribution among these regulatory element definitions. Given the enrichment of H3K4me3, what's the proportion of oCGIs that overlap TSS/Promoter-like regions vs. strong/weak enhancers called by these models?

We overlapped our oCGIs with ENCODE cCREs in order to understand the distribution of oCGIs across regulatory element definitions. This was possible for human oCGIs using human cCREs, and for mouse oCGIs using mouse cCREs.

We would like to highlight that the ENCODE definitions rely not on histone modifications alone, but rather on a combination of histone modifications and distance from annotated TSSs (see our schematic in Fig. S6A, which is modeled after Figure 2 of the “About” tab at <https://screen.encodeproject.org/)>. Notably, proximal and distal enhancers are allowed to overlap H3K4me3 peaks. This strategy reflects mounting evidence that both enhancers and promoters can be marked by H3K4me3, reflecting transcription and RNA II Pol occupancy that is common at enhancers, particularly highly active ones (Pekowska *et al EMBO Journal* 2011, PMID: 21847099; Core *et al Nature Genetics* 2014, PMID: 25383968; Henriques *et al Genes & Development* 2017, PMID: 29378787). We also discuss this in response to one of Reviewer #1’s points above.

We find that human and mouse oCGIs overlap mostly distal enhancer-like elements (dELS), with some overlapping proximal enhancer-like elements (pELS). We present the results in Fig. S6B and discuss these on page 8 (line 182).

5. Considering the H3K4me3 enrichment and its association with transcription start-sites, I wonder if some of the oCGIs may be marking promoters of transposable elements (including species-specific ones), pseudogenes, or non-coding RNAs?

There may be many CGIs in transposable element promoters, decaying pseudogene promoters, and non-coding RNA promoters, and their contribution to gene regulation is an open question. However, the strategy we built for our study prevents us from addressing it, as we have only analyzed oCGIs that do not overlap these features. Specifically, we filtered out any oCGI that overlaps an annotated pseudogene or non-coding RNA (either the gene body or the promoter, defined as 2 kb upstream of the TSS – see Methods). Additionally, we defined oCGIs using repeat-masked genomes, so no oCGIs should contain annotated repeats such as transposable elements. Ancient repeats that have since decayed beyond recognition could still be present in the oCGIs we analyzed, and we address this in the Discussion (page 35, line 626).

6. The authors make pretty strong statements throughout the manuscript that oCGIs drive enhancer evolution by either carrying with them TF binding sites or by virtue of the fact that CG content directly recruits chromatin modifiers associated with transcriptional activity. But it seems that these two things cannot really be separated (without genetic manipulation), so this really is an overstatement. I would consider revising references to "transcription-factor independent" mechanisms.

We agree that deconvoluting the relative contribution of TF binding sites versus increased CpG content in gained oCGIs that alter enhancer activity will require extensive experimental studies. We have reviewed the instances where we have referred to transcription-factor independent mechanisms in the manuscript.

On page 4, we state that “Several mechanisms link CGIs with transcription-factor independent recruitment of chromatin modifiers.” This statement is in reference to the experimental studies cited in the rest of the paragraph that describe known TF-independent mechanisms, so we believe it is warranted here.

On page 5, we have modified the text as follows to acknowledge the uncertainty the reviewer raises: “The recruitment of H3K4me3-depositing enzymes and consequently all of these downstream effects are mediated by the CpG dinucleotides within CGIs and therefore could be independent of transcription factor binding events.”

We also refer to the difficulty in separating TF-driven and CpG-driven mechanisms in the revised Discussion (page 37): “Deconvoluting the relative contributions of CpG dinucleotides and transcription factor bindings sites to enhancer activity will require further experimental perturbations, as described in a recent study (71).”

Minor comments:

Throughout the manuscript, beware of inaccurate language - for example, "The 'recruitment' of H3K4me3....are directly mediated by the CpG.....(line 28, pg. 4, para. 2). H3K4me3 is not recruited, rather the enzymes that modify the histones are recruited.

Thank you for pointing out this error, we have revised the text on page 4 (now page 5) accordingly: “The recruitment of H3K4me3-depositing enzymes and consequently all of these downstream effects are mediated by the CpG dinucleotides within CGIs and therefore could be independent of transcription factor binding events.”

Reviewer #3: The manuscript by Kocher et al. try to demonstrate that the turnover of CpG islands (CGIs) is likely a mechanism driving gene regulatory changes, which could contribute to trait evolution in mammals.

Although some of data are intriguing and will be appreciated by researchers in the relevant fields, I have to say that findings here are not so advanced. Most importantly, the model proposed in Figure 8 seems to be mostly based on author's hypothesis and not supported by their data. I have the following comments for the authors.

We address the reviewer’s specific comments below. In response to the reviewer’s mention of the model in revised Figure 7 (formerly Figure 8), we would like to note that the model we describe in the Discussion is a hypothetical model that we derived based on our results and is presented as such. We have modified the language presenting it to further emphasize this: “Given these results, we propose a hypothetical model...” (page 35, line 622).

Major points:

1.	It is well accepted that CxxC-domain containing proteins such as CFP1, KDM2B, TET1 and

etc. recognize CGIs and modify eigenomes including H3K4me, H3K36me, and DNA methylation. Therefore, it is not surprising to see orphan CGIs (oCGIs) accept histone modifications that authors observed. Especially, H3K4me is induced at almost all CGIs autonomously. Rather, I think that it will be critically important when and how oCGIs are activated (becoming active enhancers) during for example development. Because authors did not look into DNA methylation status or repressive histone modifications such as H3K27me3, it is not clear in terms of transition of the enhancer activity. It has been reported that CTCF binding could be affected by DNA methylation. The difference in CTCF binding among species might be the different DNA methylation pattern.

When we began our study, we shared the reviewer’s concern that, because CGIs accept histone modifications autonomously, these modifications may not be predictive of enhancer activity at oCGIs. In order to address this concern, we performed the analysis included in the original manuscript as Table S1 and Fig. S2 (now the revised Fig. S3), in which we used experimentally-demonstrated enhancer activity in LacZ reporter assays to validate the predictive power of histone modifications for enhancer activity. This analysis is described in the text (page 7, line 150). Briefly,

we collected all sequences tested by the VISTA Enhancer Browser and sorted them based on their overlap with: 1) histone modification peaks identified using ChIP-seq in developing mouse tissues (ENCODE), and 2) mouse oCGIs (Fig. S3A). We then asked whether sequences enriched for each histone modification were more likely to be active in the LacZ reporter assay compared to sequences that were not enriched for the histone modification. Crucially, we asked this question for sequences both with and without oCGIs to determine if the histone modifications were predictive in both cases.

We found that H3K27ac marking is similarly predictive of enhancer activity for enhancers containing oCGIs and enhancers not containing oCGIs (Fig. S3B, first row). H3K4me3 marking was predictive for enhancer activity without oCGIs but not enhancers with oCGIs (Fig. S3B, second row), although the overlap of H3K4me3 peaks with VISTA elements was very small and limited the power of our tests. The marks H3K4me2 and H3K4me1 (Fig. S3B, third and fourth rows) were also predictive for enhancers with and without oCGIs, but only reached statistical significance for enhancers with oCGIs in one sample (H3K4me1 in hindbrain), possibly also due to small sample sizes.

We believe that this analysis shows that histone modifications are predictive of enhancer activity for enhancers containing oCGIs. We have revised the text reporting this analysis in order to highlight this conclusion (page 7-8).

We considered the reviewer’s suggestion to use DNA methylation as an additional measure of enhancer activation. However, we think that this approach would not answer the question being posed by the reviewer. The central concern is that because CGIs autonomously accept histone modifications, the histone modifications may not be a good measure of enhancer activity, and that other measures like loss of DNA methylation could be better. However, any histone modifications recruited autonomously to the CGIs would already be associated with unmethylated CpGs, due to the fact that the CXXC-domain proteins that mediate histone modifier recruitment bind to unmethylated CpGs (Thomson *et al Nature* 2010, PMID: 20393567; Denissov *et al Development* 2014, PMID: 24423662). If we were to find an association between a histone modification and loss of DNA methylation, we could not distinguish between: 1) functional activation of the enhancer causing both histone modification recruitment and DNA de-methylation, and 2) DNA de-methylation associated with the oCGI causing autonomous recruitment of histone modifications without the enhancer gaining activating function. Therefore, we find that our previous approach using LacZ data is a more appropriate strategy, since it incorporates an orthogonal measure of enhancer activation (activity in a LacZ reporter assay) that is not implicated in the molecular mechanisms associated with oCGI activation like DNA methylation is.

Finally, we agree with the reviewer that differences in DNA methylation may impact the binding of methylation-sensitive transcription factors like CTCF, since the gain and loss of oCGIs should correlate with gain or loss of DNA methylation. We have added this to the Discussion on page 36.

2. As I pointed above, CGIs accept histone modifications autonomously. Therefore, it is not clear whether histone modifications in oCGIs can predict enhancer activity unless authors examine Promoter-enhancer looping by 4C or Hi-C type of experiments.

Our analysis using LacZ reporter activity as an orthogonal readout of enhancer activity supports that histone modifications in oCGIs are predictive of enhancer activity. However, to further investigate this question, we used enhancer-promoter looping as another orthogonal readout as suggested by the reviewer. We first collected ENCODE cCREs, specifically those annotated as “distal enhancer-like sequences (dELS),” and sorted them based on their overlap with 1) four histone modifications in E14.5 mouse liver, and 2) mouse oCGIs (see the revised Fig. S4A). We then counted the number of interactions for each enhancer identified in a promoter capture HiC experiment performed in E14.5 mouse liver (Schoenfelder *et al Genome Research* 2015, PMID: 25752748).

We find that cCREs that overlap a histone modification peak exhibit more promoter interactions compared to cCREs that do not overlap a peak, and that this holds for cCREs both with and without oCGIs (Fig. S4B). We find this association between enhancer-promoter interactions and histone modification peaks for all of the histone modifications included in our study, suggesting that all of them are predictive of enhancer activity. This includes H3K4me3, which is most tightly associated with oCGI-driven autonomous recruitment. We have incorporated this result into the text (page 7­8, starting line 150; Methods text on page 44, line 785).

These results provide another orthogonal measure of enhancer activation and suggest that histone modifications are predictive of enhancer activity, for putative enhancers that both do and do not contain oCGIs. These results are consistent with previous work has found that oCGI-containing enhancers have higher strength interactions than enhancers without oCGIs (Bell & Vertino *Epigenetics* 2017, PMID: 28448736). We reference this paper on page 4 in the revised manuscript.

**Second round of review**

**Reviewer #1:**

The authors have effectively addressed al of my suggestions. I support publication.

**Reviewer #2:**

Thank you to the authors for addressing my concerns. The authors have made a substantial effort to provide reasonable responses to my suggestions and have provided extensive additional analysis where needed.

**Reviewer #3:**

In the revised manuscript, authors addressed most of concerns raised by three reviewers. I am also convinced with the revision including LacZ reporter assays. I also appreciate with text changes not to overstate the results and discussions.

One minor comment is that at line 90-91, as far as my understanding, the description is not correct. CFP1 is a subunit of the SETD1A/B histone methyltransferases, and MLL2 is a member of the MLL complex. Authors may need to check the literature (PMID: 23934151) to edit the manuscript.
